# Electron Transfer-Induced Coupling of Haloarenes to Styrenes and 1,1-Diphenylethenes Triggered by Diketopiperazines and Potassium *tert-*Butoxide

**DOI:** 10.3390/molecules20021755

**Published:** 2015-01-22

**Authors:** Eswararao Doni, Shengze Zhou, John A. Murphy

**Affiliations:** WestCHEM, Department of Pure and Applied Chemistry, University of Strathclyde, 295 Cathedral Street, Glasgow G1 1XL, UK; E-Mails: eswararao.doni@strath.ac.uk (E.D.); shengze_zhou@hotmail.com (S.Z.)

**Keywords:** electron transfer, coupling, haloarenes, potassium *tert*-butoxide, diketopiperazine

## Abstract

The coupling of haloarenes to styrenes and 1,1-diarylethenes has been achieved with potassium *tert*-butoxide in the presence of *N,N'*-dialkyldiketopiperazines. In contrast to previously reported reactions where phenanthroline has been used to mediate the reactions, the use of diketopiperazines can lead to either 1,1,2-triarylethenes or 1,1,2-triarylethanes, depending on the conditions used.

## 1. Introduction

**Figure 1:**
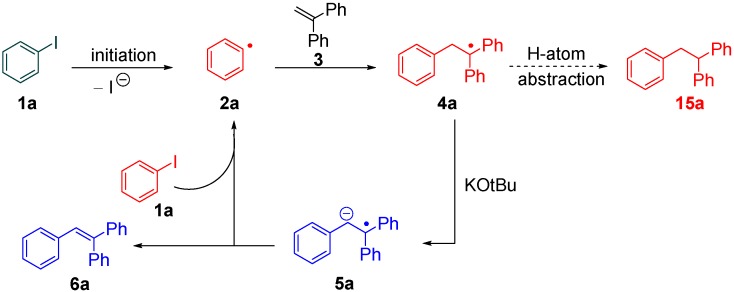
Mechanism of the coupling of iodoarenes to styrenes.

Stilbenes include a range of medicinally important molecules [1]. Traditionally, they can be formed by coupling haloarenes to styrenes, catalyzed by palladium salts or complexes [2,3,4,5]. However, in 2011, Shirakawa, Zhang and Hayashi [6] published a paper in which KO*t*Bu (3 equiv) and EtOH (0.2 equiv) brought about coupling reactions between iodobenzenes and styrenes in DMF at 80 °C, to afford the stilbene products in the absence of any palladium or other transition metal sources. Aryl iodides worked well and some aryl chlorides and bromides were also effectively coupled, but only in cases where the π-system extended beyond simple halobenzenes, e.g., halonaphthalenes. (this work followed closely on the heels of the KO*t*Bu-mediated coupling of halobenzenes to arenes [7,8,9,10]). This paper [6] demonstrated the intermediacy of benzylic radicals, such as **4a**, which resulted from addition of an aryl radical to the styrene. A full mechanism was proposed that has since been adapted [11,12] to the mechanism shown in Figure 1. The initiation of the reaction was proposed to occur by electron transfer to the iodoarene **1a**. Hayashi *et al*., suggested that the electron would be transferred from a butoxide anion, thereby forming a butoxyl radical, but see below. The resulting aryl radical **2a** adds to 1,1-diphenylethene **3** to afford radical **4a**. Deprotonation of this radical affords the stabilised radical anion **5a**. This then forms the stilbene product **6a** by electron transfer to another molecule of iodobenzene **1a** that enters the chain reaction.

**Figure 2:**
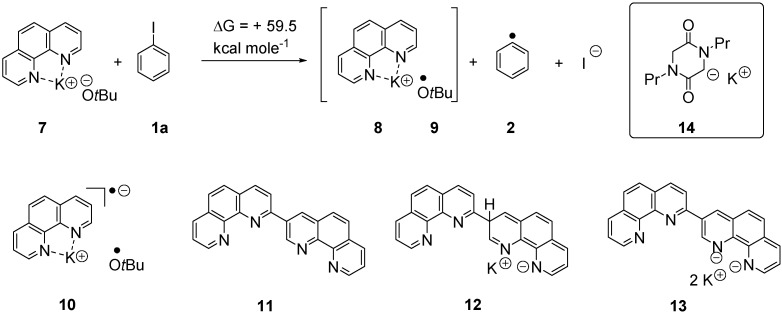
Species potentially involved in the electron-transfer initiation reactions.

Two other research groups reported closely related findings in the same year. Shi *et al*., observed [13] intermolecular and intramolecular couplings of iodoarenes to 1,1-diphenylethenes, mediated by KOtBu in benzene as solvent at 110 °C., but in the presence of phenanthrolines as additives. Taking phenanthroline itself in Figure 2 as an example, they proposed that the phenanthroline complex of KO*t*Bu **7** acted as an electron donor to the aryl halide to initiate the radical reactions. Rueping *et al*., likewise [14] used phenanthroline and KO*t*Bu to bring about intramolecular coupling of iodoaryl moieties with styryl and 1,1-diphenylethenyl groups. More recently, Rossi *et al*. [1] reported a new version of the Hayashi process (*i.e*., in the absence of organic additives), using photostimulation to enhance the initiation.

The issue of electron transfer when KO*t*Bu reacts in these reactions is of much current interest. For a phenanthroline complex, **7**, Tuttle, Murphy *et al*. [15] showed that electron transfer to iodobenzene would have a thermodynamic difference between starting materials and products ∆G = +59.5 kcal·mol^−1^, when calculated at the M06L/6-311G(d,p) level of theory using the CPCM continuum solvation method to incorporate the polarizing effect of the benzene solvent. It is stressed that this is not the kinetic barrier, which is likely to be considerably higher. The reaction is so endergonic as to make such an electron transfer untenable in ground state chemistry. Moreover they isolated a single distinctive product, **11**, resulting from *in situ* dimerisation of phenanthroline and quenching by an electron acceptor (in this case, iodine). This drove them to propose that the strong electron donors, anion **12** and/or dianion **13**, are produced during these reactions and may play a crucial role in the electron transfer steps.

Since then, Wilden *et al*., reported [16] evidence for decomposition products of *tert*-butoxyl radical in coupling reactions, and therefore supporting the role of electron transfer from *tert*-butoxide anion. In their case, they proposed that the electron transfer occurs to the phenanthroline to form the phenanthroline radical anion that then acts as the electron donor. Very recently, Lei, Jutand *et al*., proposed [17] that the phenanthroline radical anion, resulting from electron transfer from butoxide to phenanthroline, is the active electron donor although, surprisingly, they do not cite the Wilden paper [16] that came to the same conclusion. Both of these papers cite the Tuttle and Murphy [15] paper, but Wilden comments on our computational results and doesn’t discuss the experimental results including the isolation of **11** and its implications, while Lei and Jutand mention the paper only in the context that it reports that the “mechanism of action of 1,2-diamines” as organic additives in similar reactions is not yet clarified (that was not studied in the paper) but don’t discuss the core findings of the paper. A further paper has appeared from Yuan *et al.* [18] that uses a photoactivated complex of KOtBu with phenanthroline, but cites none of these papers.

In the absence of phenanthroline additives, (*i.e*., the conditions used by Hayashi), Murphy and Tuttle [15] proposed that traces of benzyne, formed from reaction of KO*t*Bu with iodoarenes, can initiate the reactions [1]. To date, phenanthrolines are the only organic additives to have triggered these reactions. In exploring the scope of the coupling reactions and in understanding the roles of organic additives, it would be helpful to explore alternative compounds that perform a similar role. That is the task of this paper.

## 2. Results and Discussion

Very recently, Tuttle and Murphy *et al.* reported [19] that the enolate **14** of *N*,*N'*-dialkyldiketopiperazine **16** could act as an electron donor and trigger coupling reactions between aryl halides and arenes. In coupling haloarenes to arenes, a number of authors had previously found that aminoacids, in the presence of KO*t*Bu, played a helpful role [9,20,21]. Moreover, it seemed that *N*-monoalkyl aminoacids (*i.e*., secondary aminoacids) were more helpful than primary aminoacids. We recently suggested that this might be explained if the secondary aminoacids underwent condensation reactions *in situ*, that could lead to *N,N'*-dialkyldiketopiperazines [19]. In turn, these could be deprotonated to form enolates that would act as electron donors. Regardless of whether that happens for secondary amino acids, our interest in the possible role of enolates such as **14** was heightened. This paper now investigates the coupling of iodoarenes with styrenes and 1,1-diphenylethenes in the presence of KO*t*Bu and *N*,*N'*-dialkyldiketopiperazines.

The prototype reaction used for the study was the coupling of iodobenzene (**1a)** to 1,1-diphenylethene **3** (Table 1). In the presence of **16** (0.5 equiv), and heating in benzene for 16 h at 100 °C (entry 1) afforded products **6a** (11%) and **15a** (18%). Product **15a** presumably arises from coupling of the aryl radical to the alkene; hydrogen abstraction by this radical then occurs more rapidly than deprotonation to form radical anion **5a**, the precursor of **6a**. Table 1 explores the optimisation of this reaction, in which various solvents (DMSO, DMF, benzene), different temperatures and different numbers of equivalents of butoxide, of the diketopiperazine and of the diphenylethene were used. The most successful conditions for forming coupling products are shown in entries 10 and 11. In entry 10, the number of equivalents of **16** is at a minimum (0.05), while the number of equivalents of KO*t*Bu (10) is at a maximum. The latter helps to maximize the formation of **6a**, since under these conditions, the deprotonation of the benzylic radical **4a** is most likely, thereby leading to the product **6a**, following electron transfer. On the other hand, conditions for entry 11 minimize the concentration of KO*t*Bu, but uses higher concentrations of the diketopiperazine **16** and this leads to **15** as the predominant product. In this Table, Entry 8 shows the importance of using KO*t*Bu, as potassium carbonate is not effective in affording coupling. Entry 12 shows that the diketopiperazine **16** is required to afford the products; in its absence, only a trace of product formed. This is in keeping with the proposed initiator role of enolate **14** as an electron donor.

**Table 1:** Optimisation conditions for the coupling of iodobenzene **1a** to 1,1-diphenylethene **3**. 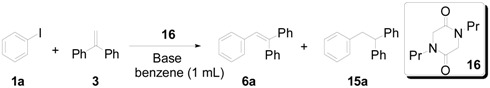

Entry	3 (Equiv.)	16 (Equiv.)	Base (Equiv.)	Solvent	T (°C)	Reaction Time (h)	6a (%) ^a^	15a (%) ^a^
1	1	0.5	KOtBu (3)	benzene	100	16	11	18
2	1	0.1	KOtBu (5)	benzene	110	16	22	13
3	1	0.1	KOtBu (5)	benzene	160	16	25	8
4	1	0.1	KOtBu (5)	DMSO	160	16	-- ^b^	-- ^b^
5	3	0.1	KOtBu (5)	DMF	160	16	6	13
6	3	0.3	KOtBu (3)	benzene	110	36	25	43
7	5	0.1	KOtBu (6)	benzene	110	36	36	26
8	5	0.1	K_2_CO_3_ (6)	benzene	110	36	0	0
9	5	0.2	KOtBu (6)	benzene	110	36	25	42
**10**	**5**	**0.05**	**KOtBu (10)**	**benzene**	**110**	**36**	**58**	**14**
**11**	**5**	**0.3**	**KOtBu (1.5)**	**benzene**	**110**	**36**	**20**	**63**
12	5	-	KOtBu (6)	benzene	110	36	trace	trace

Notes: Reactions were carried out using 0.5 mmol of **1a** in a sealed pressure tube and provided 1%–5% of biphenyl product (except for Entries 5 and 8) along with **6a** and **15a**. ^a^ Isolated product yields. ^b^ Starting material was decomposed.

**Figure 3:**
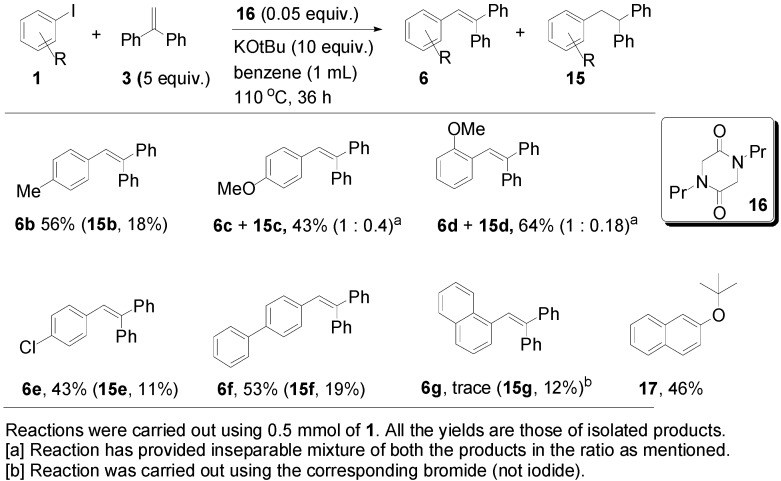
Scope of the formation of alkene products **6**.

These optimised conditions of entry 10 were then applied to the formation of coupled alkene products **6**. Figure 3 shows the outcomes. With the exception of the naphthalene cases arising from 1-bromonaphthalene, in each case, a mixture of two products, the triarylethene **6** and triarylethane **15**, was formed. Where separation of the two products was possible, their isolated yields are quoted as the percentages above. In some cases, separation of the two products was not possible and, there, an overall yield is followed by a ratio of the two products. In each case, the alkene predominates, with the ratios varying from 2.5:1 to more than 5.5:1. In the case of 1-bromonaphthalene, it appears that very easy elimination to the corresponding benzyne occurred. Addition of *tert*-butoxide occurred selectively at the 2-position, ultimately affording 2-*t-*butoxynaphthalene **17** [22]. Even allowing for that, 1-naphthyl radicals were clearly also formed as shown by the formation of **6g** and **15g**. It is not clear why the alkene product, **6g**, was not isolated in greater yield in this case.

**Figure 4:**
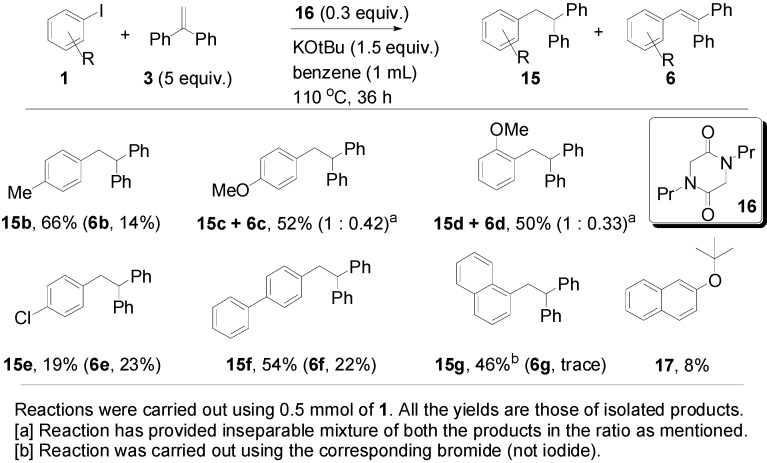
Scope of the formation of triarylethane products **15**.

Returning to Table 1, entry 11 showed the optimised conditions for the formation of the 1,1,2-triarylethanes **15**. Here, far less KO*t*Bu (1.5 equiv) was used, and the amount of diketopiperazine **16** was higher (0.3 equiv) than for entry 10. Figure 4 reports our studies on optimization of the yields of **15**. It should be noted that while the formation of the alkene product **6** leads to continuation of a radical chain shown in Figure 1, the formation of **15** does not.

At the end of the addition sequence, hydrogen abstraction is required, and the most likely source of that hydrogen is the molecule **16**, since abstraction from one of its CH_2_ groups would afford a stabilized captodative radical. However, there is a deficiency of this molecule (only 0.3 equiv), and since more than 60% of product **15** is formed this suggests that each molecule of **16** provides more than one hydrogen atom. This will be discussed further below (Figure 7). With 1-bromonaphthalene, 1,1,2-triarylethane product **15g** (46%) was formed in preference to 1,1,2-triarylethene **6g** (trace amounts), but 2-*t-*butoxynaphthalene **17** was again isolated (8%).

**Figure 5:**
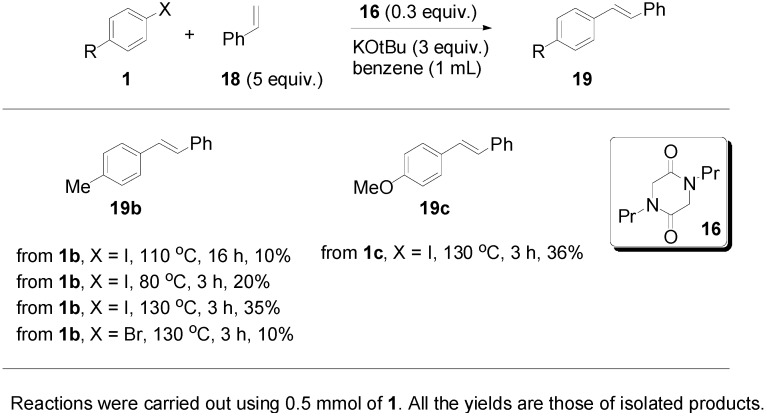
Formation of stilbene products from styrenes.

Proceeding to test the reaction in coupling with styrenes (Figure 5), it is clear that the reaction affords yields that are lower than for 1,1-diphenylethenes. Iodides work better than bromides, and the higher temperature of 130 °C works better than lower temperatures.

**Figure 6:**
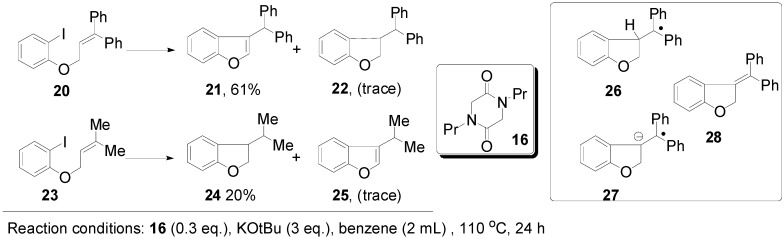
The importance of acidity in forming alkene products.

In reaching the unsaturated products, the importance of the acidity of the proton to be removed in transforming benzylic radical **4** to the corresponding radical anion **5** (Figure 1) is key. This is seen in comparing the cyclisations of substrates **20** and **23** (Figure 6). Cyclisation of the aryl radical derived from iodoarene **20** onto the diphenylethene affords benzylic radical **26**. Deprotonation of this radical gives a radical anion **27** that can delocalize over the three aromatic rings. Hence the diphenylethene **28** can form in good yield, allowing the chain reaction to continue. Either during the reaction or on work up, isomerisation of the stilbene central double-bond in **28** occurs to afford the benzofuran **21**. In contrast, deprotonation of the radical resulting from cyclisation of the aryl radical arising from **23** does not lead to such a delocalized radical anion, and so the deprotonation is relatively inhibited, with hydrogen atom abstraction occurring instead to form **24**.

**Figure 7:**
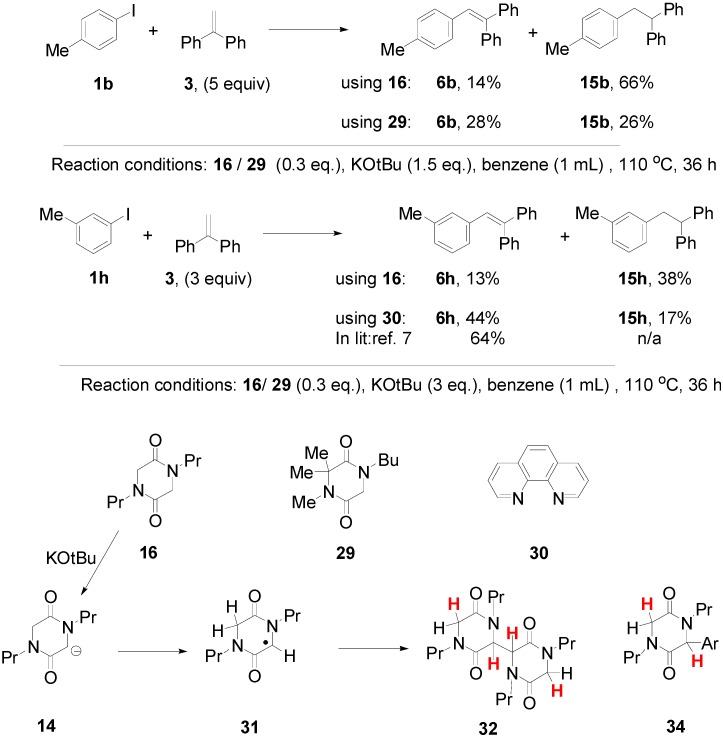
The importance of hydrogen atom abstraction.

The importance of the hydrogen abstraction step is revisited in Figure 7. Here two diketopiperazines **16** and **29** were compared, mindful of what was said above about hydrogen abstraction. The two additives gave very different results. Compound **16** afforded significantly higher yield (66%) of the reduced compound **15b**, indicating that the additional CH_2_ group in **16** plays a major role in determination of the pathways to product. Since 0.3 equiv of the *N,N'*-dialkyl diketopiperazine **16** was used, and more than 60% of dihydrostilbene product **15b** formed, it suggests that on average the *N,N'*-dialkyl diketopiperazine, as the likely source for hydrogen atom abstraction, can be responsible for providing two hydrogen atoms.

If the diketopiperazine requires to be deprotonated to **14**, and later transforms into radical **31** following electron transfer then, in either case, only a single hydrogen from the remaining methylene group would be available for hydrogen atom abstraction. However, if dimerization of radical **31** to **32** should occur [23] (**32** could also arise by combination of radical **31** with anion **14**, followed by electron transfer) or if combination of **31** with another radical (e.g., an aryl radical) to form **34** should occur, then additional hydrogens come into play (shown in red in Figure 7), that can account for the observed yields of products.

The results from the *m*-tolyl case **1h** were less pronounced, but comparison with the experiment using phenanthroline **30** shows that the yield of saturated product **15h** is enhanced when the diketopiperazine is used. The literature [13] reports a 64% yield of the stilbene, whereas our experiments afford a 61% yield, (but as a 44% + 17% mixture of the stilbene and the dihydrostilbene product).

## 3. Experimental Section

### 3.1. General Information

All the reactions were performed in oven-dried or flame-dried apparatus and preparation of the substrates was carried out under argon atmosphere using dry solvents. Diethyl ether, tetrahydrofuran, dichloromethane and hexane were dried with a Pure-Solv 400 solvent purification system by Innovative Technology Inc., Amesbury, MA, USA. A glove box (Innovative Technology Inc., Amesbury, MA, USA) was used to weigh out the super-electron-donor (SED) into the reaction flask. All the reagents were bought from commercial suppliers and used without further purification unless stated otherwise. A Büchi rotary evaporator was used to concentrate the reaction mixtures. Thin layer chromatography (TLC) was performed using aluminum-backed sheets of silica gel and visualized under a UV lamp (254 nm). The plates were developed using vanillin or KMnO_4_ solution. Column chromatography was performed to purify compounds by using silica gel 60 (200–400 mesh).

Proton (^1^H) NMR spectra were recorded at 400 MHz on a Bruker DPX 400 spectrometer or at 500 MHz on a Bruker DRX 500 spectrometer. Carbon NMR (^13^C) spectra were recorded at 100 MHz or 125 MHz respectively. The chemical shifts are quoted in parts per million (ppm) by taking tetramethylsilane as a reference (δ = 0) but calibrated on the residual non-deuterated solvent signal. Signal multiplicities are abbreviated as: s, singlet; d, doublet; t, triplet; q, quartet; m, multiplet; bs, broad singlet; coupling constants are given in Hertz (Hz).

Infra-Red spectra were recorded on a Perkin Elmer Spectrum One FT IR Spectrometer either pressed as discs in KBr or as films applied on NaCl crystal plates or using Shimadzu FT-IR Spectrophotometer (Model IRAffinity-1) with a MIRacle Single Reflection Horizontal ATR Accessory. Melting points were determined on a Gallenkamp Melting point apparatus. High resolution mass spectra were recorded at the EPSRC National Mass Spectrometry Service Centre, Swansea. The spectra were recorded using electron ionization (EI), chemical ionization (CI), fast atom bombardment (FAB) or electrospray ionization (ESI) techniques as stated for each compound.

### 3.2. Preparation of Piperazinediones

#### 3.2.1. Synthesis of Compound **16**


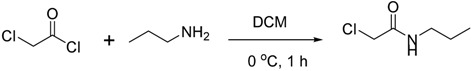


Chloroacetyl chloride (5.60 g, 50 mmol) was added dropwise to a flask containing a solution of *n-*propylamine (5.9 g, 100 mmol) in DCM (50 mL) cooled in an ice-water bath. The resulting reaction mixture was stirred in the cooling ice bath for 1 h, diluted with ether (100 mL), and filtered. The filtrate was then concentrated. The residue was again dissolved in ether (100 mL) and the solid was filtered. Concentration of the filtrate provided 2-chloro-*N*-propylacetamide (6.73 g, 100%) as a pure pale yellow oil. The product was used without further purification [19].


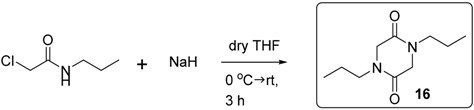


To a flask containing a suspension of NaH (1.80 g, 45 mmol) in dry THF (10 mL) was added dropwise a solution of 2-chloro-*N*-propylacetamide (6.076 g, 45 mmol) in dry THF (30 mL) at 0 °C, under argon gas flow. After the completion of the addition, the resulting reaction mixture was stirred at room temperature for 3 h. Then the mixture was diluted with ether (70 mL) and filtered. The filtrate was concentrated and purified by column chromatography (hexane/EtOAc = 50/50 → 100% EtOAc). 1,4-Di-*n-*propylpiperazine-2,5-dione **16** [19] (723 mg, 73%) was obtained as a white solid. mp, 40 °C–42 °C; ν*_max_* (neat)/cm^−1^ 2965, 2934, 2874, 1653, 1482, 1336, 1310, 1279, 1206, 1057; [Found: (FTMS+) (M+H)^+^ 199.1439, C_10_H_19_N_2_O_2_ (M+H) requires 199.1441]; ^1^H-NMR (400 MHz, CDCl_3_): δ 0.93 (6H, t, *J* = 7.2 Hz, NCH_2_CH_2_C*H*_3_), 1.55–1.63 (4H, m, NCH_2_C*H*_2_CH_3_), 3.35–3.39 (4H, m, NC*H*_2_CH_2_CH_3_), 3.96 (4H, s, COC*H*_2_N); ^13^C-NMR (100 MHz, CDCl_3_): δ 11.2 (CH_3_), 20.1 (CH_2_), 47.7 (CH_2_), 50.1 (CH_2_), 163.6 (C).

#### 3.2.2. Synthesis of Compound **29**


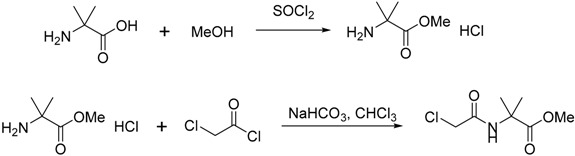


To a suspension of 2-amino-2-methylpropanoic acid (5.25 g, 50.97 mmol) in MeOH (60 mL), cooled in an ice-water bath, was added thionyl chloride (3.9 mL, 53.7 mmol) dropwise over about 20 min. The resulting solution was heated to 55–60 °C for 4 h, then cooled to room temperature, and concentrated. More MeOH (50 mL) was added, and concentrated under vacuum. The white solid obtained was used in the next reaction without purification.

Crude methyl 2-amino-2-methylpropanoate hydrogen chloride salt was dissolved in water. NaHCO_3_ (12.9 g, 153 mmol) was added, followed by chloroform (30 mL). The 2-chloroacetyl chloride (4.4 mL, 55 mmol) in chloroform (5 mL) was added dropwise. The mixture was stirred at room temperature overnight. The organic layer was separated. The aqueous layer was extracted with chloroform (30 mL). The combined organic layer was dried over Na_2_SO_4_, filtered and concentrated. Column purification (EtOAc) of the crude product provided methyl 2-(2-chloroacetamido)-2-methyl propanoate, (5.47 g, 56% over two steps) as a white solid that was used in the next step [19].


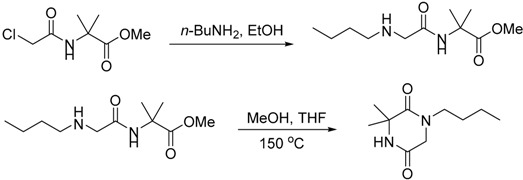


A solution of 2-(2-chloroacetamido)-2-methyl propanoate (850 mg, 4.41 mmol) and *n*-BuNH_2_ in ethanol (15 mL) was heated at 55 °C for 3 h, and the mixture was cooled to room temperature. The volatiles were removed on a rotary evaporator. The residue was dissolved in EtOAc and washed with aqueous potassium carbonate solution (5 mL, containing K_2_CO_3_ (1.0 g). The organic layer was separated and concentrated. The residue was dissolved in MeOH (5 mL) and THF (5 mL), sealed in a pressure tube and heated to 150 °C overnight behind a shield. The mixture was cooled to room temperature. The solvent was removed under vacuum, and the residue was purified by column chromatography (EtOAc). 1-Butyl-3,3-dimethylpiperazine-2,5-dione [19] was obtained as a white solid, (645 mg, 74% over two steps). mp 78–80 °C; ν*_max_* (neat)/cm^−1^ 3073, 2963, 2930, 2872, 1682, 1645, 1450, 1427, 1298, 1196, 825, 785; [Found: (FTMS) (M+H)^+^ 199.1438, C_10_H_19_N_2_O_2_ (M+H) requires 199.1441]; ^1^H-NMR (400 MHz, CDCl_3_): δ 0.94 (3H, t, *J* = 7.3 Hz), 1.29–1.38 (2H, m), 1.49 (6H, s), 1.50–1.58 (2H, m), 3.4 (2H, t, *J* = 7.4 Hz), 3.96 (2H, s), 7.55 (1H, broad); ^13^C-NMR (100 MHz, CDCl_3_): δ 13.2, 19.4, 27.3, 28.2, 45.8, 49.4, 55.5, 165.7, 168.6.


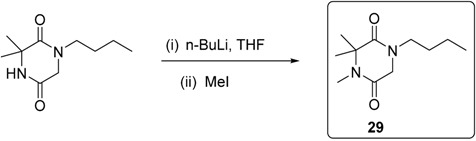


To a solution of 1-butyl-3,3-dimethylpiperazine-2,5-dione (220 mg, 1.11 mmol) in THF (8 mL) cooled in an ice-water bath under argon was added *n*-BuLi (0.5 mL, 1.25 mmol) dropwise. The mixture was stirred at room temperature for 10 min, then it was cooled in ice-water bath again. Iodomethane (0.2 mL) was added and the mixture was stirred at room temperature overnight. The reaction was quenched by water (30 mL). The mixture was extracted with EtOAc (2 × 30 mL). The organic layer was dried over Na_2_SO_4_, filtered and concentrated. Purification by column chromatography (EtOAc) gave 1-butyl-3,3,4-trimethylpiperazine-2,5-dione **29** [19] as a yellow oil (118 mg, 50%). ν*_max_* (neat)/cm^−1^ 2959, 2932, 2875, 1651, 1456, 1429, 1400, 1379, 1308, 1211, 1153, 989, 752; [Found: (FTMS) (M+H)^+^ 213.1594, C_11_H_21_N_2_O_2_ (M+H) requires 213.1598]; ^1^H-NMR (400 MHz, CDCl_3_): δ 0.85 (3H, t, *J* = 7.3 Hz), 1.25–1.29 (2H, m), 1.39–1.50 (2H, m), 1.43 (6H, s), 2.89 (3H, s), 3.13 (2H, t, *J* = 7.4 Hz), 3.89 (2H, s); ^13^C-NMR (100 MHz, CDCl_3_): δ 13.2, 19.3, 24.2, 27.2, 28.0, 45.7, 48.9, 60.1, 163.6, 168.0.

### 3.3. Synthesis of (3-(2-Iodophenoxy)prop-1-ene-1,1-diyl)dibenzene (**20**)


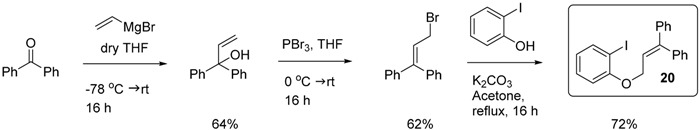


To a flask equipped with condenser, magnesium turnings (0.583 g, 24 mmol), iodine (50 mg), and dry THF (20 mL) were added and the flask was kept under argon gas flow. Vinyl bromide (1 M in THF, 24 mL, 24 mmol) was added drop-wise into the flask while heating the reaction mixture at 40 °C. Later, the resulting reaction mixture was stirred for 1 h at 50 °C before cooling it to room temperature. This solution was then added dropwise using a cannula into another flask containing benzophenone (3.644 g, 20 mmol) in THF (20 mL) at −78 °C. The resulting reaction mixture was then warmed to room temperature and stirred for 16 h. Reaction contents were then quenched with ice and extracted with diethyl ether (3 × 30 mL). The combined ether layers were washed with water (15 mL), brine (15 mL), dried over anhydrous Na_2_SO_4_, filtered and concentrated using rotary evaporator. Purification of the crude product by column chromatography (15% EtOAc in hexane) afforded 1,1-diphenylprop-2-en-1-ol [13] (2.691 g, 64%).

A solution of phosphorus tribromide (2.7016 g, 10.0 mmol) was added drop-wise to a flask containing 1,1-diphenylprop-2-en-1-ol (1.892 g, 9 mmol) and pyridine (0.711 g, 9 mmol) in dry tetrahydrofuran (20 mL) at 0 °C under argon gas. The resulting solution was warmed to room temperature and further stirred for 16 h. At this point, the reaction was quenched with water (10 mL) and extracted with diethyl ether (3 × 10 mL). The combined ether layers were washed with water (10 mL), brine (10 mL), dried over anhy. Na_2_SO_4_, filtered, concentrated using rotary evaporator and afforded (3-bromoprop-1-ene-1,1-diyl)dibenzene [13] (1.516 g, 62%).

To a flask containing K_2_CO_3_ (0.691 g, 5 mmol) in acetone (20 mL) was added with 2-iodophenol (0.660 g, 3 mmol) in acetone (2 mL) and (3-bromoprop-1-ene-1,1-diyl)dibenzene (0.682 g, 2.5 mmol) in acetone (2 mL) at room temperature and the resulting reaction mixture was stirred at reflux conditions for 16 h. At this point, the reaction was quenched with water (10 mL) and extracted with diethyl ether (3 × 15 mL). The combined ether layers were washed with water (10 mL), brine (10 mL), dried over anhydrous Na_2_SO_4_, filtered, concentrated using rotary evaporator. The crude product was then purified by column chromatography (2% diethyl ether in hexane) to afford desired (3-(2-iodophenoxy)prop-1-ene-1,1-diyl)dibenzene **19** [13] (0.746 g, 72%) as a viscous pale yellow oil. ν*_max_* (neat)/cm^−1^ 3057, 3023, 1580, 1470, 1441, 1377, 1241, 1020, 731; ^1^H-NMR (400 MHz, CDCl_3_) δ 4.67 (2H, d, *J* = 6.8 Hz, ArOC*H*_2_), 6.38 (1H, t, *J* = 6.8 Hz, *H*C = CPh_2_), 6.66–6.72 (2H, m, Ar*H*), 7.20–7.31 (8H, m, Ar*H*), 7.36–7.44 (3H, m, Ar*H*), 7.79 (2H, dd, *J* = 7.6, 1.6 Hz, ArH); ^13^C-NMR (100 MHz, CDCl_3_) δ 67.5 (CH_2_), 87.1 (C), 113.0 (CH), 122.8 (CH), 123.6 (CH), 127.9 (CH), 128.0 (CH), 128.3 (CH), 128.5 (CH), 129.4 (CH), 129.9 (CH), 139.0 (C), 139.6 (CH), 141.6 (C), 146.0 (C), 157.4 (C). Spectral data of this compound were consistent with literature data [13].

### 3.4. Synthesis of 1-Iodo-2-((3-methylbut-2-en-1-yl)oxy)benzene (**23**)


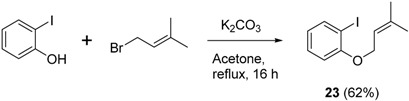


To a flask containing K_2_CO_3_ (0.829 g, 6 mmol) in acetone (20 mL) was added with 2-iodophenol (0.968 g, 4.4 mmol) in acetone (2 mL) and 1-bromo-3-methylbut-2-ene (0.596 g, 4 mmol) in acetone (2 mL) at room temperature and the resulting reaction mixture was stirred at reflux conditions for 16 h. At this point, the reaction was quenched with water (10 mL) and extracted with diethyl ether (3 × 15 mL). The combined ether layers were washed with water (10 mL), brine (10 mL), dried over anhy. Na_2_SO_4_, filtered, concentrated using rotary evaporator. The crude product was then purified by column chromatography (2% diethyl ether in hexane) to afford desired 1-iodo-2-((3-methylbut-2-en-1-yl)oxy)benzene (0.719 g, 62%) as a colourless oil. ν*_max_* (neat)/cm^−1^ 3060, 2913, 2930, 1582, 1470, 1439, 1383, 1275, 1230, 1050, 1018, 746; ^1^H-NMR (400 MHz, CDCl_3_) δ 1.76 (3H, s, C*H*_3_), 1.80 (3H, s, C*H*_3_), 4.59 (2H, d, *J* = 6.8 Hz, ArOC*H*_2_), 5.50–5.54 (1H, m, *H*C = C(CH_3_)_2_), 6.71 (1H, td, *J* = 7.6, 1.6 Hz, Ar*H*), 6.83 (1H, d, *J* = 8.0, 1.2 Hz, Ar*H*), 7.26–7.31 (1H, m, Ar*H*), 7.78 (1H, dd, *J* = 8.0, 1.6 Hz, ArH); ^13^C-NMR (100 MHz, CDCl_3_) δ 18.5 (CH_3_), 25.9 (CH_3_), 66.4 (CH_2_), 87.1 (C), 112.8 (CH), 119.6 (CH), 122.5 (CH), 129.4 (CH), 138.0 (C), 139.6 (CH), 157.6 (C). Spectral data of this compound were consistent with literature data [24].

### 3.5. General Reaction Procedure

Substrate (0.5 mmol) and additive were added to a pressure tube. KO*^t^*Bu and benzene were added into the tube in a glove box. The tube was then sealed properly before removing from the glove box and reaction was done as described in the Tables (time and temperature). Later, the pressure tube was cooled to room temperature. The reaction was quenched with water (10 mL) and extracted with diethyl ether (3 × 15 mL). The combined ether layers were then washed with water (10 mL), brine (10 mL) and dried over anhydrous sodium sulfate. The crude product was obtained after evaporation of solvent under reduced pressure using a rotary evaporator. The crude product was then adsorbed onto silica and purified by column chromatography (100% hexane), providing the corresponding coupling products.

#### 3.5.1. Data for Compound **6a**


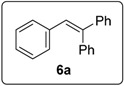


White solid m.p. 70–72 °C (lit.: [25] 70 °C). ν*_max_* (neat)/cm^−1^ 3057, 3023, 1601, 1493, 1446, 1078, 1033, 908, 731; ^1^H-NMR (400 MHz, CDCl_3_) δ 6.98 (1H, s, Ar*CH* = CPh_2_), 7.03–7.05 (2H, m, ArH), 7.11–7.15 (3H, m, ArH), 7.21–7.23 (2H, m, ArH), 7.29–7.36 (8H, m, ArH); ^13^C-NMR (100 MHz, CDCl_3_) δ 126.9 (CH), 127.5 (CH), 127.6 (CH), 127.7 (CH), 128.1 (CH), 128.3 (CH), 128.34 (CH), 128.8 (CH), 129.7 (CH), 130.5 (CH), 137.5 (C), 140.5 (C), 142.7 (C), 143.6 (C). Spectral data of this compound were consistent with literature data [26].

#### 3.5.2. Data for Compound **15a**


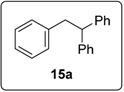


White solid m.p. 50–52 °C (lit.: [20] 50–52 °C). ν*_max_* (neat)/cm^−1^ 3062, 3027, 2935, 1601, 1495, 1448, 1070, 1031, 694; ^1^H-NMR (400 MHz, CDCl_3_) δ 3.38 (2H, d, *J* = 7.6 Hz, ArC*H*_2_CH), 4.24 (1H, t, *J* = 7.6 Hz, ArCH_2_C*H*), 7.00–7.02 (2H, m, ArH), 7.11–7.28 (3H, m, ArH); ^13^C-NMR (100 MHz, CDCl_3_) δ 42.2 (CH_2_), 53.2 (CH), 126.0 (CH), 126.3 (CH), 128.2 (CH), 128.5 (CH), 129.2 (CH), 140.4 (C), 144.6 (2 × C). Spectral data of this compound were consistent with literature data [27].

#### 3.5.3. Data for Compound **6b**


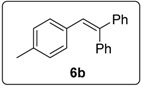


White solid m.p. 70–72 °C (lit.: [26] 69–70 °C). ν*_max_* (neat)/cm^−1^ 3079, 3055, 3023, 1599, 1495, 1446, 1031, 908, 698; ^1^H-NMR (400 MHz, CDCl_3_) δ 2.28 (3H, s, C*H*_3_), 6.91–6.97 (5H, m, ArH + =CH), 7.21–7.24 (2H, m, ArH), 7.27–7.35 (8H, m, ArH); ^13^C-NMR (100 MHz, CDCl_3_) δ 21.3 (CH_3_), 127.4 (CH), 127.5 (CH), 127.6 (CH), 128.2 (CH), 128.3 (CH), 128.8 (CH), 128.9 (CH), 129.6 (CH), 130.5 (CH), 134.7 (C), 136.7 (C), 140.7 (C), 141.8 (C), 143.7 (C). Spectral data of this compound were consistent with literature data [26].

#### 3.5.4. Data for Compound **15b**


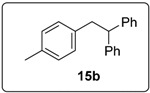


White solid m.p. 78–80 °C (lit.: [28] 80 °C). ν*_max_* (neat)/cm^−1^ 3084, 3025, 2917, 1601, 1493, 1452, 1035, 1022, 700; ^1^H-NMR (400 MHz, CDCl_3_) δ 2.28 (3H, s, CH_3_), 3.34 (2H, d, *J* = 7.6 Hz, ArC*H*_2_CH), 4.23 (1H, t, *J* = 7.6 Hz, ArCH_2_C*H*), 6.91 (2H, d, *J* = 7.2 Hz, ArH), 6.99 (2H, d, *J* = 8.0 Hz, ArH), 7.15–7.19 (2H, m, ArH), 7.22–7.28 (8H, m, ArH); ^13^C-NMR (100 MHz, CDCl_3_) δ 21.1 (CH_3_), 41.8 (CH_2_), 53.3 (CH), 126.3 (CH), 128.2 (CH), 128.5 (CH), 128.9 (CH), 129.0 (CH), 135.4 (C), 137.3 (C), 144.6 (2 × C). Spectral data of this compound were consistent with literature data [28].

#### 3.5.5. Data for Compound **6e**


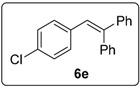


Colourless viscous oil. ν*_max_* (neat)/cm^−1^ 3057, 3023, 1493, 1444, 1053, 1037, 908, 731; [Found: (CI^+^ corona) (M+H)^+^ 291.0937 C_20_H_16_Cl (M+H) requires 291.0935]; ^1^H-NMR (400 MHz, CDCl_3_) δ 6.82–6.84 (1H, m, ArH), 6.87–6.91 (1H, m, ArH), 7.04–7.08 (1H, m, ArH), 7.10 (1H, s, ArC*H*=CPh_2_), 7.14–7.16 (2H, m, ArH), 7.26–7.28 (3H, m, ArH), 7.32–7.38 (6H, m, ArH); ^13^C-NMR (100 MHz, CDCl_3_) δ 125.2 (CH), 126.1 (CH), 127.6 (CH), 128.0 (CH), 128.2 (CH), 128.4 (CH), 128.5 (CH), 129.4 (CH), 130.8 (CH), 131.3 (CH), 134.7 (C), 136.4 (C), 139.9 (C), 143.1 (C), 144.8 (C); *m/z* (CI^+^ corona) 291 [(M+H)^+^, 34%], 197 (22), 181 (100), 111 (14). Spectral data of this compound were consistent with literature data [13].

#### 3.5.6. Data for Compound **15e**


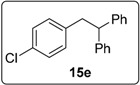


White solid m.p. 54–56 °C. ν*_max_* (neat)/cm^−1^ 3062, 3027, 1603, 1496, 1450, 1053, 1040, 754; [Found: (CI^+^ corona) (M+NH_4_)^+^ 310.1351. C_20_H_21_NCl (M+NH_4_) requires 310.1357]; ^1^H-NMR (400 MHz, CDCl_3_) δ 3.49 (2H, d, *J* = 7.6 Hz, ArC*H*_2_), 4.40 (1H, t, *J* = 7.6 Hz, ArCH_2_C*H*), 6.81 (1H, dd, *J* = 7.6, 1.6 Hz, ArH), 6.97 (1H, td, *J* = 7.6, 1.6 Hz, ArH), 7.08 (1H, td, *J* = 7.6, 1.6 Hz, ArH), 7.16–7.29 (10H, m, ArH), 7.33 (1H, dd, *J* = 8.0, 1.2 Hz, ArH) ^13^C-NMR (100 MHz, CDCl_3_) δ 39.9 (CH), 50.8 (CH_2_), 126.4 (CH), 126.42 (CH), 127.6 (CH), 128.3 (CH), 128.5 (CH), 129.5 (CH), 131.5 (CH), 134.4 (C), 137.8 (C), 144.2 (2 × C); *m/z* (CI^+^ corona) 310 [(M+NH_4_)^+^, 9%], 215 (18), 167 (100), 149 (20).

#### 3.5.7. Data for Compound **6f**


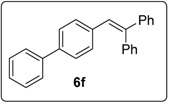


White solid m.p. 110–112 °C (lit.: [29] 113–114 °C). ν*_max_* (neat)/cm^−1^ 3053, 3023, 1601, 1493, 1444, 1078, 735; ^1^H-NMR (500 MHz, CDCl_3_) δ 7.03 (1H, s, ArC*H*=CPh_2_), 7.12 (2H, d, *J* = 8.5 Hz, ArH), 7.26–7.43 (15H, m, ArH), 7.57 (2H, d, *J* = 8.0 Hz, ArH); ^13^C-NMR (100 MHz, CDCl_3_) δ 126.7 (CH), 127.0 (CH), 127.4 (CH), 127.6 (CH), 127.7 (CH), 127.74 (CH), 127.8 (CH), 128.4 (CH), 128.86 (CH), 128.88 (CH), 130.1 (CH), 130.5 (CH), 136.6 (C), 139.5 (C), 140.6 (C), 140.7 (C), 142.8 (C), 143.6 (C). Spectral data of this compound were consistent with literature data [30].

#### 3.5.8. Data for Compound **15f**


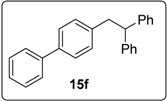


White solid m.p. 128–130 °C. ν*_max_* (neat)/cm^−1^ 3084, 3062, 3029, 1601, 1491, 1452, 1081, 1011, 765; [Found: (CI^+^ corona) (M+NH_4_)^+^ 352.2056. C_26_H_26_N (M+NH_4_) requires 352.2060]; ^1^H-NMR (400 MHz, CDCl_3_) δ 3.42 (2H, d, *J* = 7.6 Hz, ArC*H*_2_CH), 4.29 (1H, t, *J* = 7.6 Hz, ArCH_2_C*H*), 7.09 (2H, d, *J* = 8.0 Hz, ArH), 7.16–7.20 (2H, m, ArH), 7.24–7.34 (9H, m, ArH), 7.39–7.43 (4H, m, ArH), 7.55–7.57 (2H, m, ArH); ^13^C-NMR (100 MHz, CDCl_3_) δ 41.9 (CH_2_), 53.1 (CH), 126.4 (CH), 126.9 (CH), 127.0 (CH), 127.1 (CH), 128.2 (CH), 128.5 (CH), 128.8 (CH), 129.6 (CH), 138.8 (C), 139.5 (C), 141.1 (C), 144.6 (2 × C); *m/z* (CI^+^ corona) 352 [(M+NH_4_)^+^, 22%], 333 [(M−H)^+^, 38%], 249 (11), 195 (14), 171 (100), 149 (10).

#### 3.5.9. Data for Compound **17**


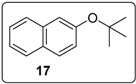


Colourless oil. ν*_max_* (neat)/cm^−1^ 3057, 2976, 2932, 1632, 1597, 1508, 1467, 1366, 1251, 1154, 1122, 968, 867, 752; ^1^H-NMR (400 MHz, CDCl_3_) δ 1.43 (9H, s, CC*H*_3_), 7.20 (1H, dd, *J* = 8.8, 2.4 Hz, ArH), 7.37–7.47 (3H, m, ArH), 7.66 (2H, d, *J* = 8.4 Hz, ArH), 7.81 (1H, d, *J* = 8.4 Hz, ArH); ^13^C-NMR (100 MHz, CDCl_3_) δ 29.1 (CH_3_), 79.1 (C), 119.9 (CH), 124.6 (CH), 125.1 (CH), 126.1 (CH), 127.3 (CH), 127.7 (CH), 128.7 (CH), 130.4 (C), 134.2 (C), 153.3 (C). Spectral data of this compound were consistent with literature data [31].

#### 3.5.10. Data for Compound **15g**


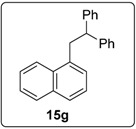


White solid m.p. 90–92 °C. ν*_max_* (neat)/cm^−1^ 3062, 3021, 2963, 1597, 1493, 1450, 1398, 1264, 1033, 1018, 782; [Found: (CI^+^ corona) (M+NH_4_)^+^ 326.1895. C_24_H_24_N (M+NH_4_) requires 326.1903]; ^1^H-NMR (400 MHz, CDCl_3_) δ 3.84 (2H, d, *J* = 7.6 Hz, ArC*H*_2_CH), 4.48 (1H, t, *J* = 7.6 Hz, ArCH_2_C*H*), 6.92 (1H, d, *J* = 6.4 Hz, ArH), 7.18–7.30 (11H, m, ArH), 7.49–7.54 (2H, m, ArH), 7.69 (1H, d, *J* = 8.4 Hz, ArH), 7.87–7.89 (1H, m, ArH), 8.06–8.09 (1H, m, ArH); ^13^C-NMR (100 MHz, CDCl_3_) δ 39.4 (CH_2_), 51.8 (CH), 123.8 (CH), 125.3 (CH), 125.5 (CH), 126.0 (CH), 126.4 (CH), 126.9 (CH), 127.4 (CH), 128.2 (CH), 128.5 (CH), 129.0 (CH), 132.1 (C), 134.0 (C), 136.0 (C), 144.8 (2 × C); *m/z* (CI^+^ corona) 326 [(M+NH_4_)^+^, 22%], 307 [(M−H)^+^, 42%], 229 (100), 167 (50), 153 (25).

#### 3.5.11. Data for Compound **18b**


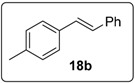


White solid m.p. 116–118 °C (lit.: [25] 118–119 °C). ν*_max_* (neat)/cm^−1^ 3051, 3027, 2921, 1599, 1513, 1450, 966, 908, 731; ^1^H-NMR (400 MHz, CDCl_3_) δ 2.38 (3H, s, ArC*H*_3_), 7.07 (1H, d, *J* = 16.4 Hz, ArC*H*=CHPh), 7.12 (1H, d, *J* = 16.4 Hz, ArCH=C*H*Ph), 7.18 (2H, d, *J* = 8.0 Hz, ArH), 7.24–7.28 (1H, m, ArH), 7.37 (2H, t, *J* = 7.6 Hz, ArH), 7.43 (2H, d, *J* = 8.4 Hz, ArH), 7.52 (2H, d, *J* = 7.2 Hz, ArH); ^13^C-NMR (100 MHz, CDCl_3_) δ 21.4 (CH_3_), 126.5 (CH), 126.6 (CH), 127.5 (CH), 127.9 (CH), 128.8 (CH), 129.5 (CH), 134.7 (C), 137.67 (C), 137.7 (C). Spectral data of this compound were consistent with literature data [32].

#### 3.5.12. Data for Compound **18c**


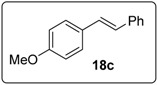


White solid m.p. 134–136 °C (lit.: [26] 136–137 °C). ν*_max_* (neat)/cm^−1^ 3066, 3023, 2965, 2839, 1603, 1595, 1513, 1448, 1249, 1180, 1031, 815; ^1^H-NMR (400 MHz, CDCl_3_) δ 3.84 (3H, s, ArC*H*_3_), 6.91 (2H, d, *J* = 8.8 Hz, Ar*H*), 6.98 (1H, d, *J* = 16.4 Hz, ArC*H*=CHPh), 7.08 (1H, d, *J* = 16.4 Hz, ArCH=C*H*Ph), 7.24–7.28 (1H, m, ArH), 7.36 (2H, t, *J* = 8.0 Hz, ArH), 7.46–7.51 (4H, m, ArH); ^13^C-NMR (100 MHz, CDCl_3_) δ 55.4 (CH_3_), 114.2 (CH), 126.4 (CH), 126.7 (CH), 127.3 (CH), 127.8 (CH), 128.3 (CH), 128.8 (CH), 130.2 (C), 137.8 (C), 159.4 (C). Spectral data of this compound were consistent with literature data [33].

#### 3.5.13. Data for Compound **20**


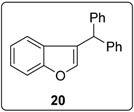


Pale yellow viscous oil. ν*_max_* (neat)/cm^−1^ 3084, 3060, 3027, 1601, 1496, 1454, 1282, 1186, 1098, 860, 700; ^1^H-NMR (400 MHz, CDCl_3_) δ 5.54 (1H, s, C*H*Ph_2_), 7.03 (1H, d, *J* = 1.6 Hz, OC*H*=C), 7.08–7.15 (2H, m, Ar*H*), 7.23–7.34 (11H, m, Ar*H*), 7.48 (1H, d, *J* = 8.4 Hz, ArH); ^13^C-NMR (100 MHz, CDCl_3_) δ 47.8 (CH), 111.6 (CH), 120.8 (CH), 122.5 (CH), 124.2 (C), 124.4 (CH), 126.9 (CH), 127.7 (C), 128.7 (CH), 128.9 (CH), 142.4 (C), 144.1 (C), 156.0 (C). Spectral data of this compound were consistent with literature data [13].

#### 3.5.14. Data for Compound **22**


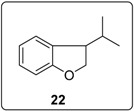


Colourless oil. ν*_max_* (neat)/cm^−1^ 3049, 2958, 2872, 1597, 1483, 1459, 1232, 1018, 748; ^1^H-NMR (400 MHz, CDCl_3_) δ 0.89 (3H, d, *J* = 7.2 Hz, C*H*_3_), 0.97 (3H, d, *J* = 6.8 Hz, C*H*_3_), 1.94–2.02 (1H, m, C*H*(CH_3_)_2_), 3.31–3.36 (1H, m, ArC*H*), 4.39 (1H, dd, *J* = 9.2, 5.2 Hz, ArOC*H*_2_), 4.53 (1H, t, *J* = 9.2 Hz, ArOC*H*_2_), 6.78 (1H, d, *J* = 8.0 Hz, Ar*H*), 6.86 (1H, td, *J* = 7.2, 0.8 Hz, Ar*H*), 7.11–7.15 (1H, m, Ar*H*), 7.19 (1H, d, *J* = 7.6 Hz, ArH); ^13^C-NMR (100 MHz, CDCl_3_) δ 18.6 (CH_3_), 19.9 (CH_3_), 31.8 (CH), 48.3 (CH), 74.0 (CH_2_), 109.5 (CH), 120.2 (CH), 125.2 (CH), 128.3 (CH), 129.6 (C), 160.6 (C). Spectral data of this compound were consistent with literature data [34].

#### 3.5.15. Data for Compound **6h**


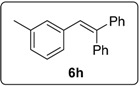


Colourless oil. ν*_max_* (neat)/cm^−1^ 3055, 3021, 2921, 1603, 1493, 1446, 1076, 1031, 696; ^1^H-NMR (500 MHz, CDCl_3_) δ 2.20 (3H, s, C*H*_3_), 6.81 (1H, d, *J* = 8.0 Hz, ArH), 6.87 (1H, s, ArC*H*=), 6.94 (1H, t, *J* = 7.5 Hz, ArH), 6.96 (1H, s, Ar*H*), 7.02 (1H, t, *J* = 7.5 Hz, ArH), 7.21–7.23 (2H, m, ArH), 7.28–7.36 (8H, m, ArH); ^13^C-NMR (100 MHz, CDCl_3_) δ 21.5 (CH_3_), 126.7 (CH), 127.5 (CH), 127.6 (CH), 127.67 (CH), 127.7 (CH), 127.9 (CH), 128.3 (CH), 128.4 (CH), 128.7 (CH), 130.5 (CH), 130.6 (CH), 137.4 (C), 137.5 (C), 140.6 (C), 142.5 (C), 143.6 (C). Spectral data of this compound were consistent with literature data [13].

#### 3.5.16. Data for Compound **15h**


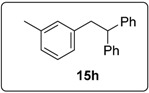


Colourless oil. ν*_max_* (neat)/cm^−1^ 3060, 3027, 2921, 1603, 1495, 1452, 1081, 1033, 698; [Found: (CI^+^ corona) (M+NH_4_)^+^ 290.1899. C_21_H_24_N (M+NH_4_) requires 290.1903]; ^1^H-NMR (500 MHz, CDCl_3_) δ 2.25 (3H, s, C*H*_3_), 3.34 (2H, d, *J* = 8.0 Hz, ArC*H*_2_CH), 4.24 (1H, t, *J* = 8.0 Hz, ArCH_2_C*H*), 6.80 (1H, d, *J* = 7.5 Hz, ArH), 6.84 (1H, s, ArH), 6.95 (1H, d, *J* = 7.5 Hz, ArH), 7.06 (1H, t, *J* = 7.5 Hz, ArH), 7.15–7.19 (2H, m, ArH), 7.21–7.28 (8H, m, ArH); ^13^C-NMR (100 MHz, CDCl_3_) δ 21.5 (CH_3_), 42.2 (CH_2_), 53.2 (CH), 126.2 (CH), 126.3 (CH), 126.7 (CH), 128.0 (CH), 128.2 (CH), 128.4 (CH), 130.1 (CH), 137.6 (C), 140.3 (C), 144.7 (2 × C); *m/z* (CI^+^ corona) 290 [(M+NH_4_)^+^, 100%], 271 [(M−H)^+^, 28%], 195 (33), 167 (70), 111 (12).

## 4. Conclusions

Coupling of iodoarenes to diphenylethenes and to styrenes can be triggered by *N,N'*-dialkyl diketopiperazines in the presence of KO*t*Bu. The reaction products can be largely determined by the number of equivalents of KO*t*Bu and of the *N,N'*-dialkyl diketopiperazine. Increasing the amount of butoxide drives the products towards stilbene-type products, whereas increasing the number of equivalents of the *N,N'*-dialkyl diketopiperazine while decreasing the number of equivalents of KO*t*Bu diverts the reaction towards dihydrostilbenes.

## Supplementary Materials

Supplementary materials can be accessed at: http://www.mdpi.com/1420-3049/20/02/1755/s1.
