# Supplementary material for: Electron Transfer-Induced Coupling of Haloarenes to Styrenes and 1,1-Diphenylethenes Triggered by Diketopiperazines and Potassium tert-Butoxide
Source: Molecules. 2015 Jan 22;20(2):1755–74. doi: 10.3390/molecules20021755 (PMC6272703; doi:10.3390/molecules20021755)
Supplement: Supplementary file 1 [file molecules-20-01755-s001.pdf]

Supplementary Materials

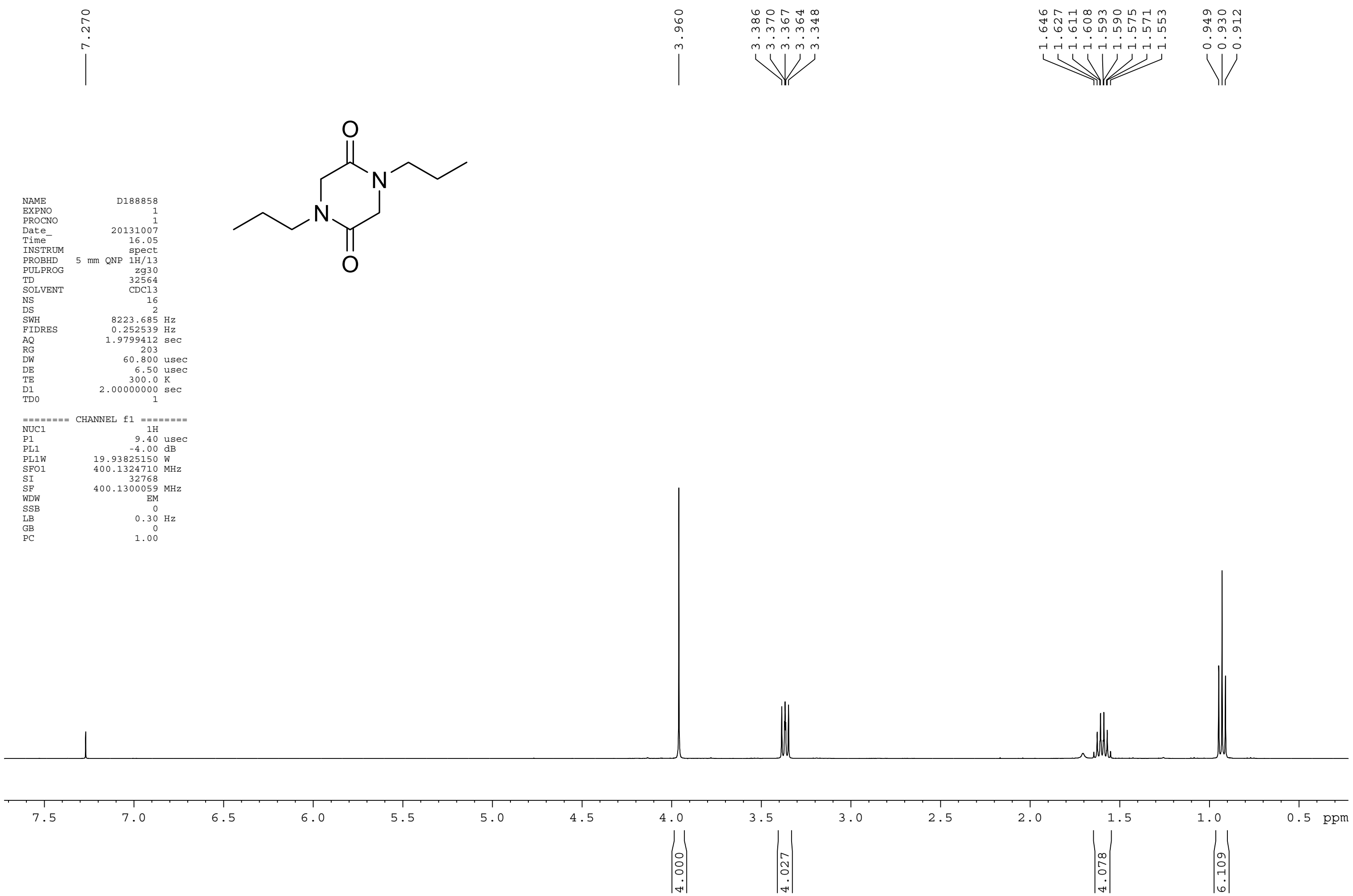

Figure S1. ^{1}H spectrum—compound **16**.

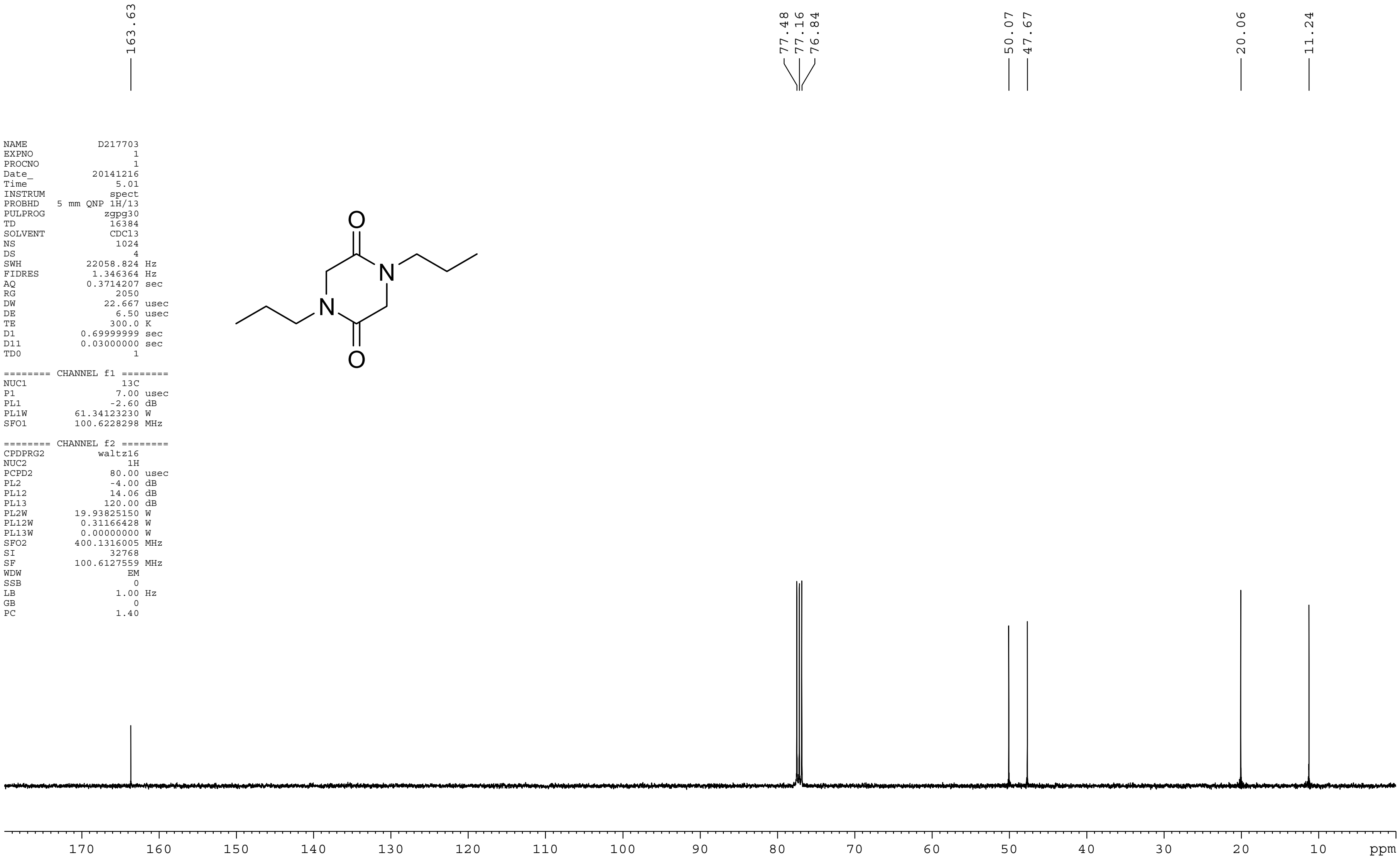

Figure S2. ^{13}C spectrum—compound **16**.

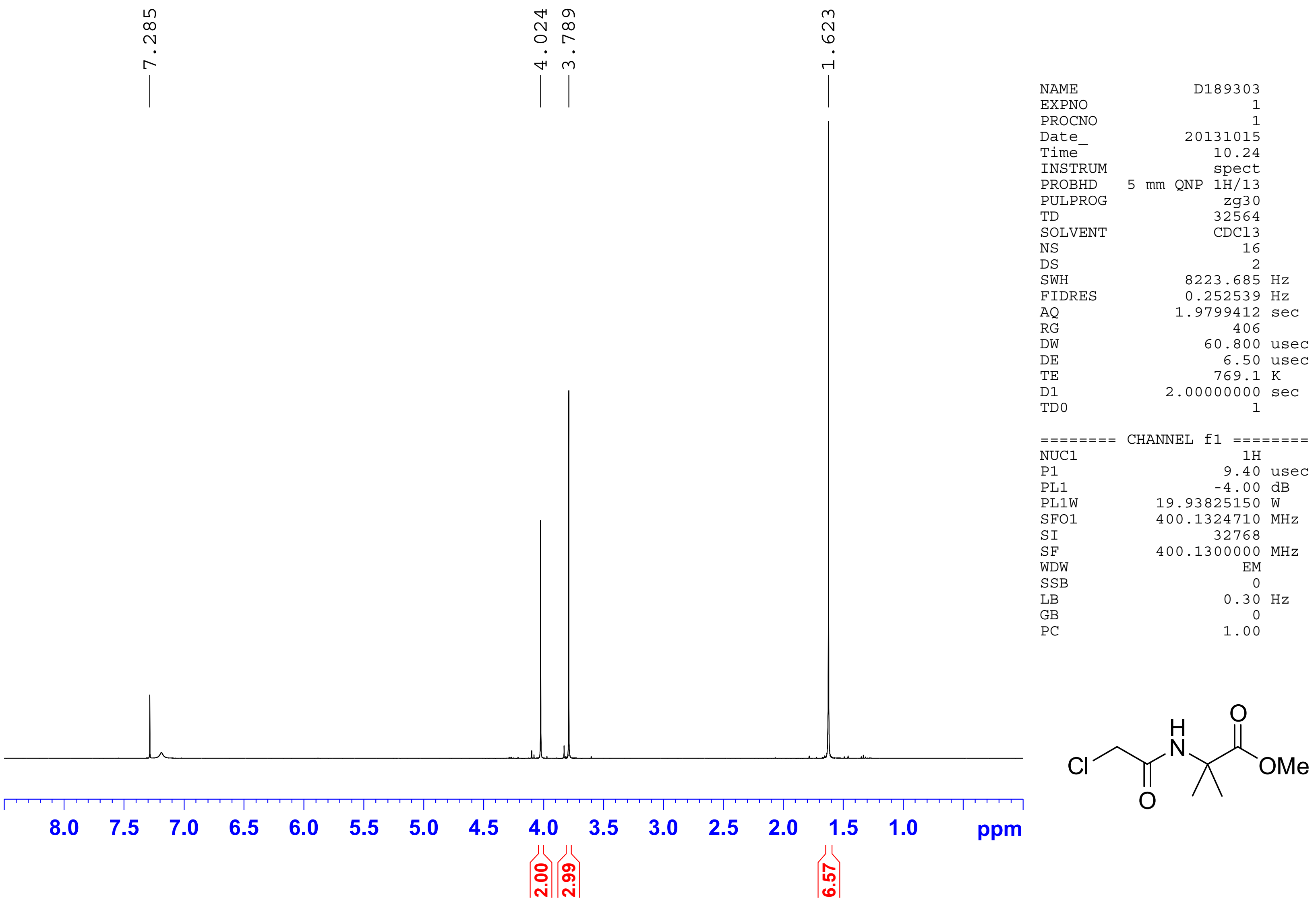

Figure S3. ^{1}H spectrum—intermediate 1 on way to compound **29**.

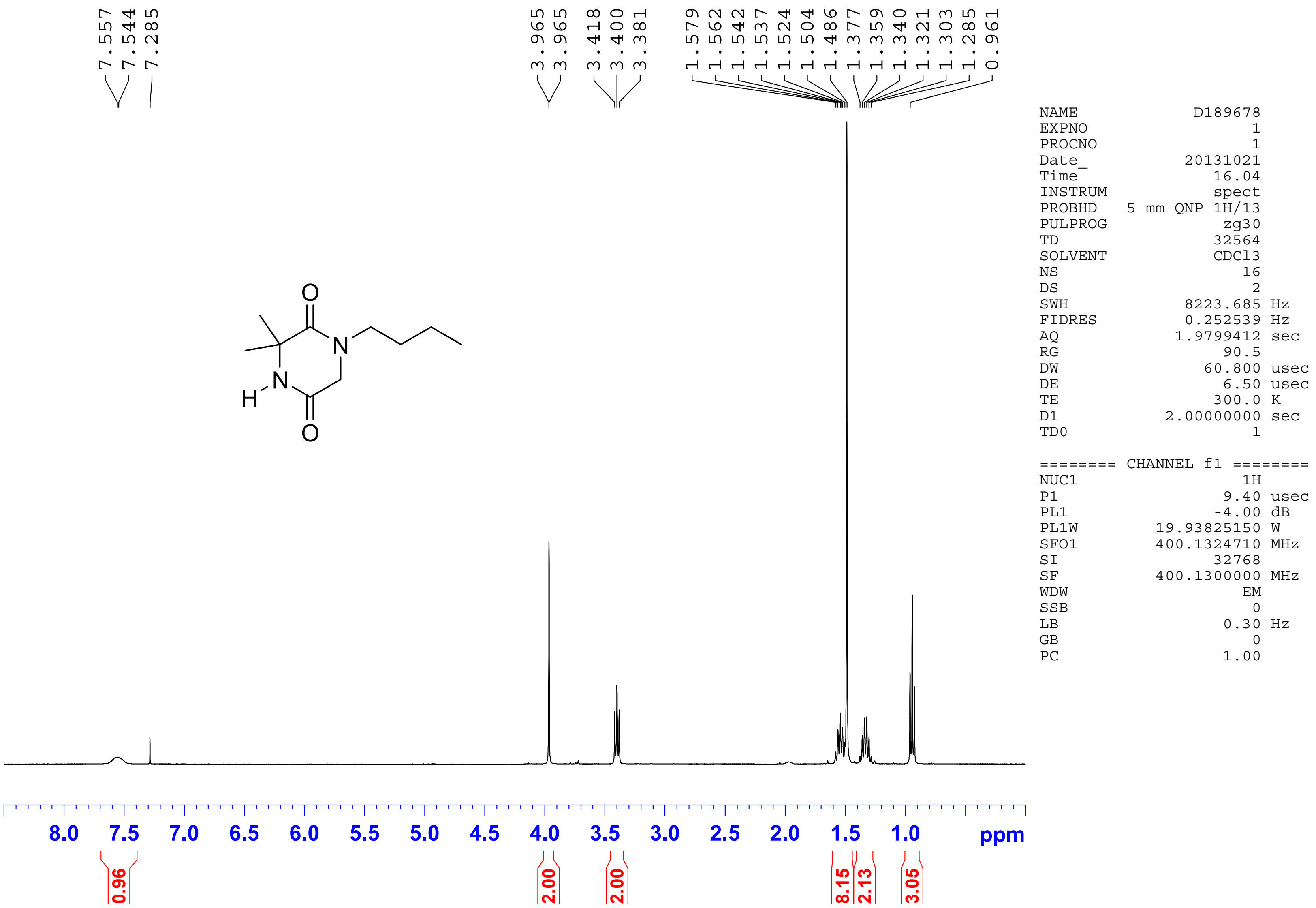

Figure S4. ^{1}H spectrum—intermediate 2 on way to compound **29**.

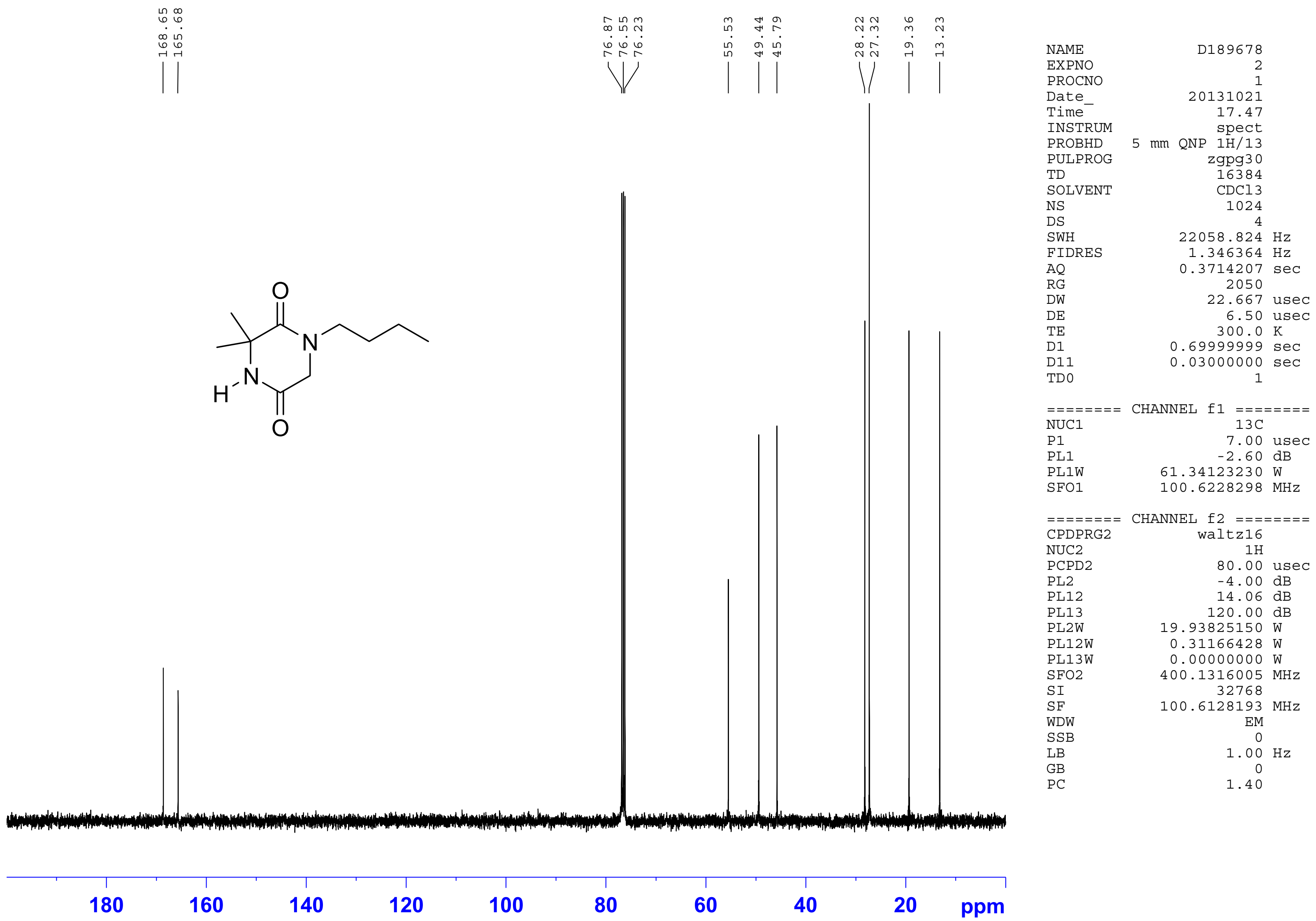

Figure S5. ^{13}C spectrum—intermediate 2 on way to compound **29**.

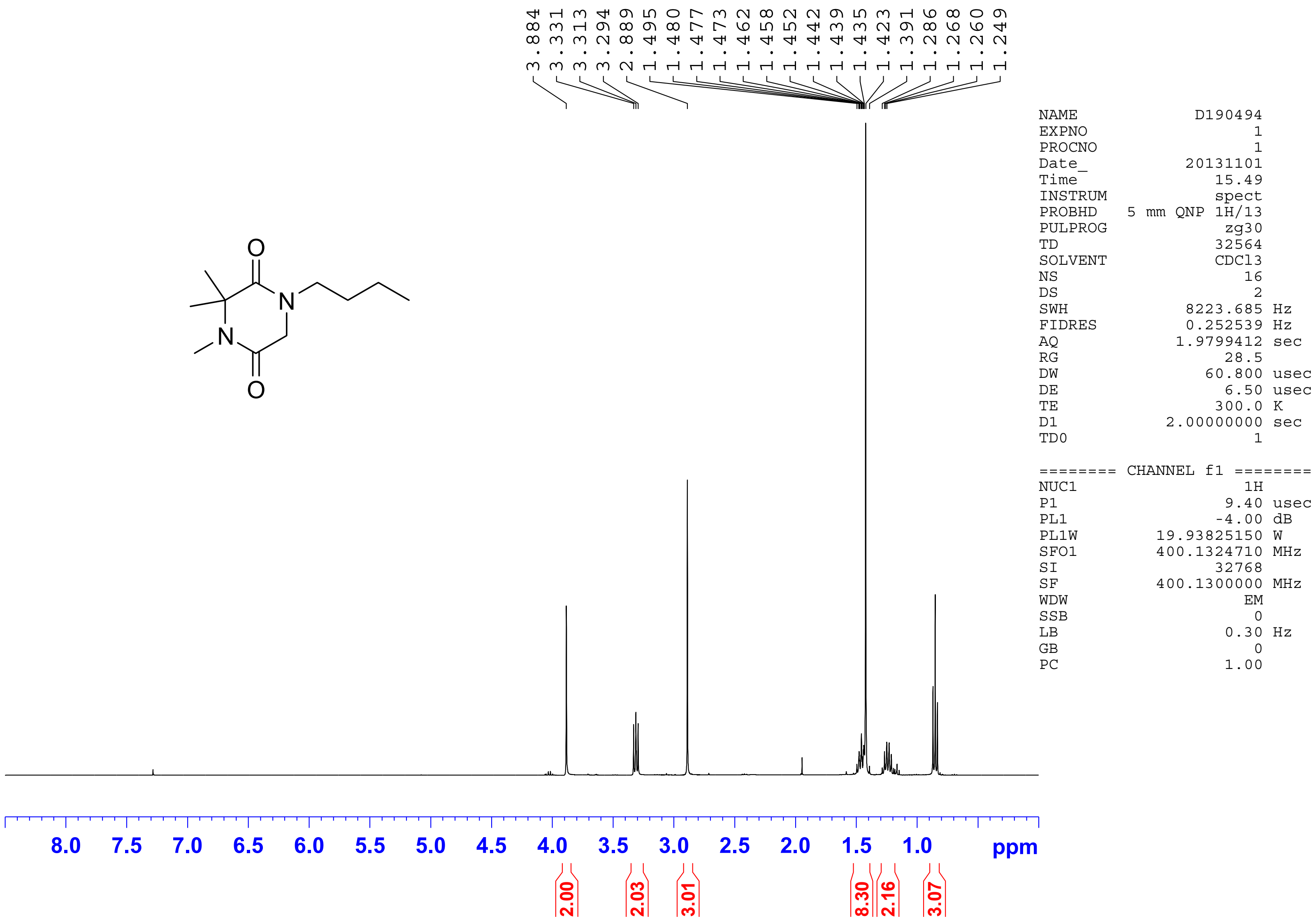

Figure S6. ^{1}H spectrum—compound **29**.

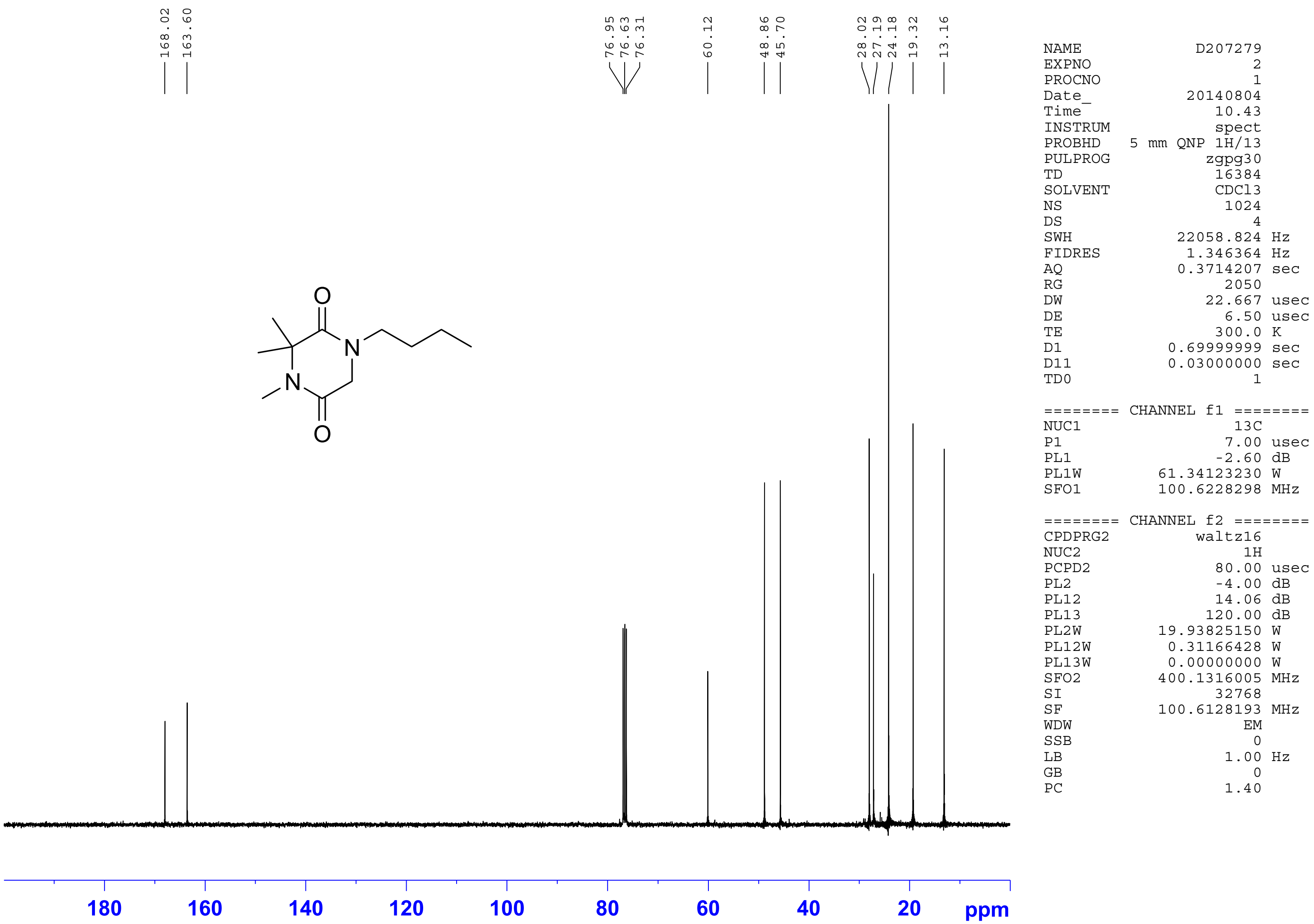

Figure S7. ^{13}C spectrum—compound **29**.

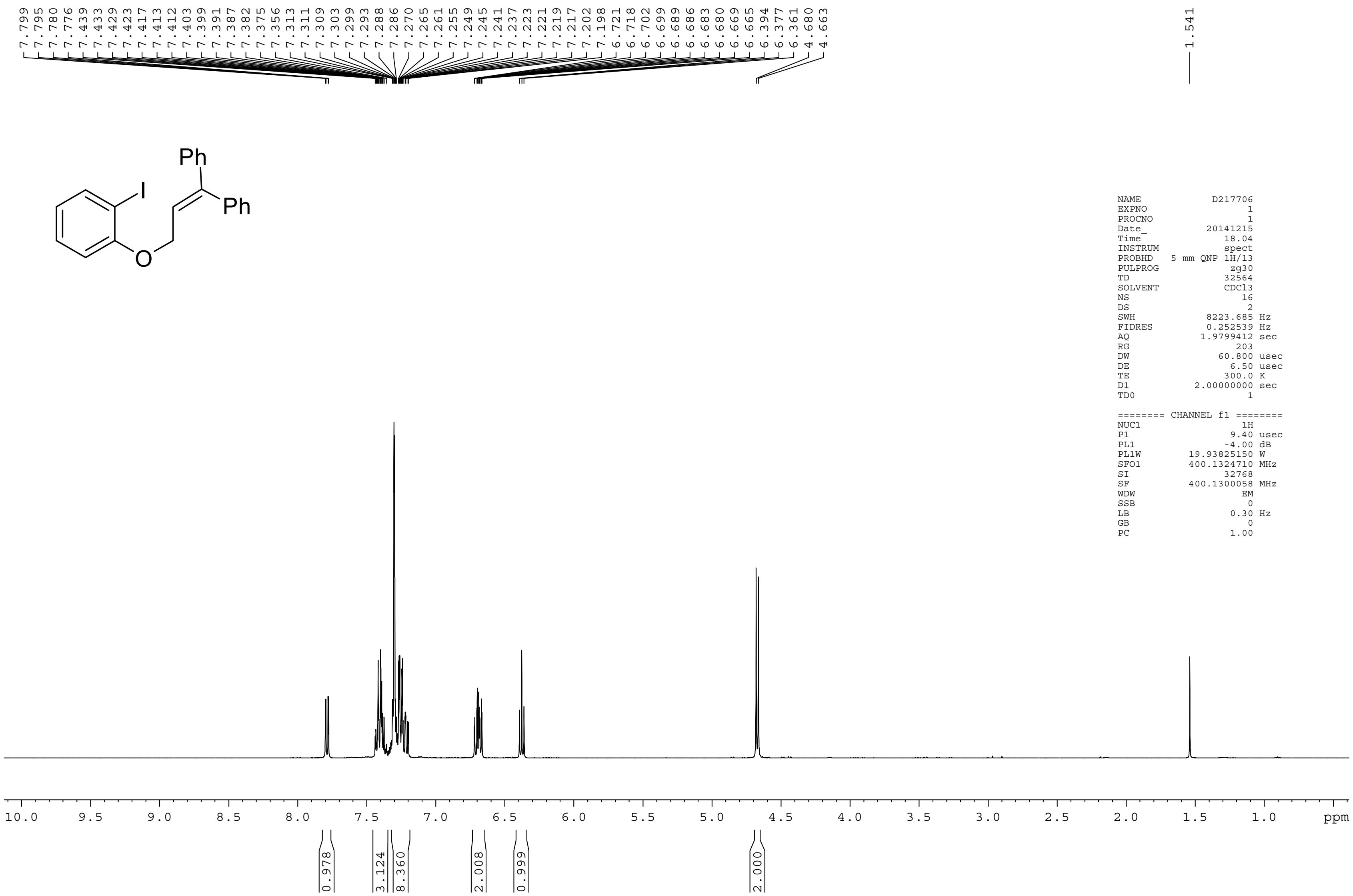

Figure S8. ^{1}H spectrum—compound **20**.

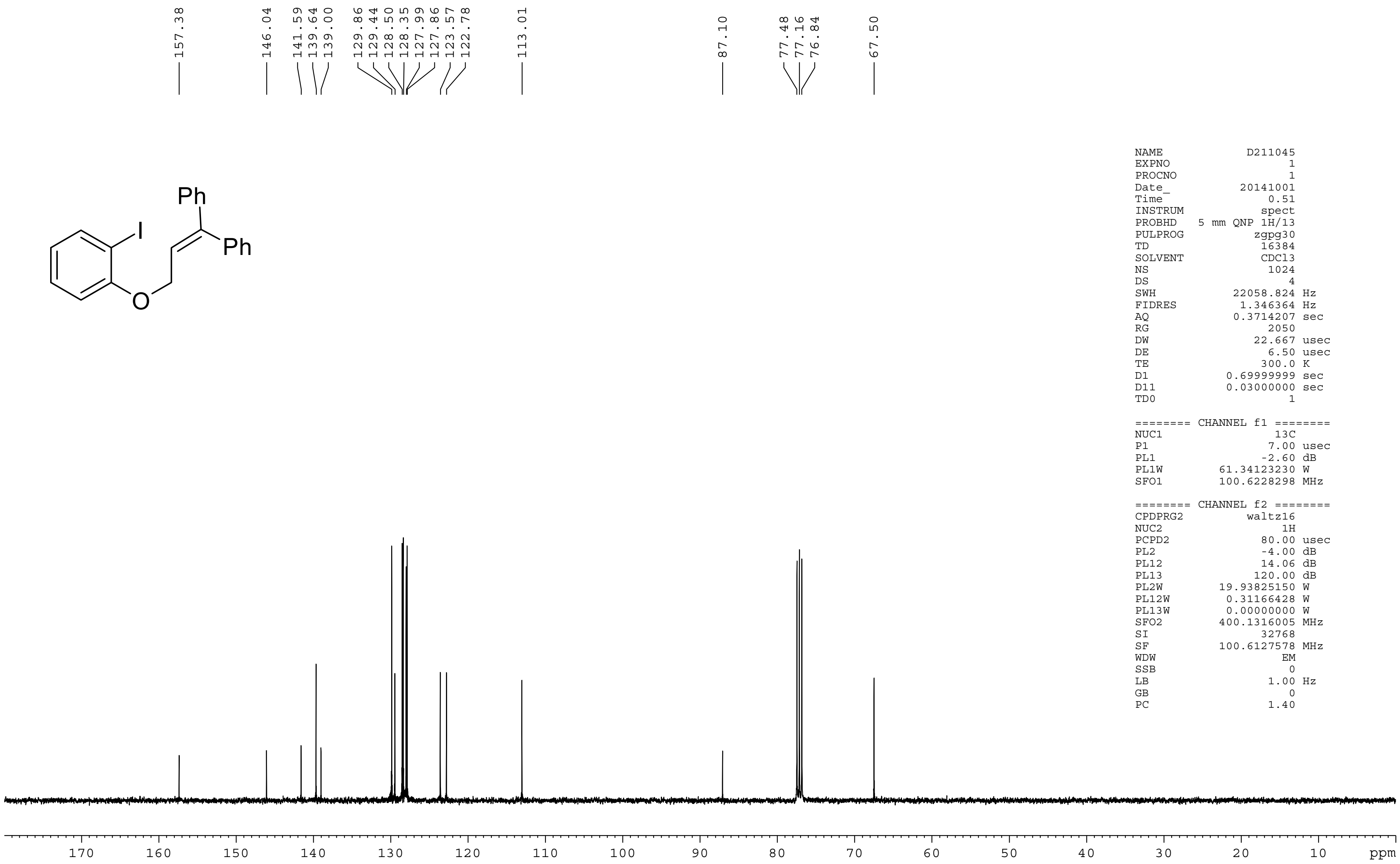

Figure S9. ^{13}C spectrum—compound **20**.

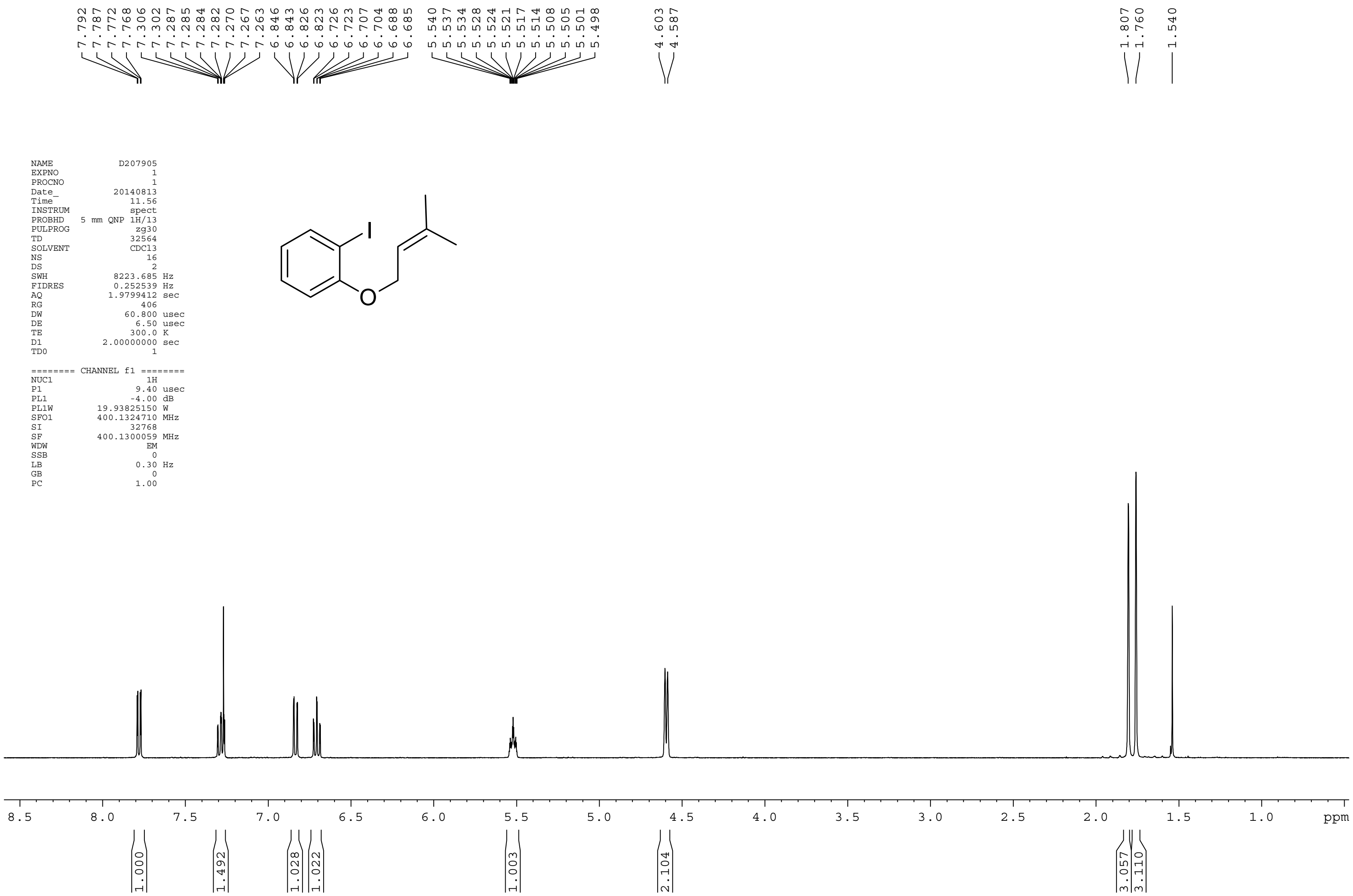

Figure S10. ^{1}H spectrum—compound **23**.

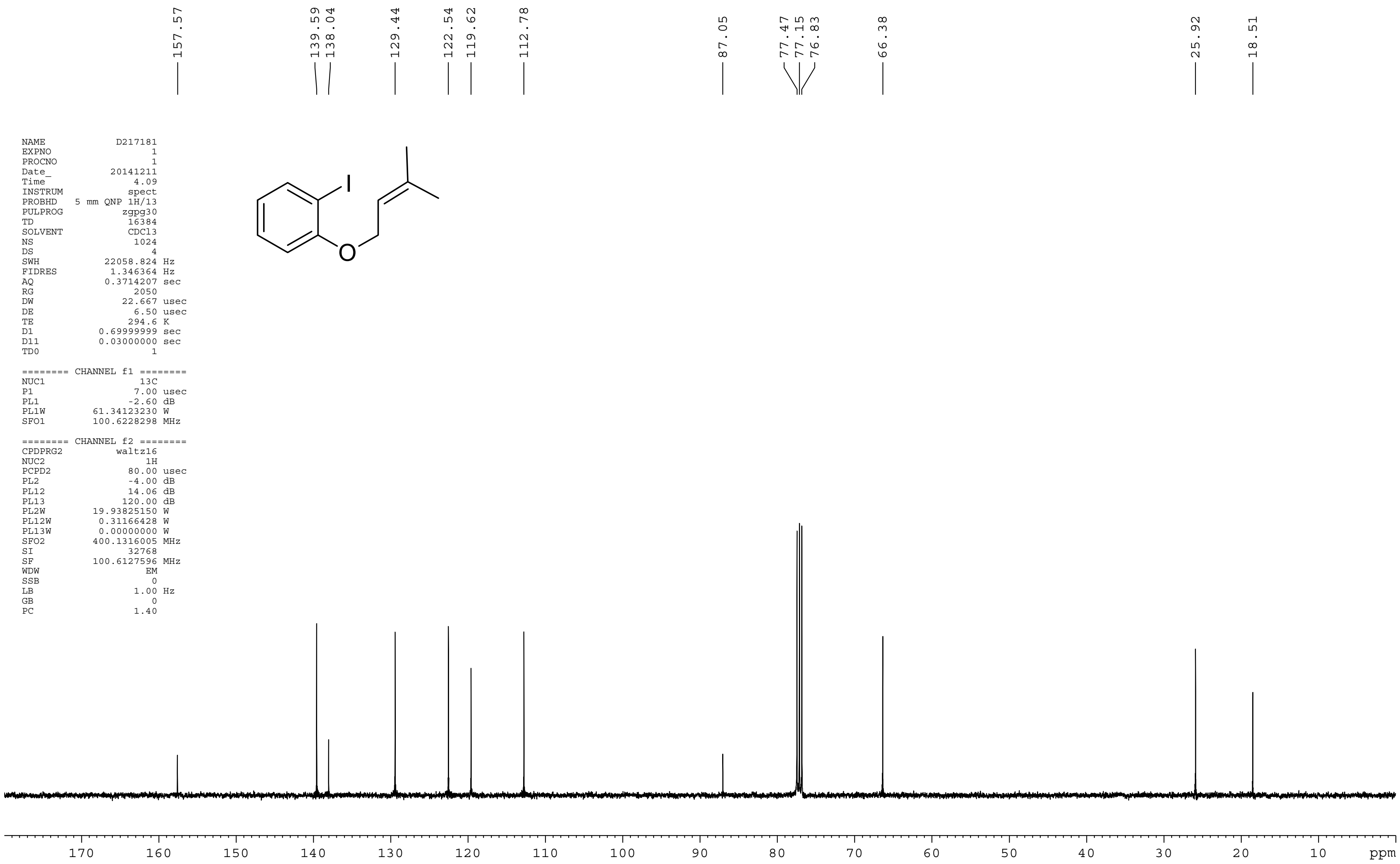

Figure S11. ^{13}C spectrum—compound **23**.

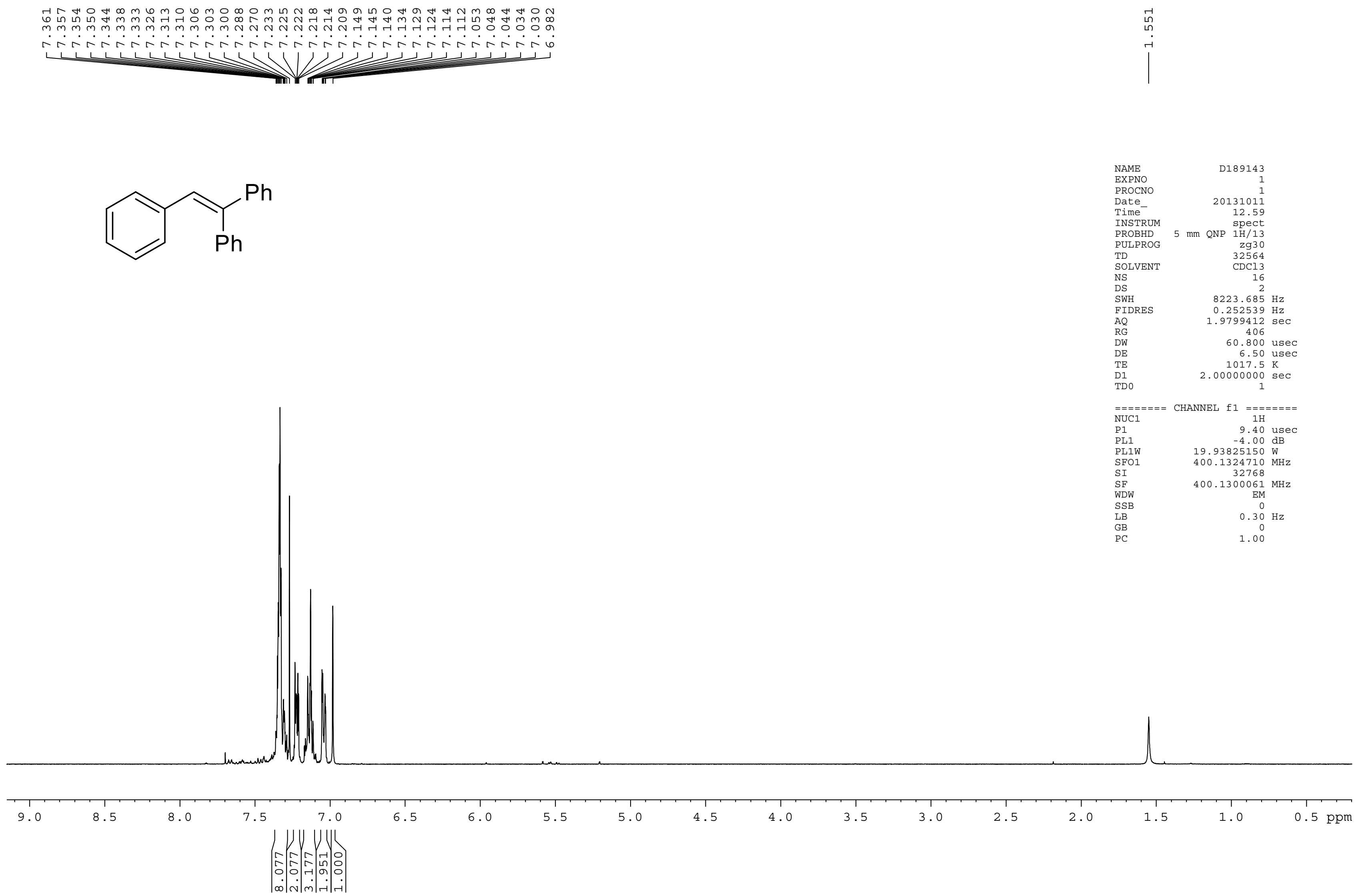

Figure S12. ^{1}H spectrum—compound **6a**.

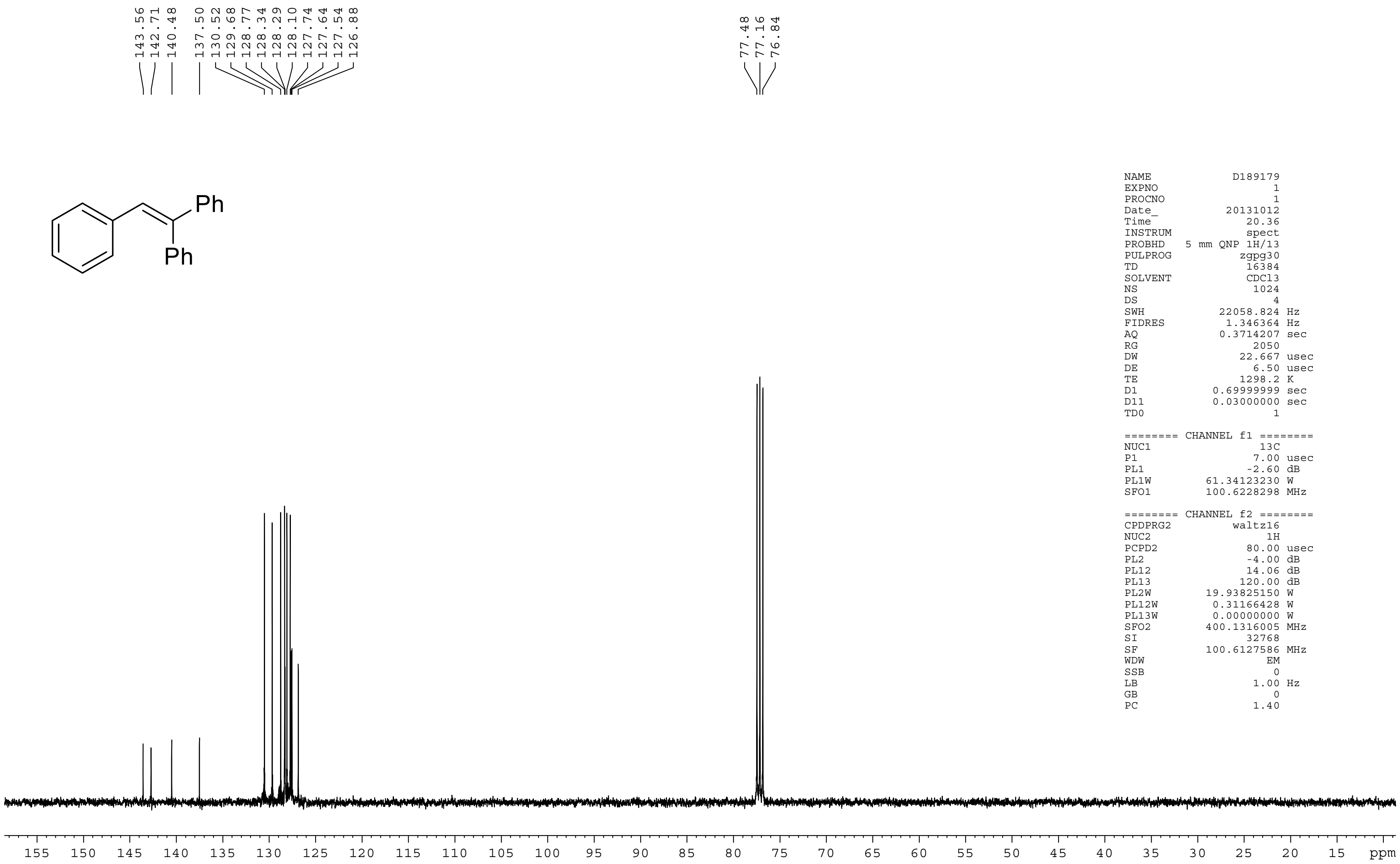

Figure S13. ^{13}C spectrum—compound **6a**.

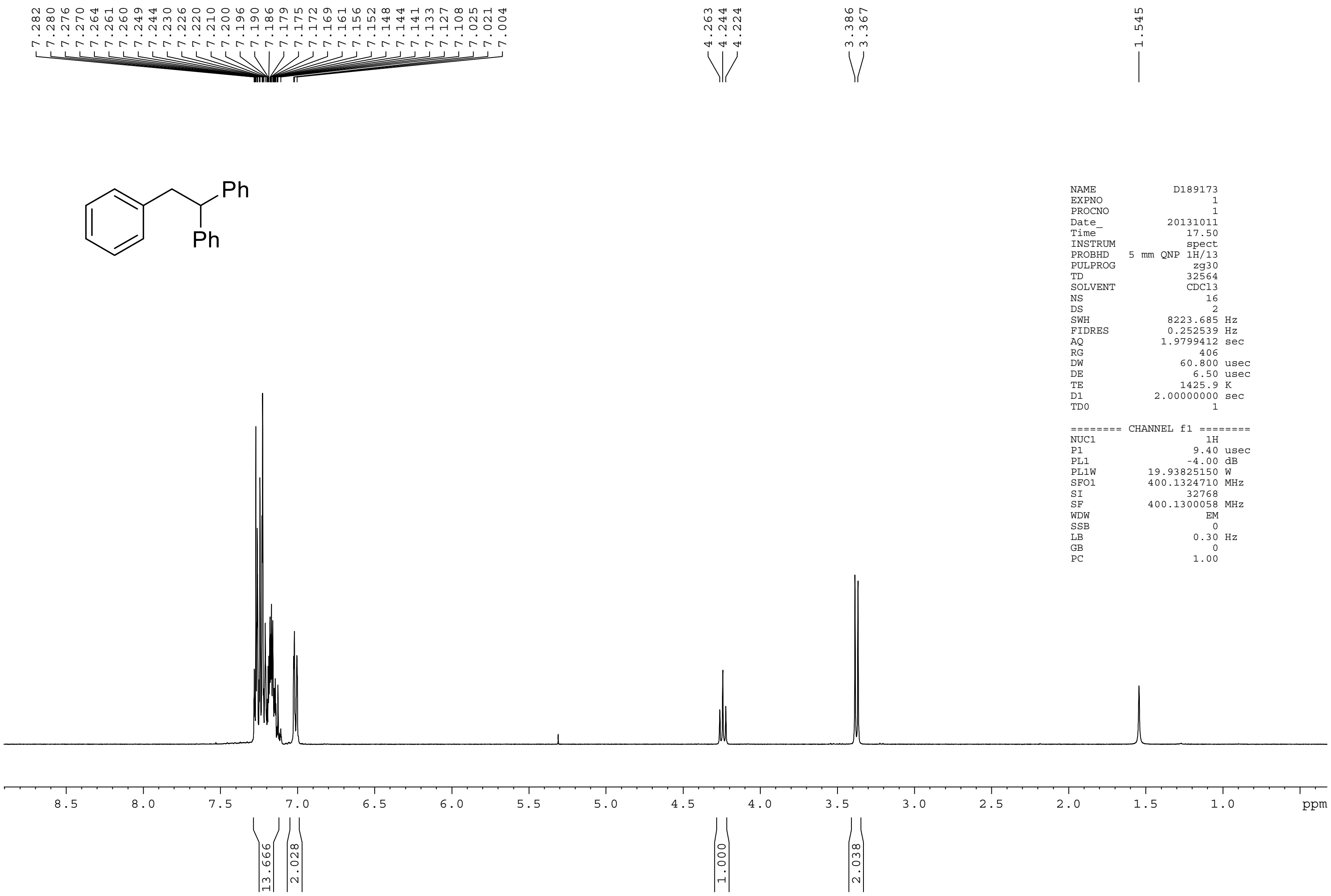

Figure S14. ^{1}H spectrum—compound **15a**.

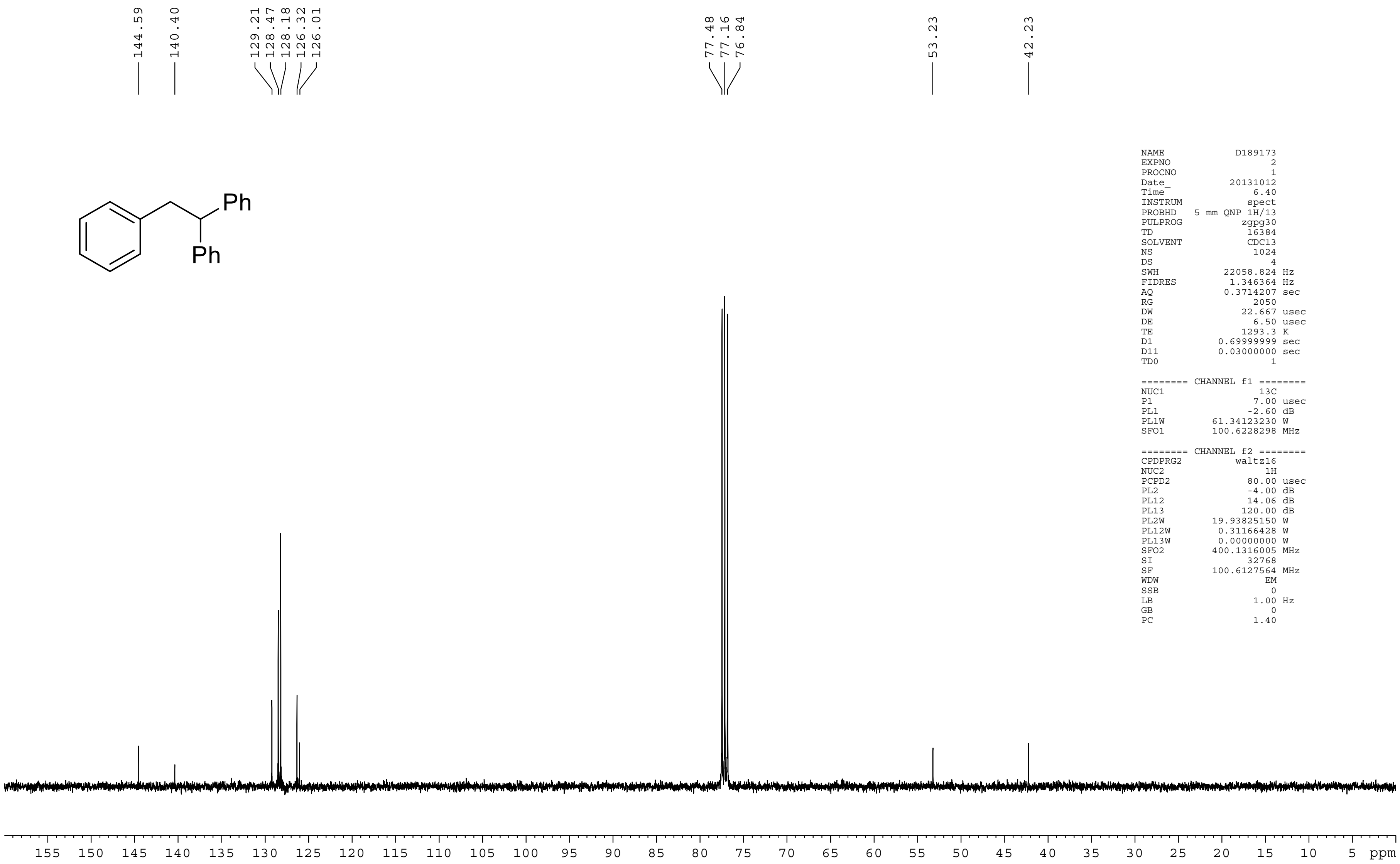

Figure S15. ^{13}C spectrum—compound **15a**.

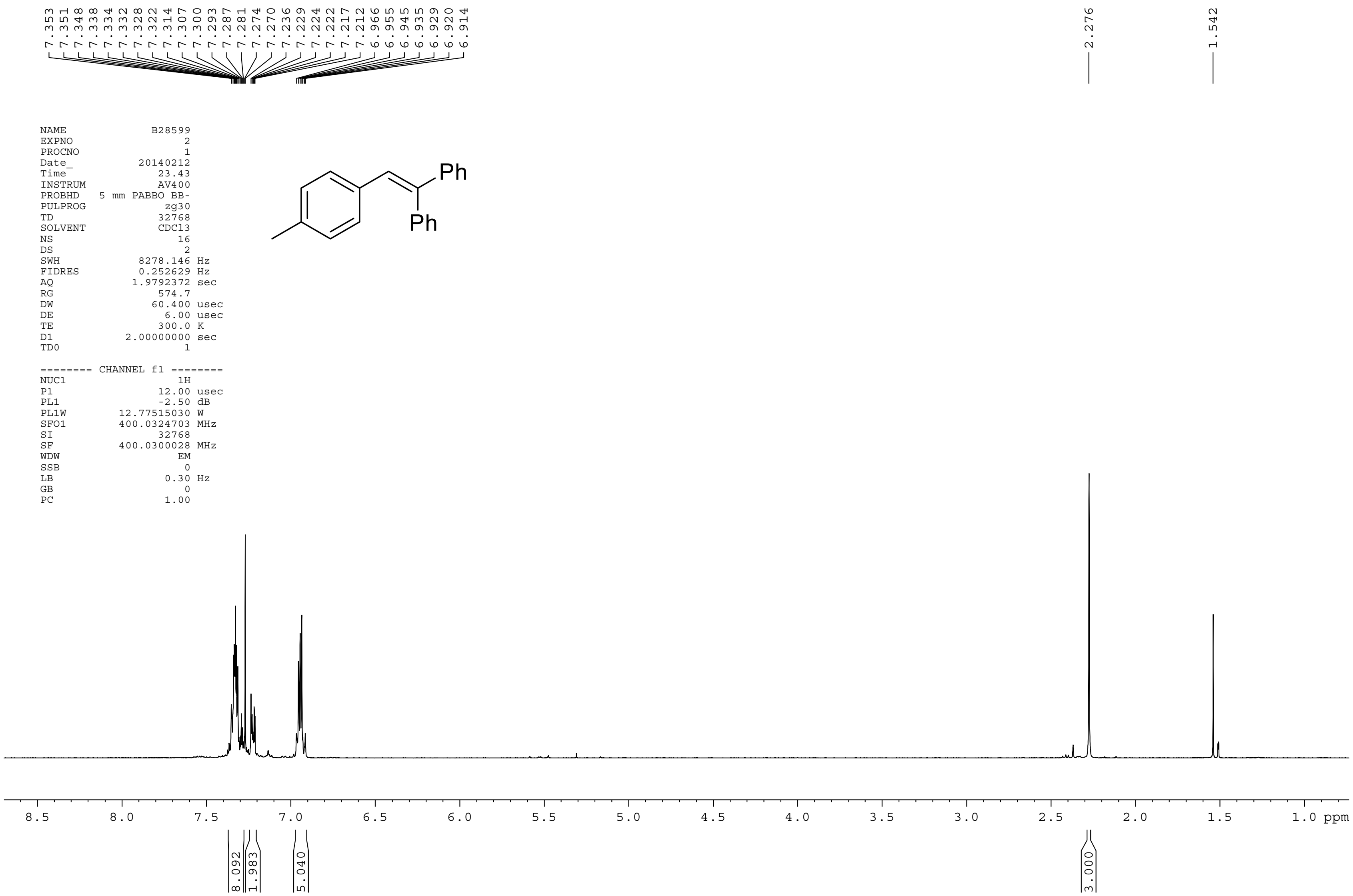

Figure S16. ^{1}H spectrum—compound **6b**.

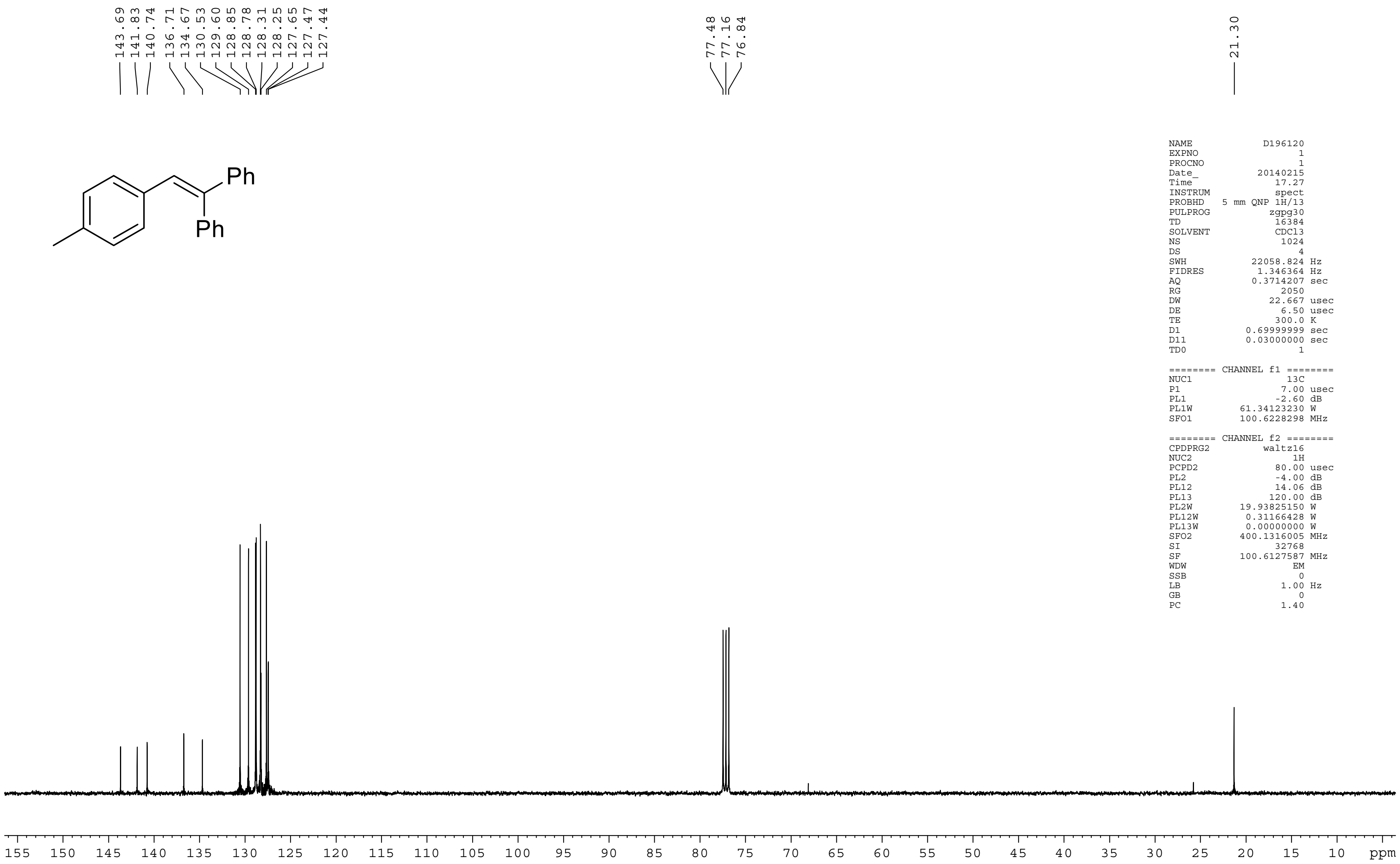

Figure S17. ^{13}C spectrum—compound **6b**.

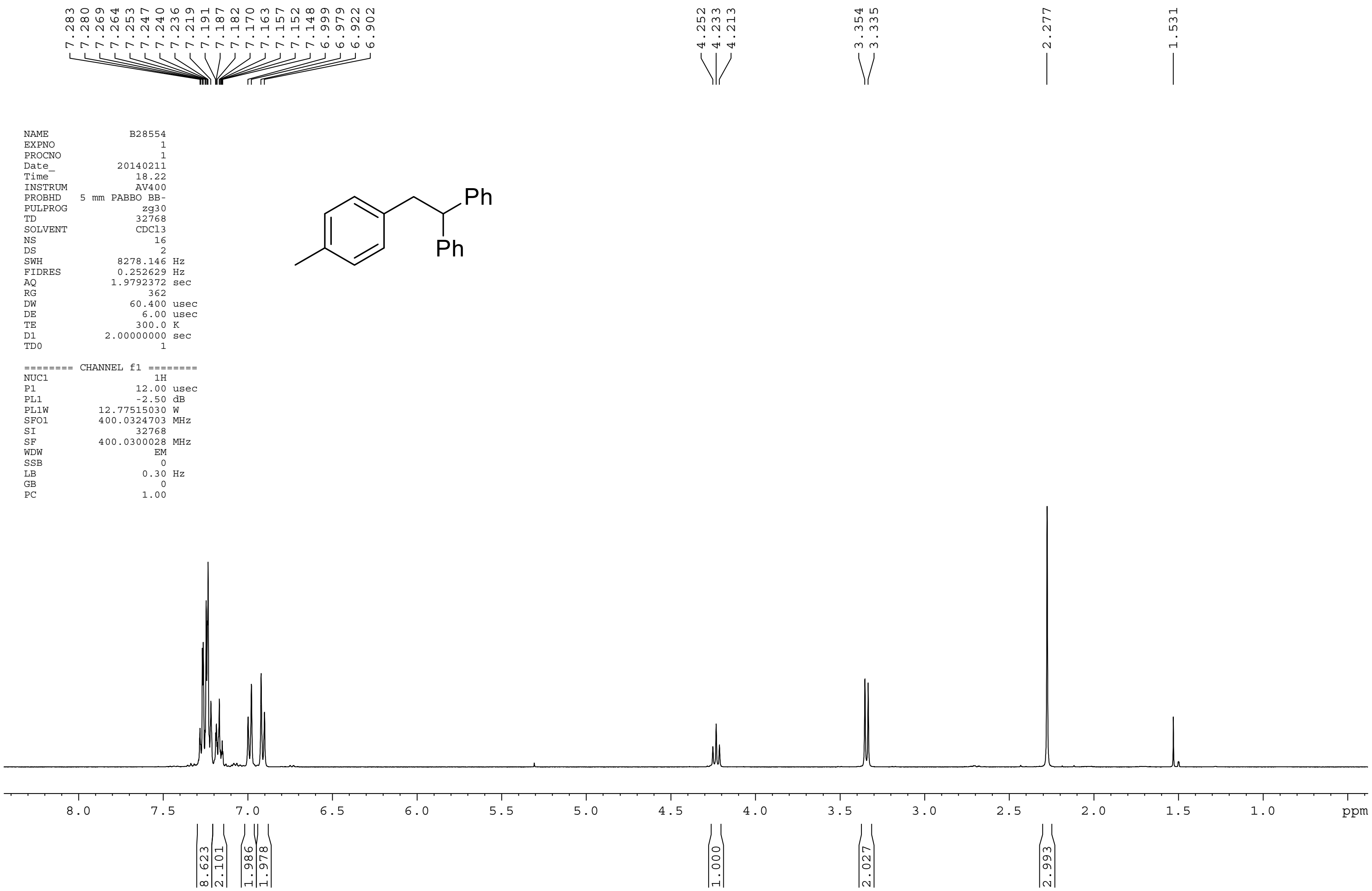

Figure S18. ^{1}H spectrum—compound **15b**.

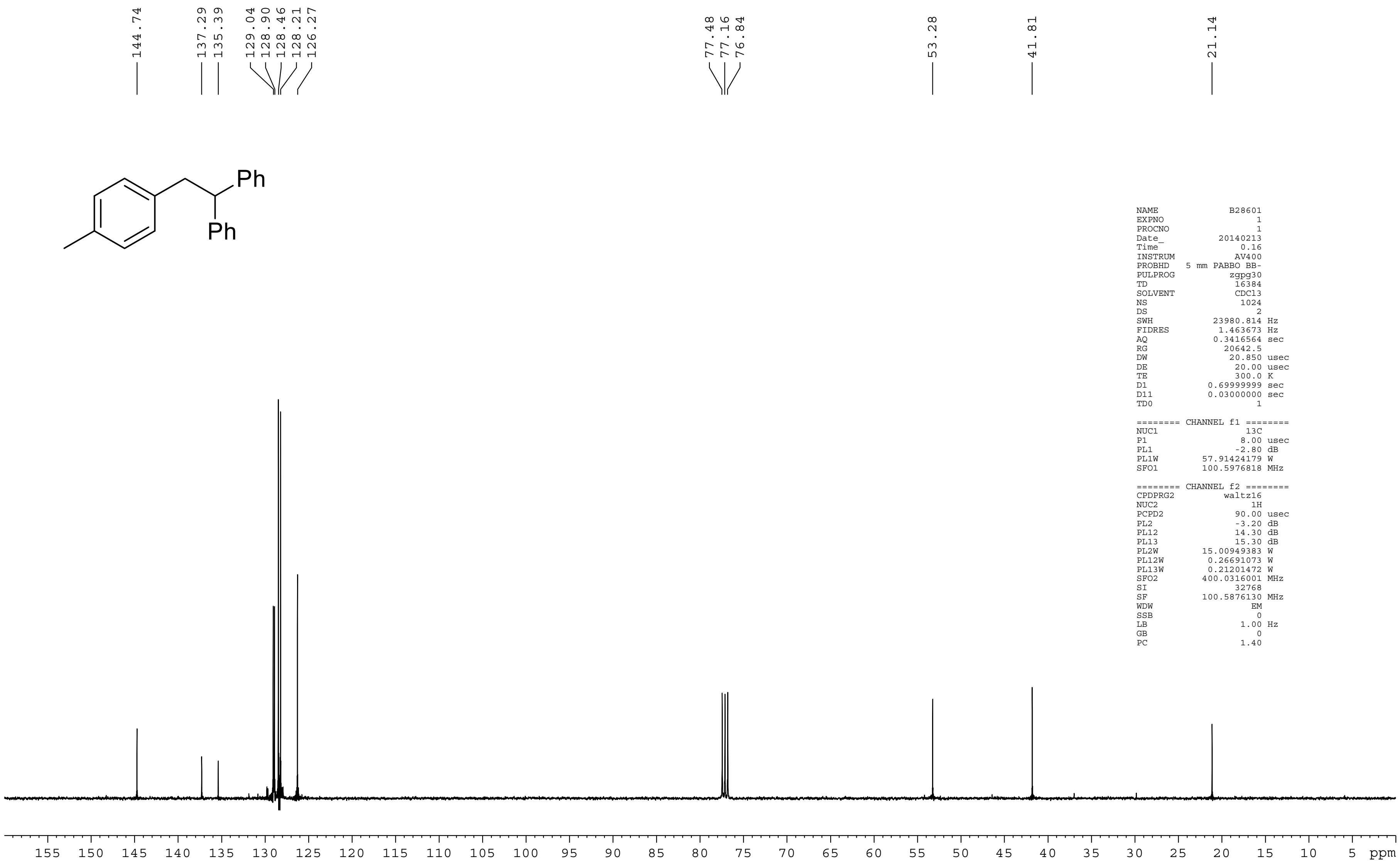

Figure S19. ^{13}C spectrum—compound **15b**.

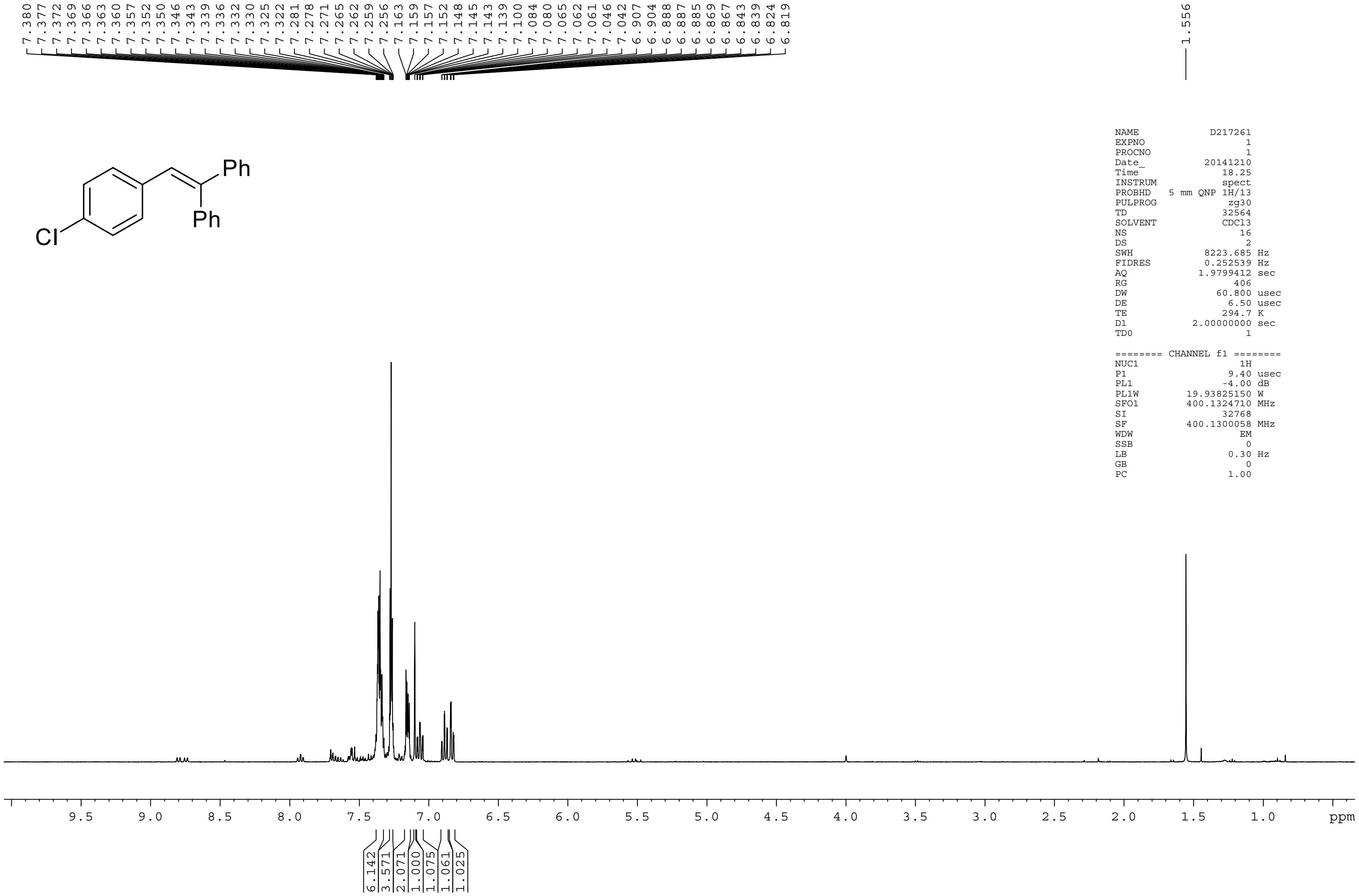

Figure S20. ^{1}H spectrum—compound **6e**.

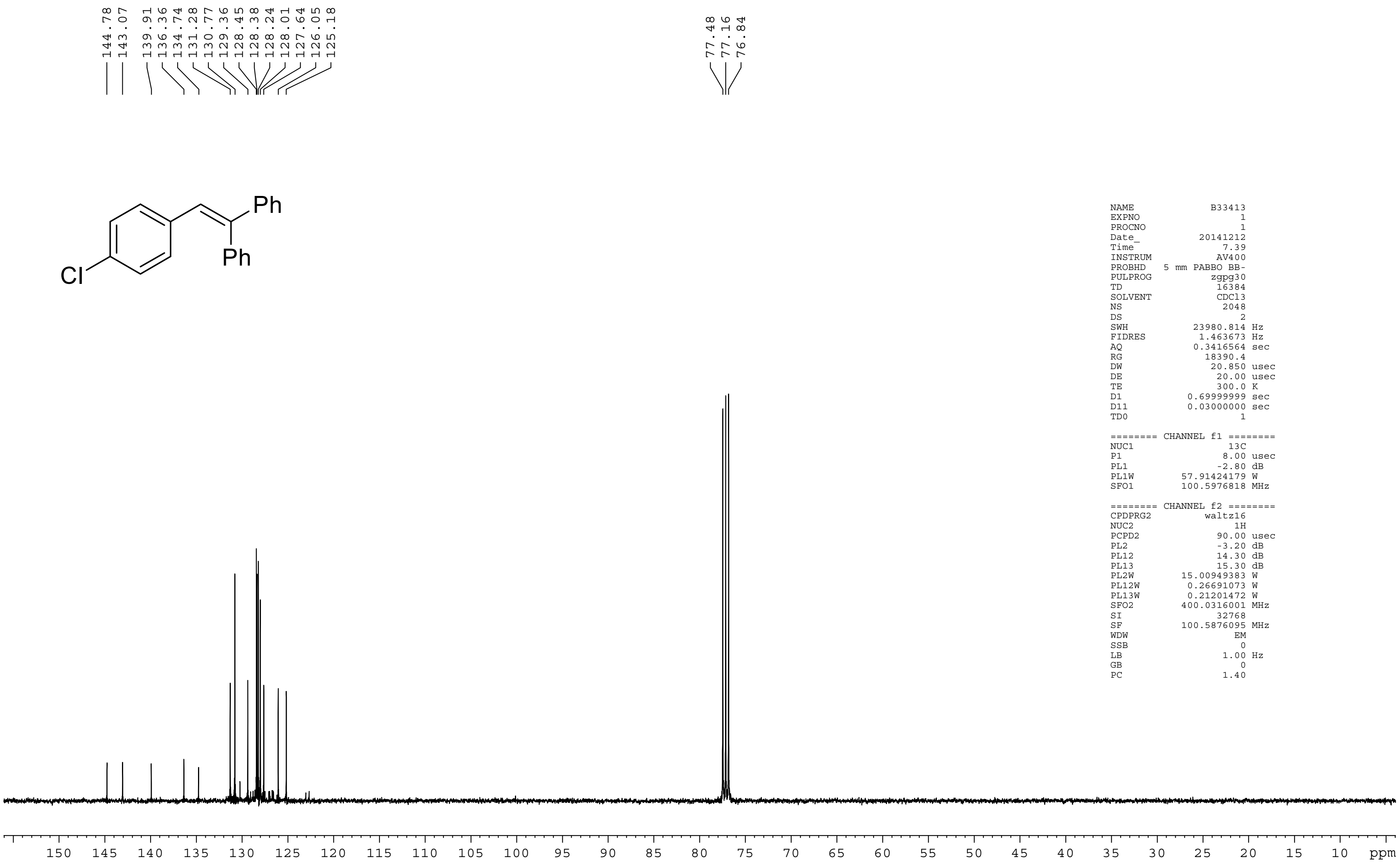

Figure S21. ^{13}C spectrum—compound **6e**.

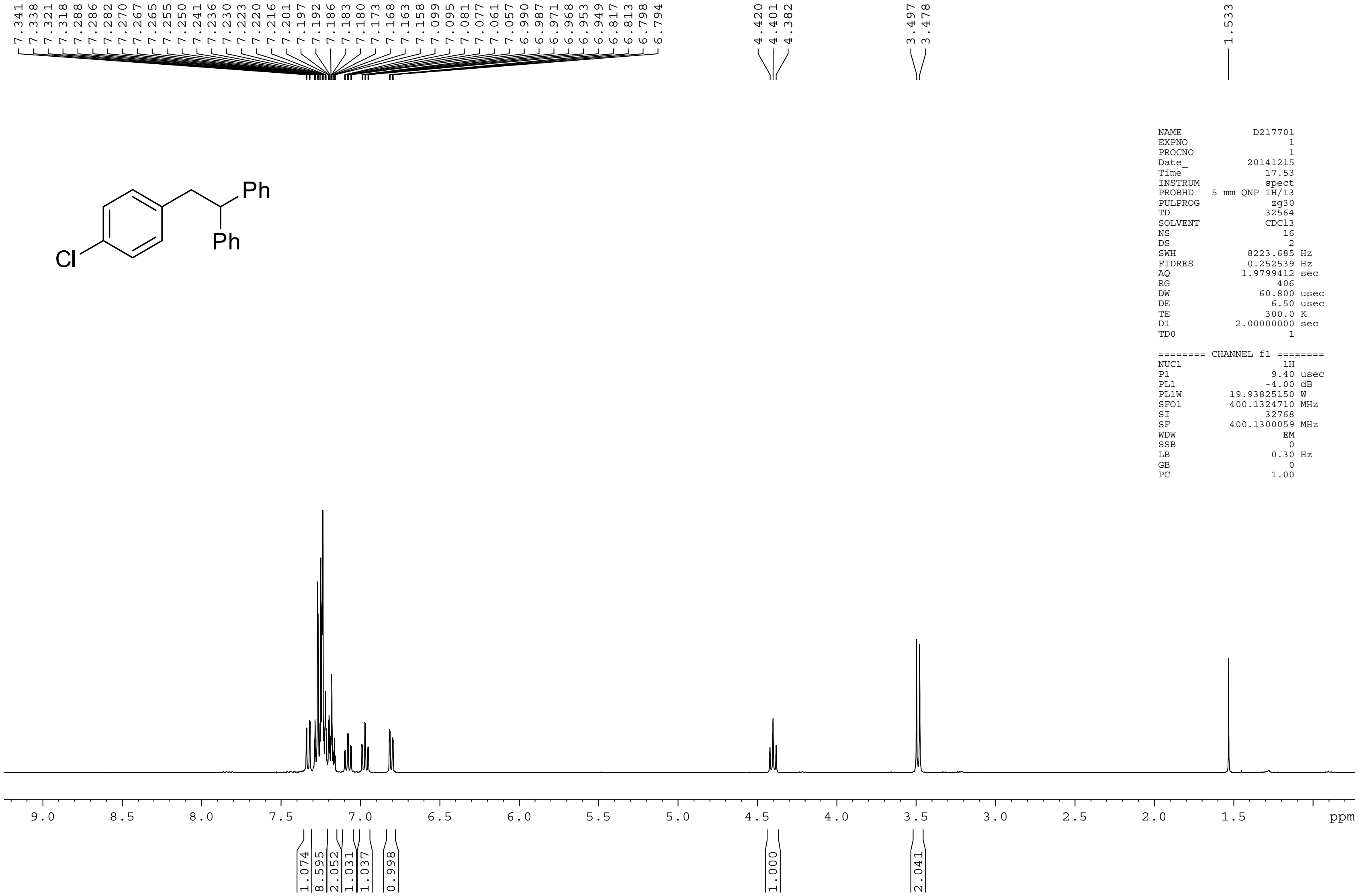

Figure S22. ^{1}H spectrum—compound **15e**.

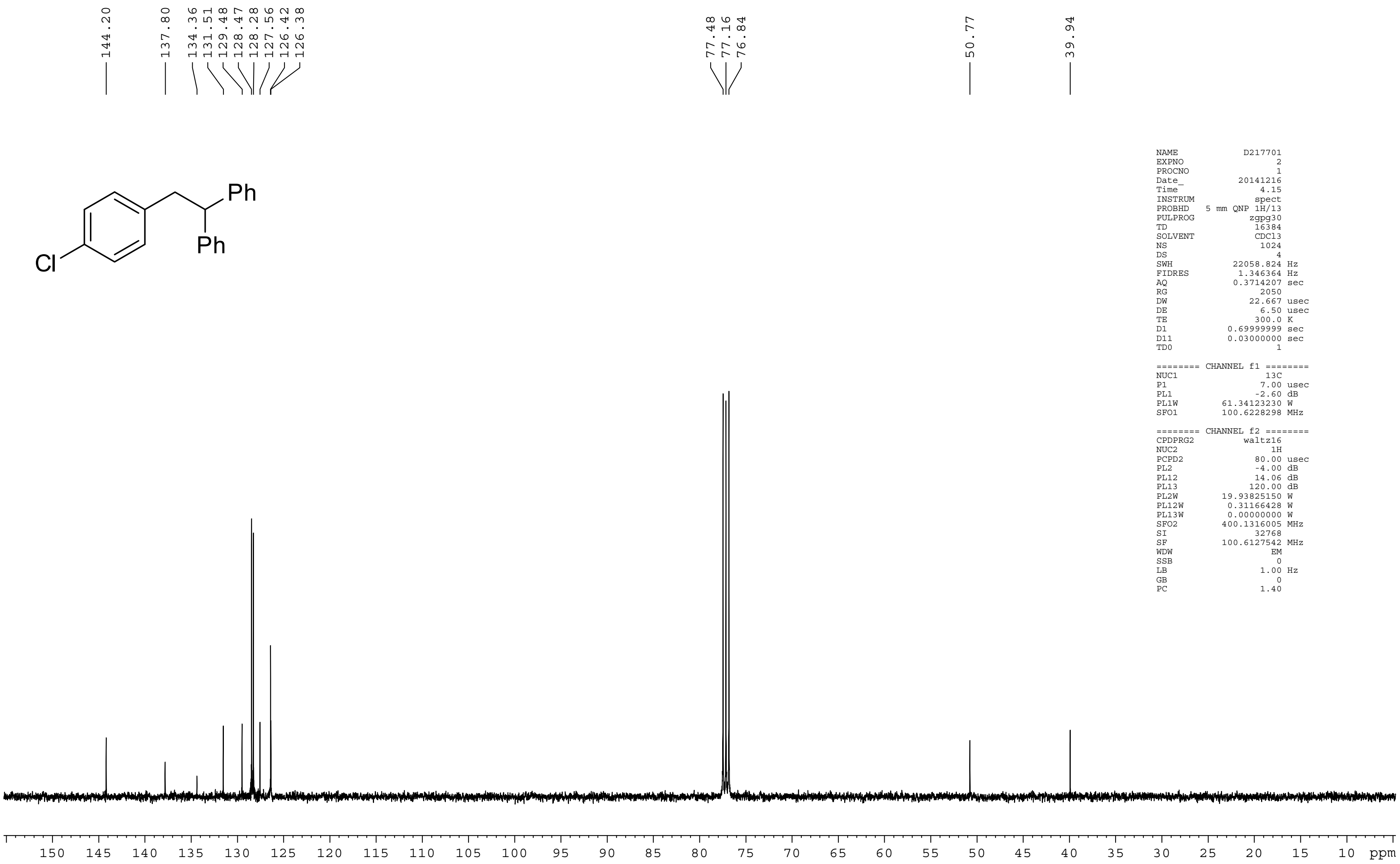

Figure S23. ^{13}C spectrum—compound **15e**.

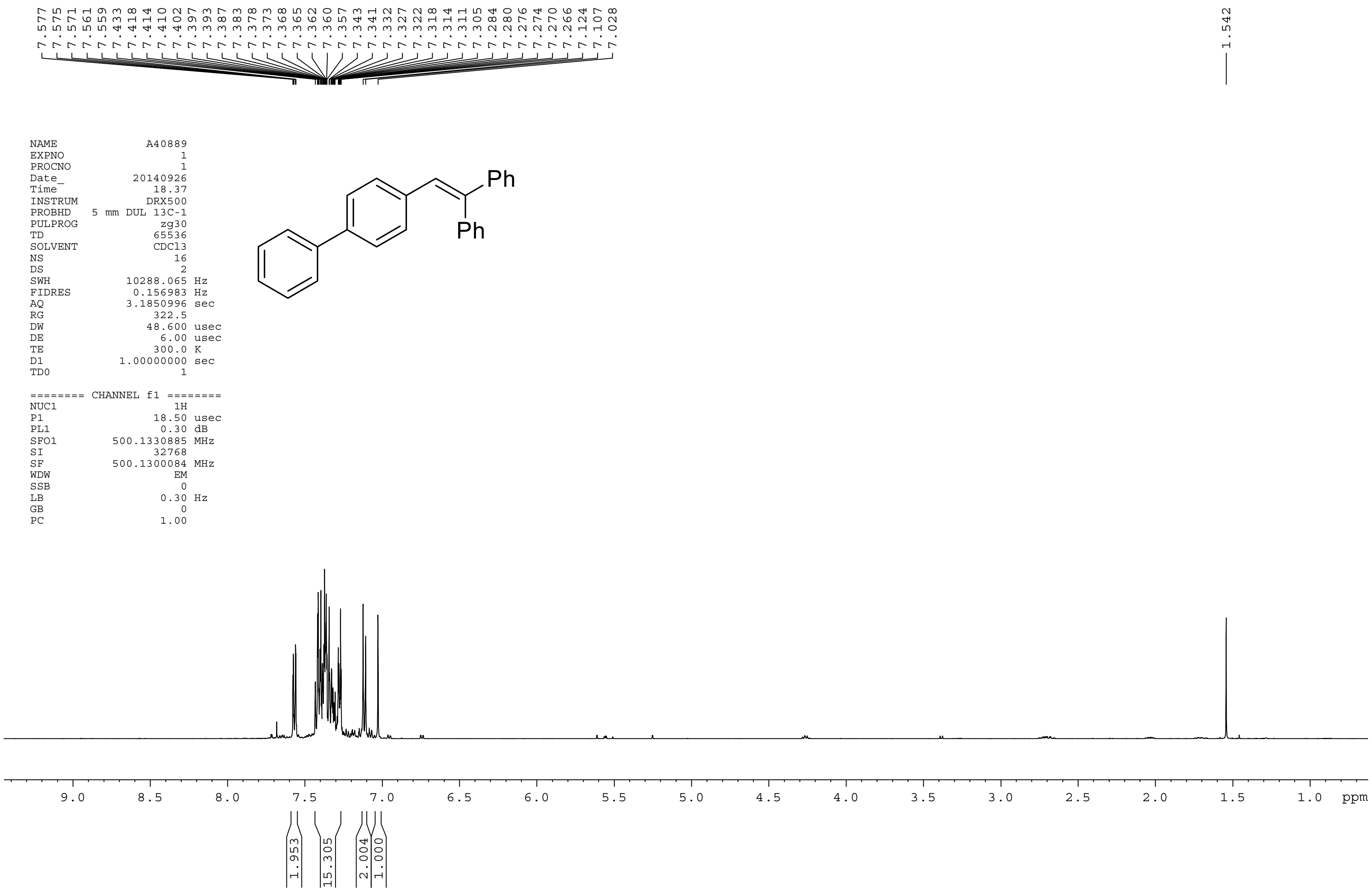

Figure S24. ^{1}H spectrum—compound **6f**.

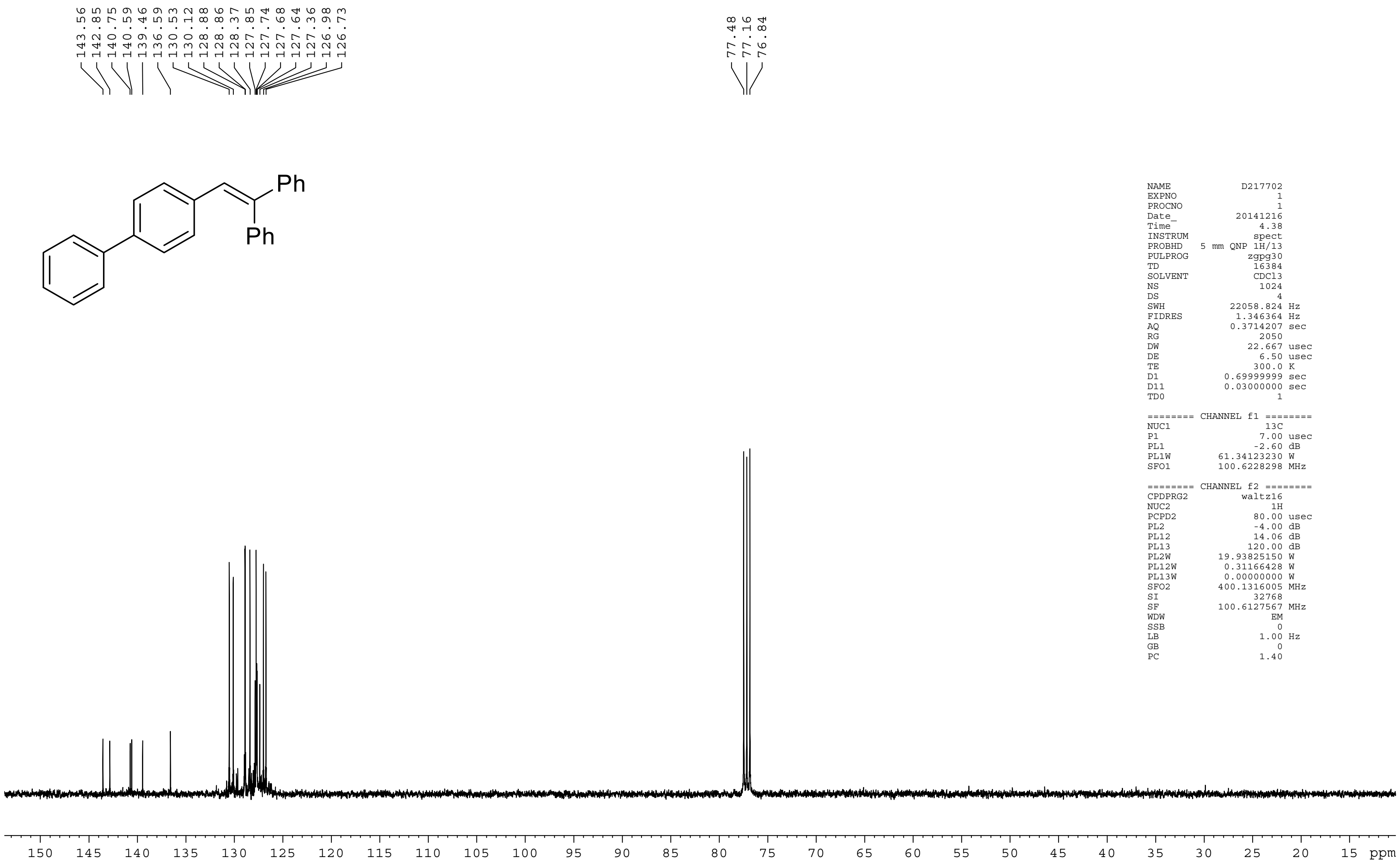

Figure S25. ^{13}C spectrum—compound **6f**.

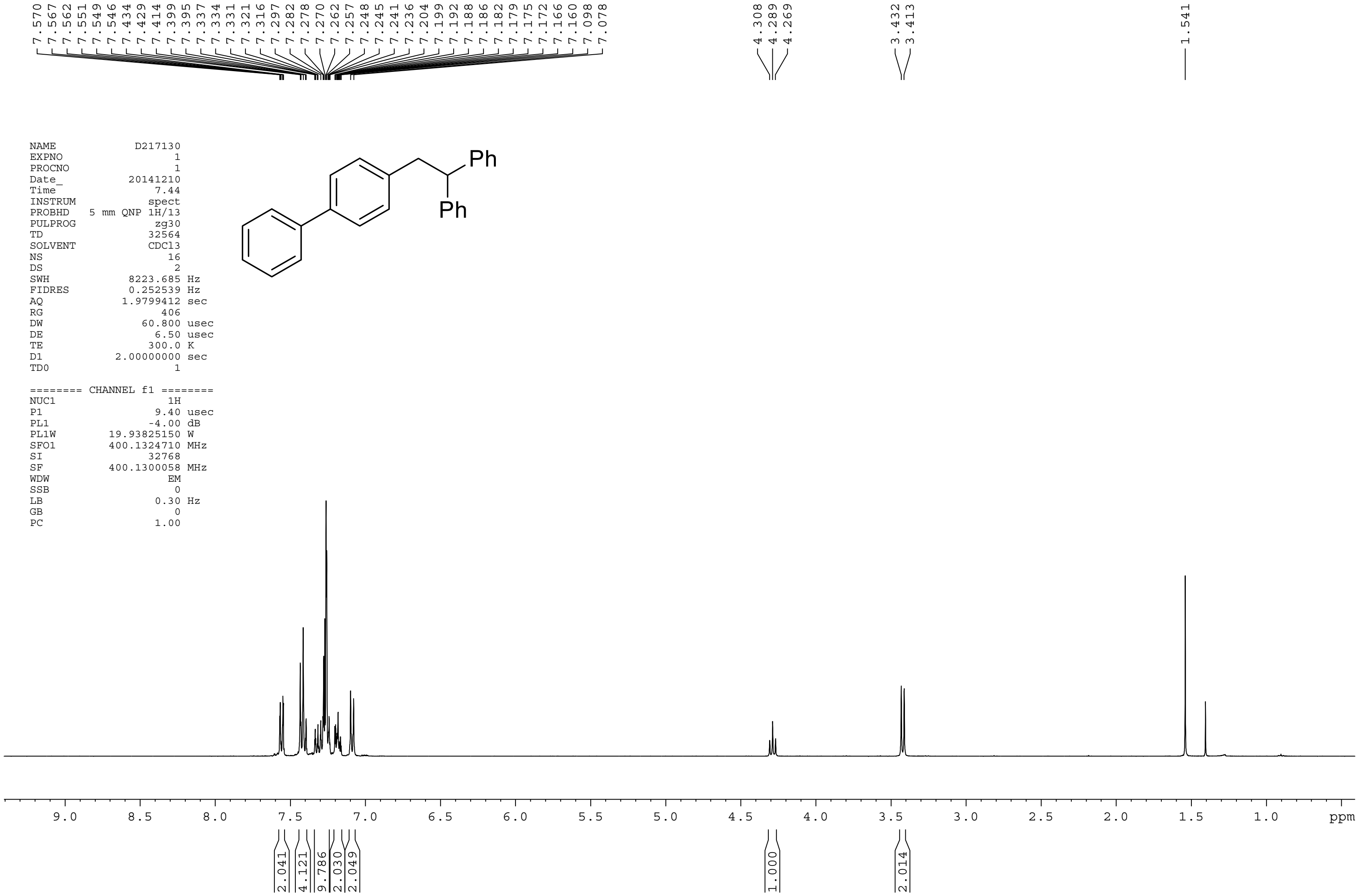

Figure S26. ^{1}H spectrum—compound **15f**.

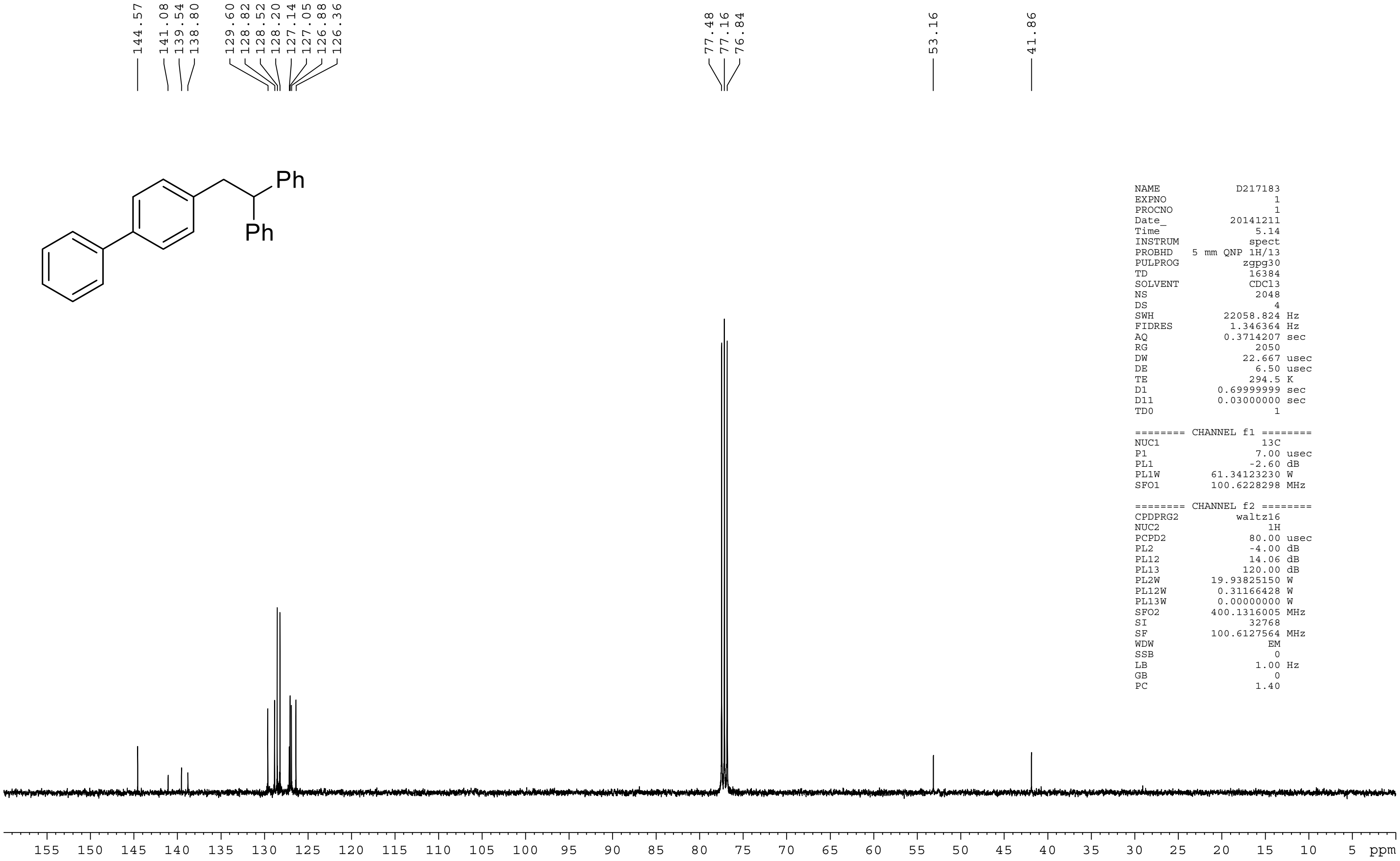

Figure S27. ^{13}C spectrum—compound **15f**.

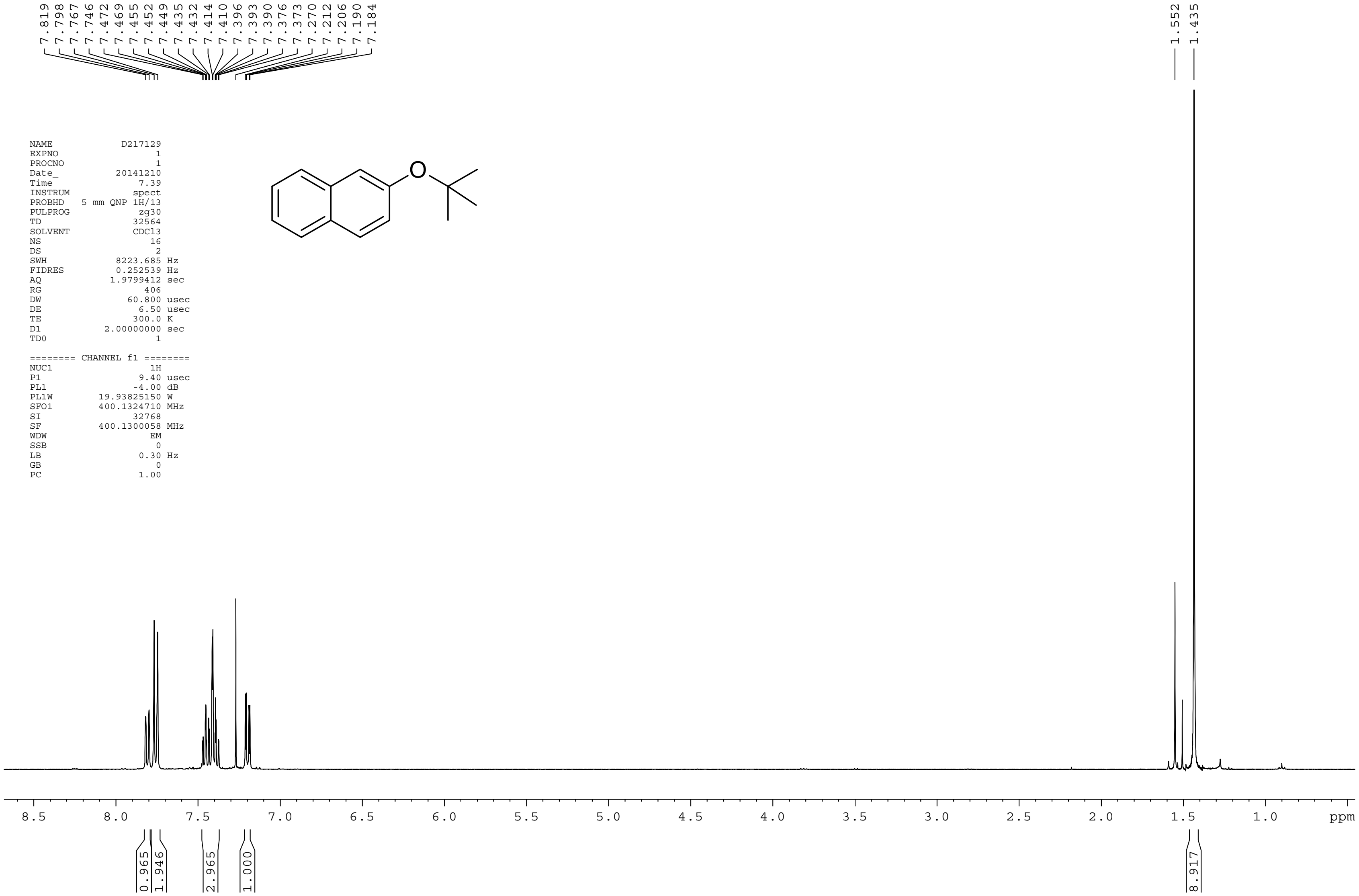

Figure S28. ^{1}H spectrum—compound **17**.

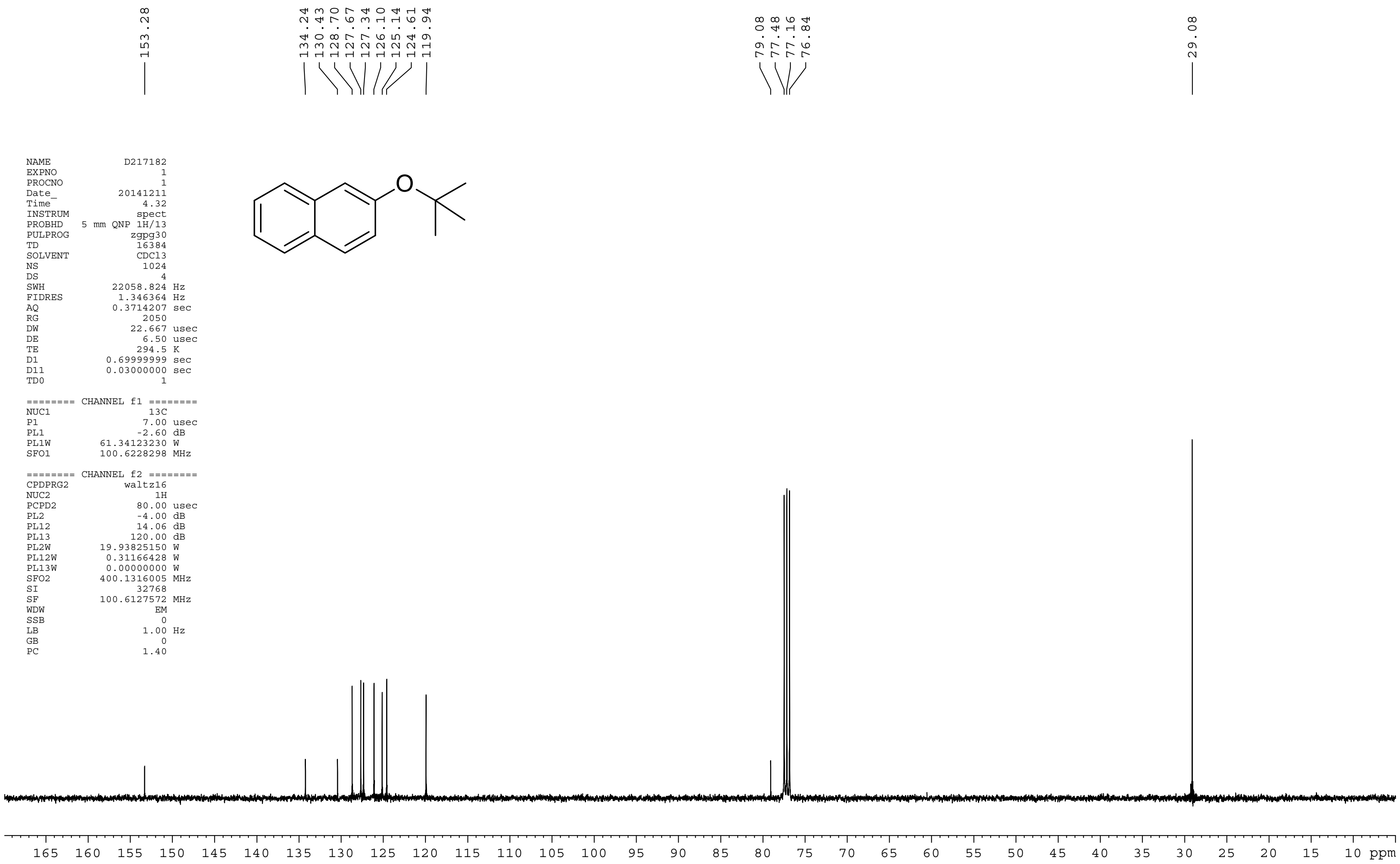

Figure S29. ^{13}C spectrum—compound **17**.

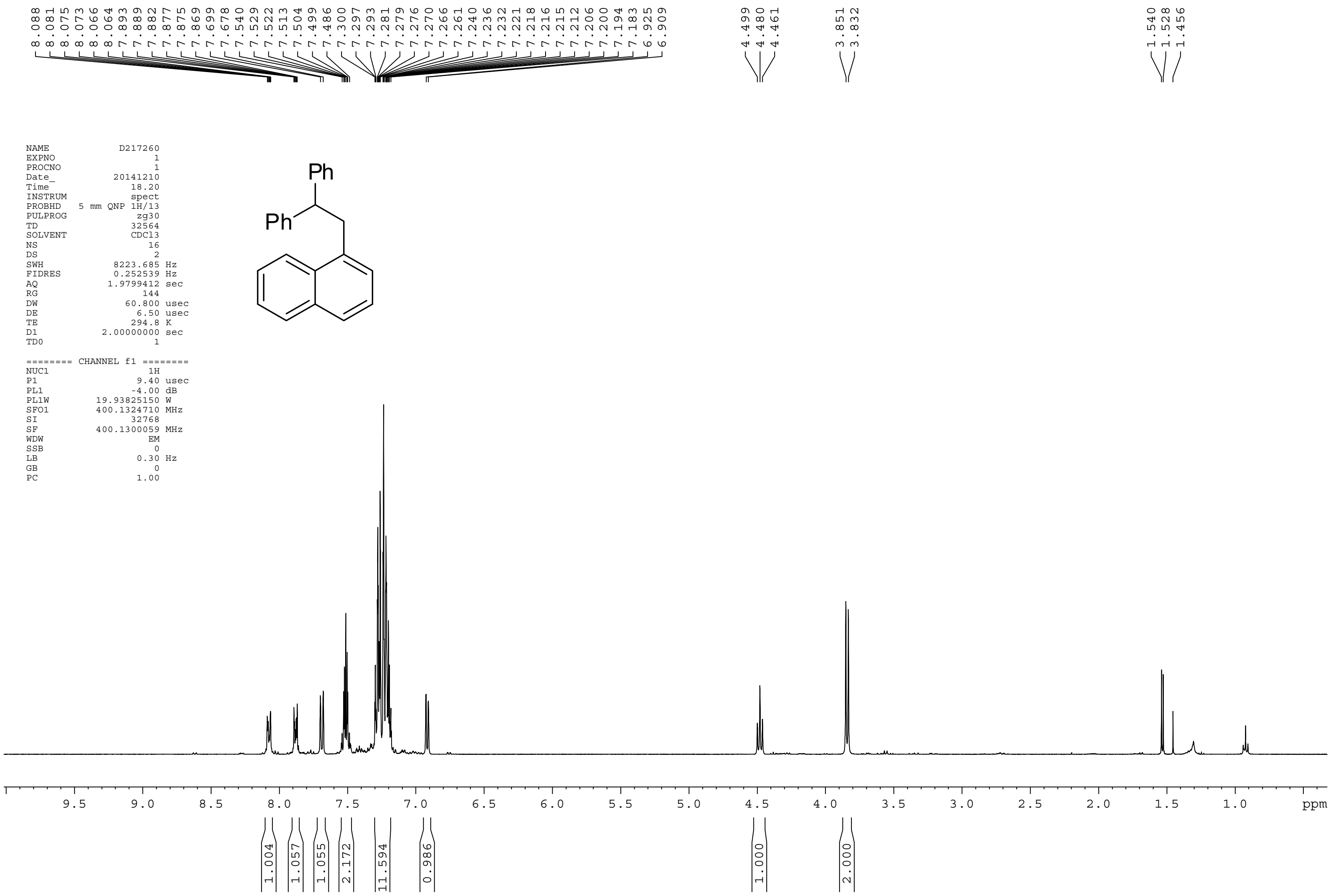

Figure S30. ^{1}H spectrum—compound **15g**.

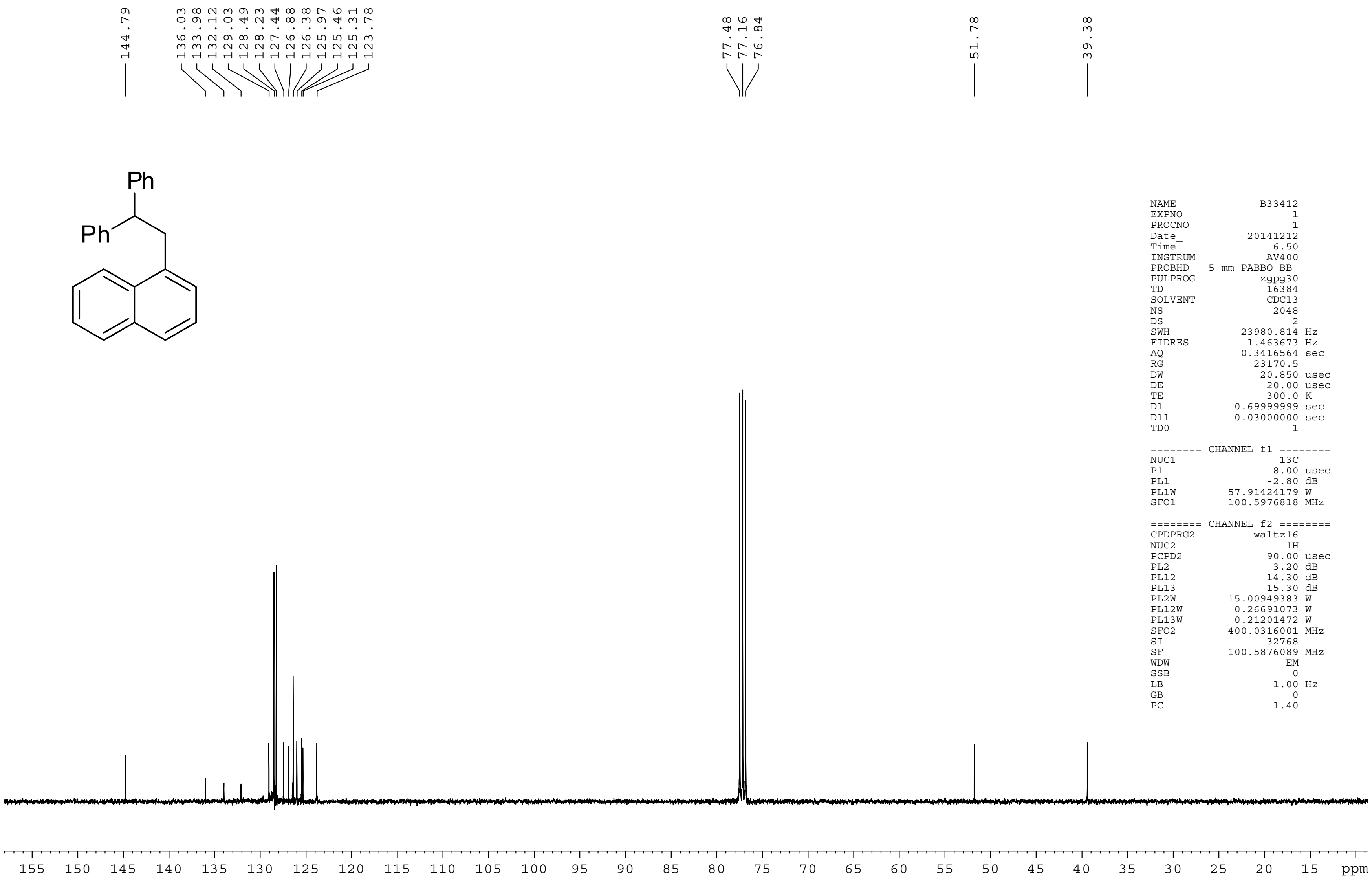

Figure S31. ^{13}C spectrum—compound **15g**.

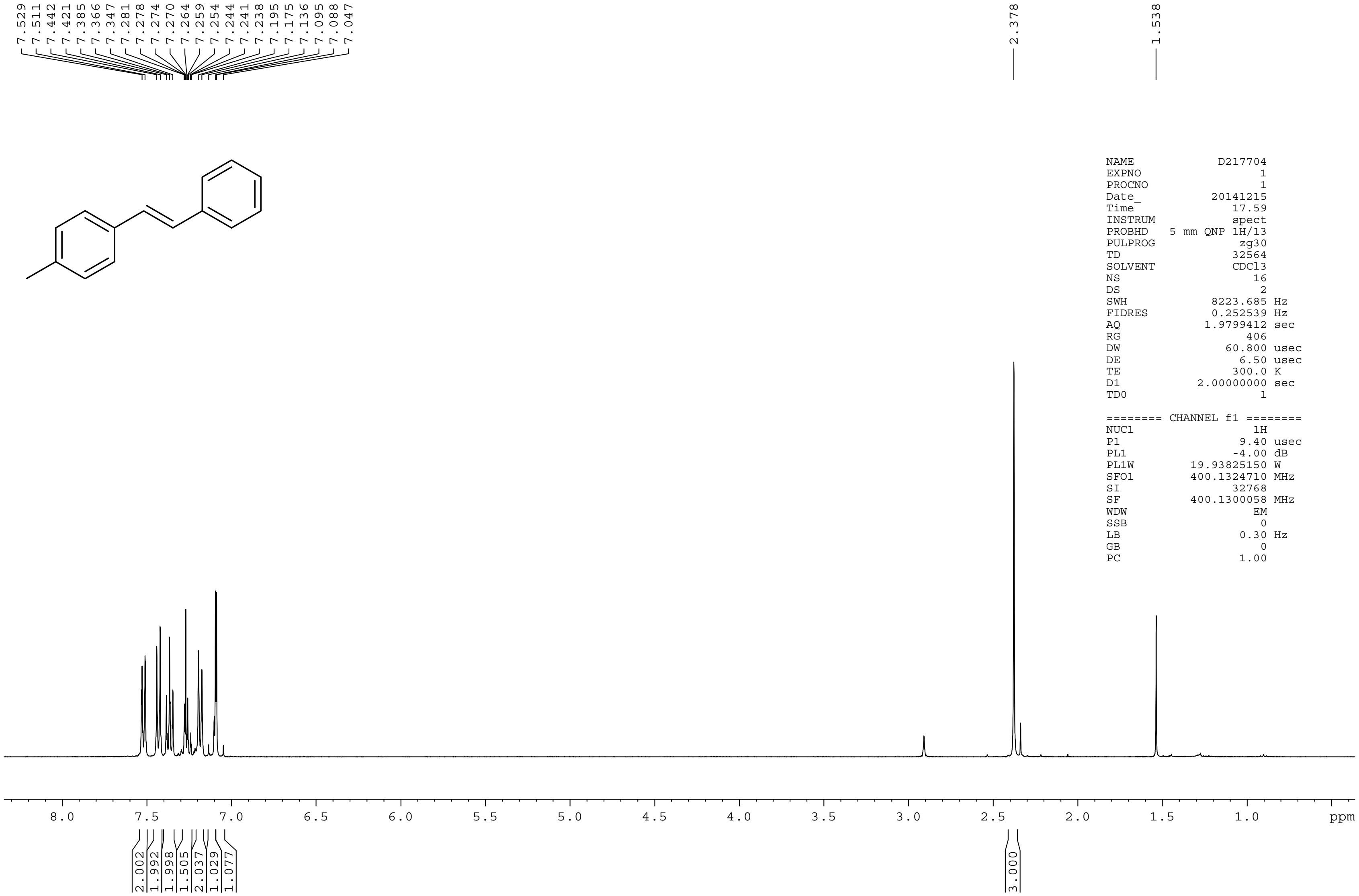

Figure S32. ^{1}H spectrum—compound **18b**.

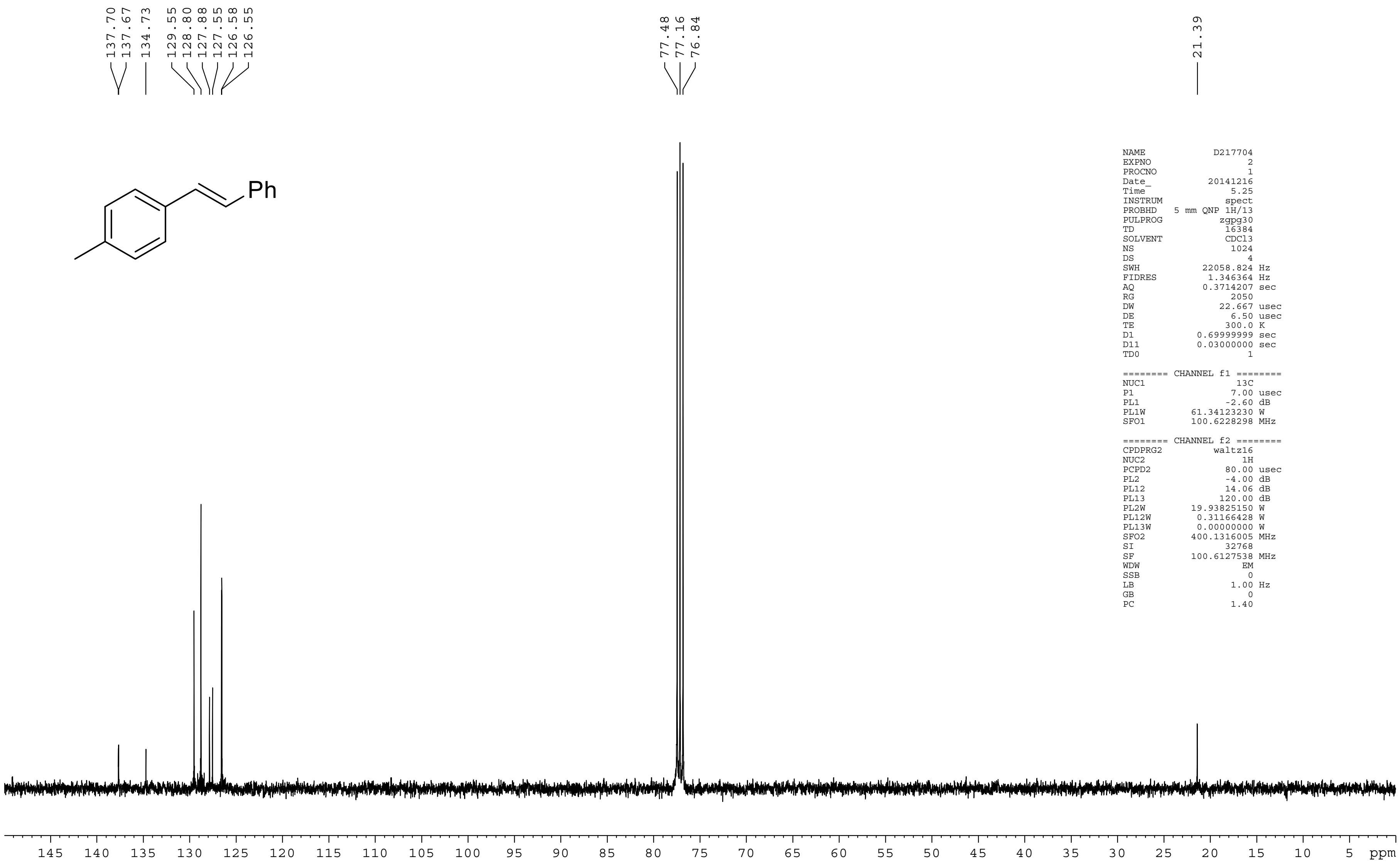

Figure S33. ^{13}C spectrum—compound **18b**.

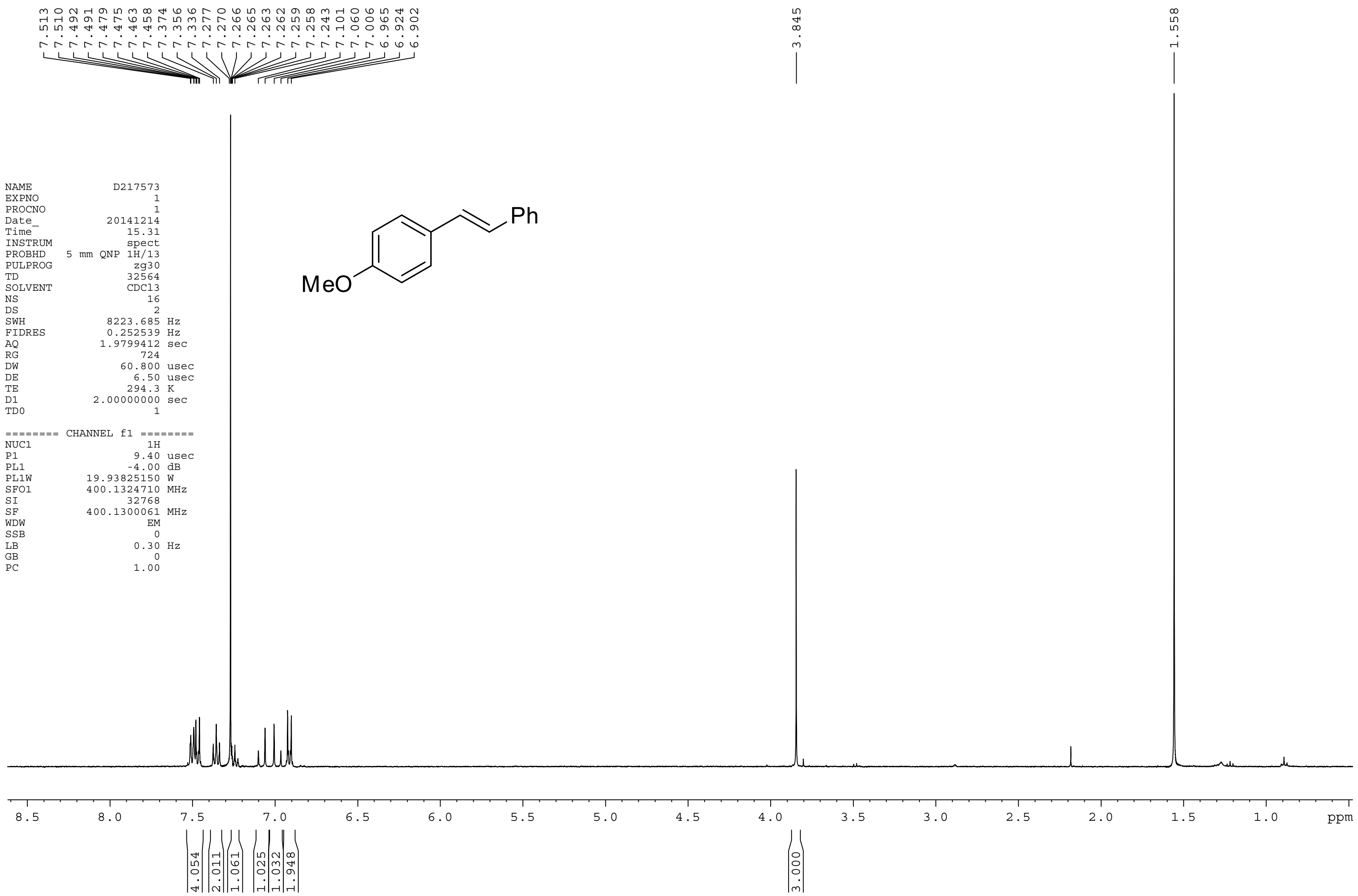

Figure S34. ^{1}H spectrum—compound **18c**.

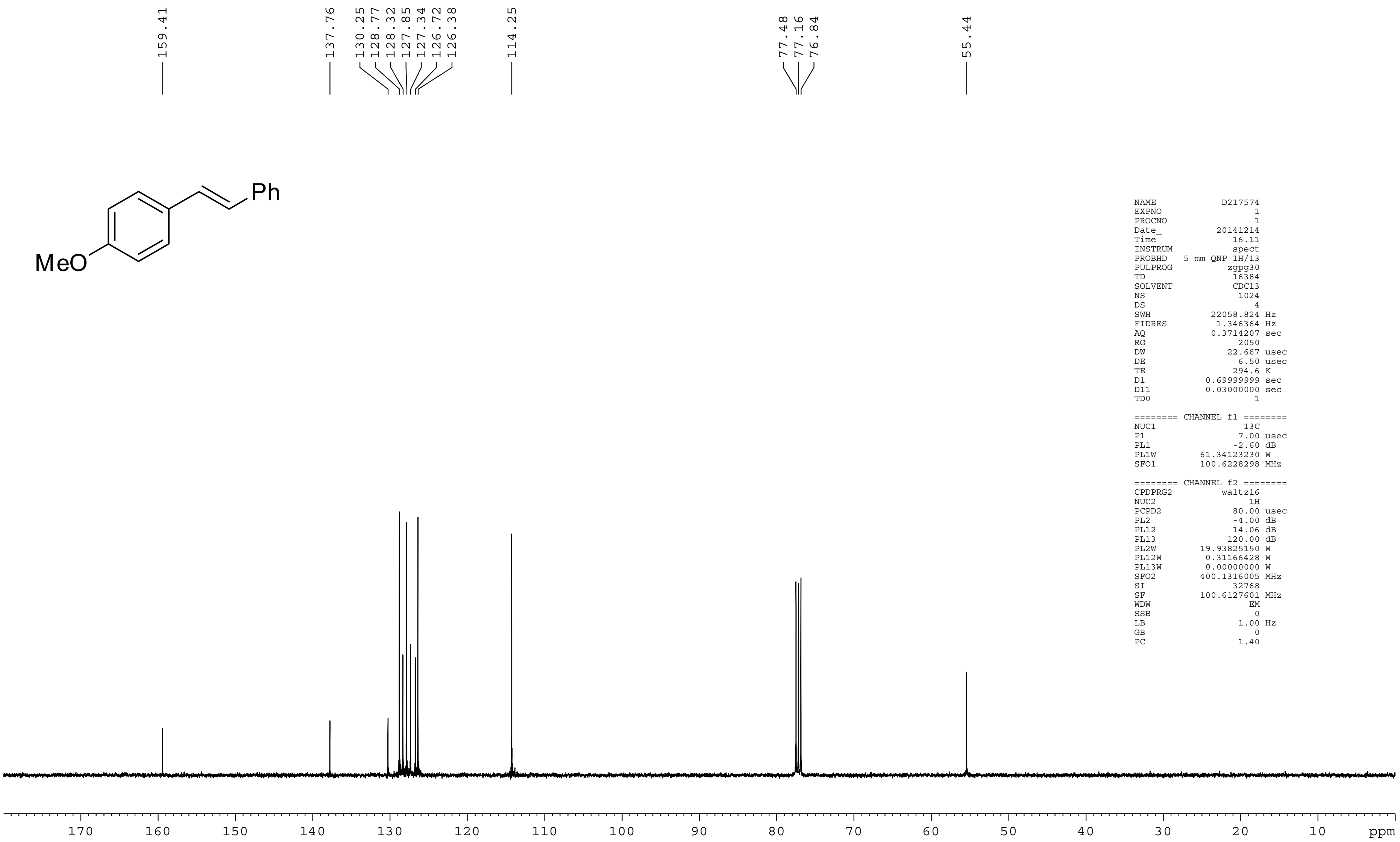

Figure S35. ^{13}C spectrum—compound **18c**.

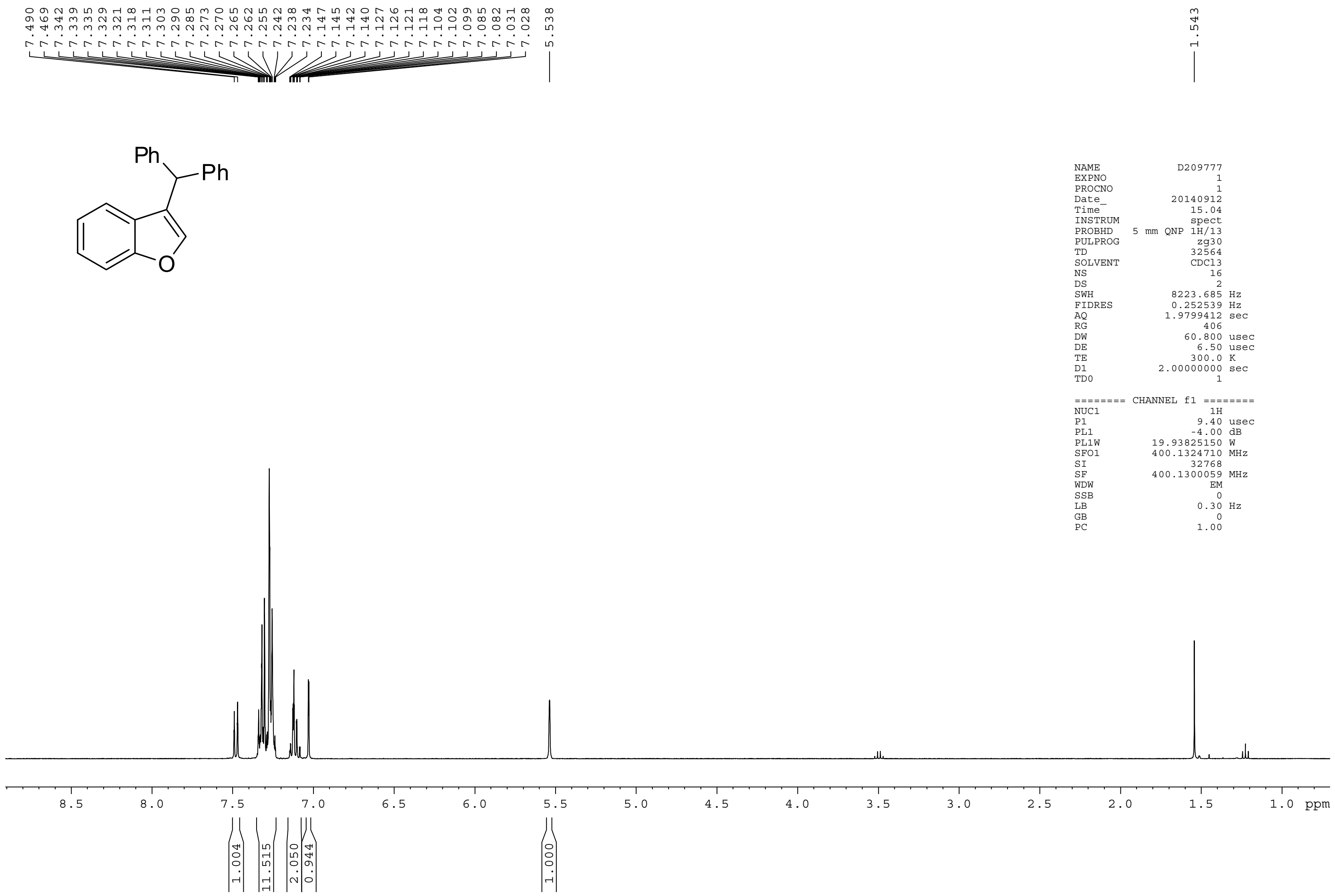

Figure S36. ^{1}H spectrum—compound **20**.

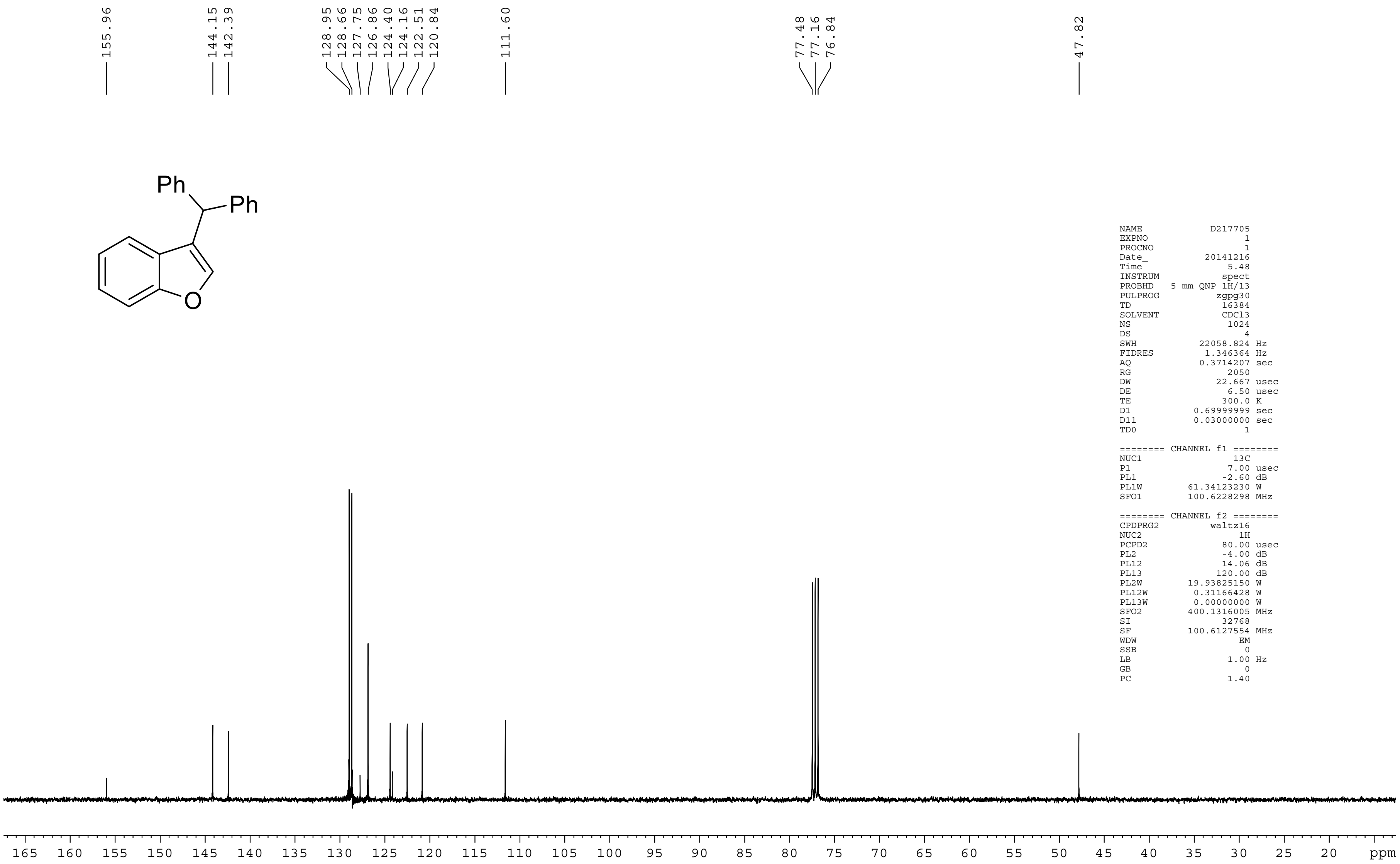

Figure S37. ^{13}C spectrum—compound **20**.

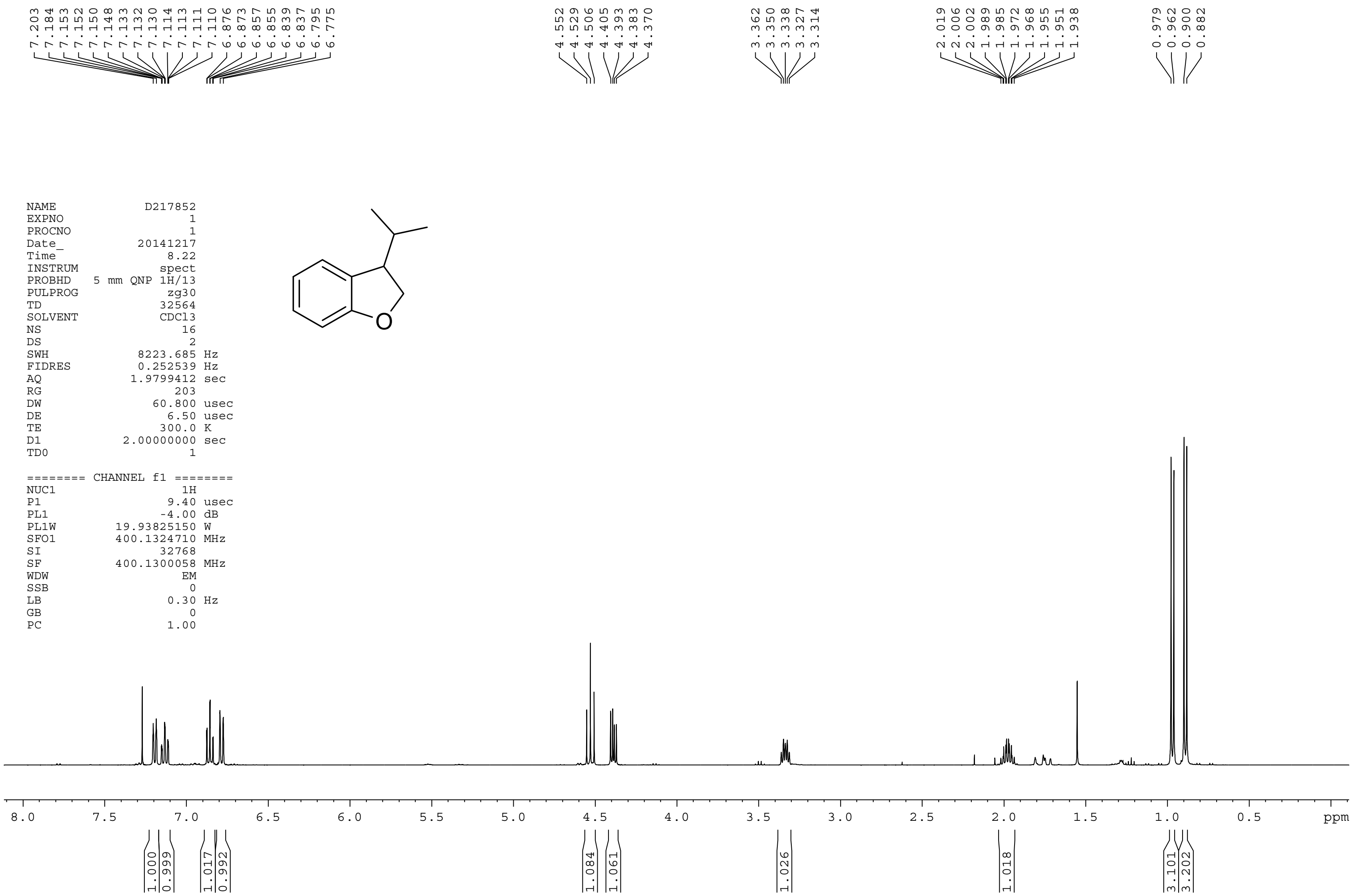

Figure S38. ^{1}H spectrum—compound **22**.

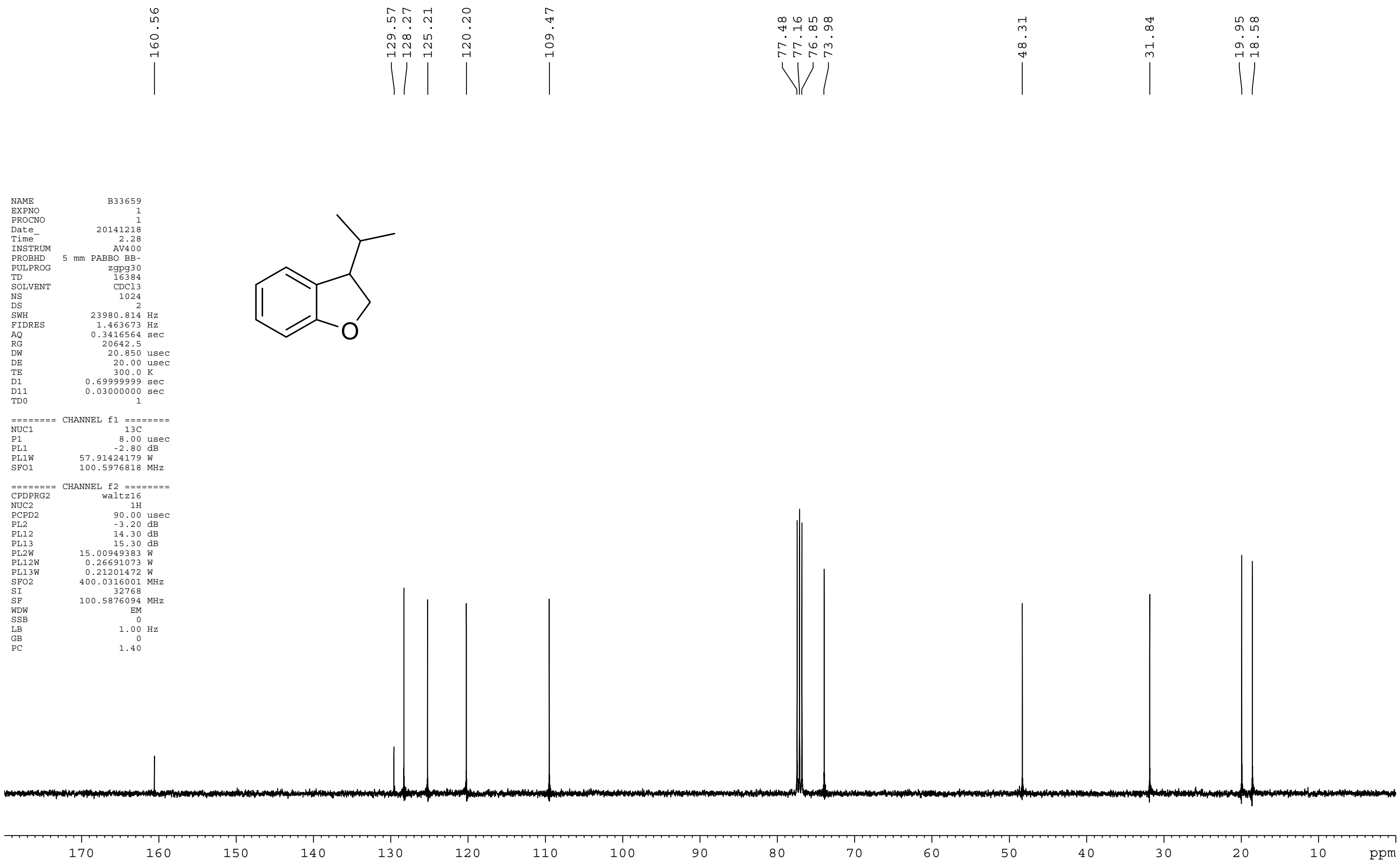

Figure S39. ^{13}C spectrum—compound **22**.

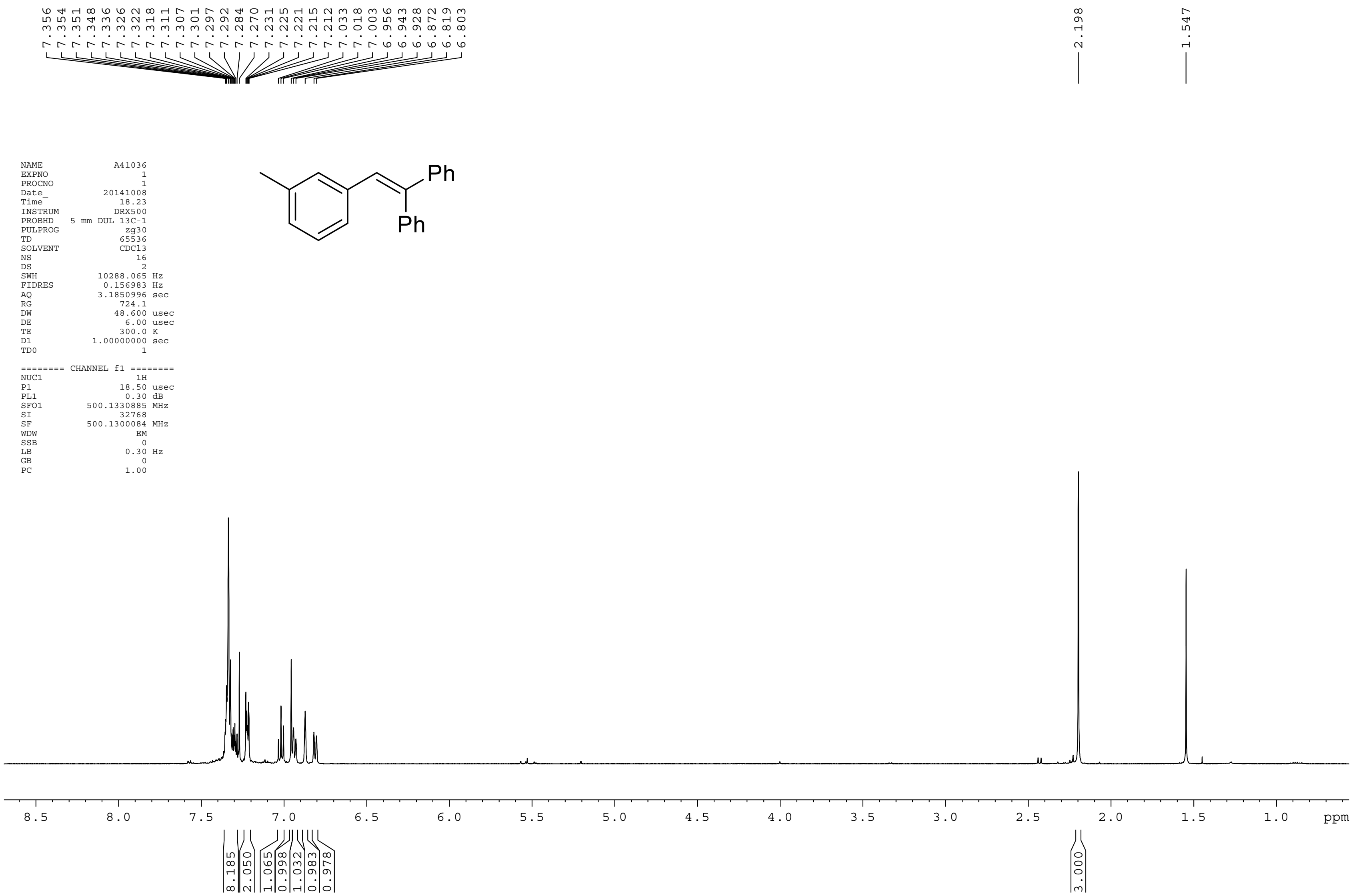

Figure S40. ^{1}H spectrum—compound **6h**.

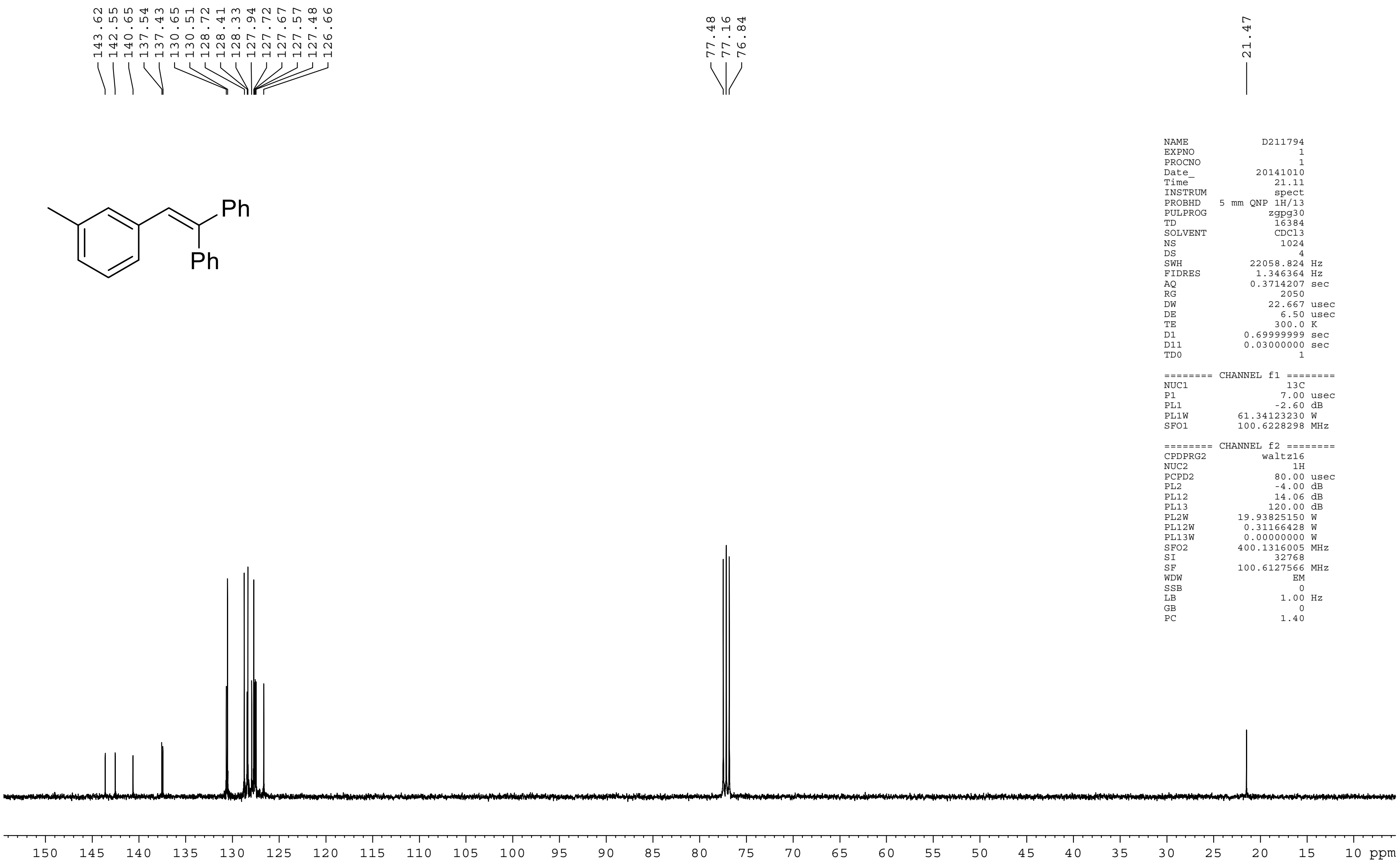

Figure S41. ^{13}C spectrum—compound **6h**.

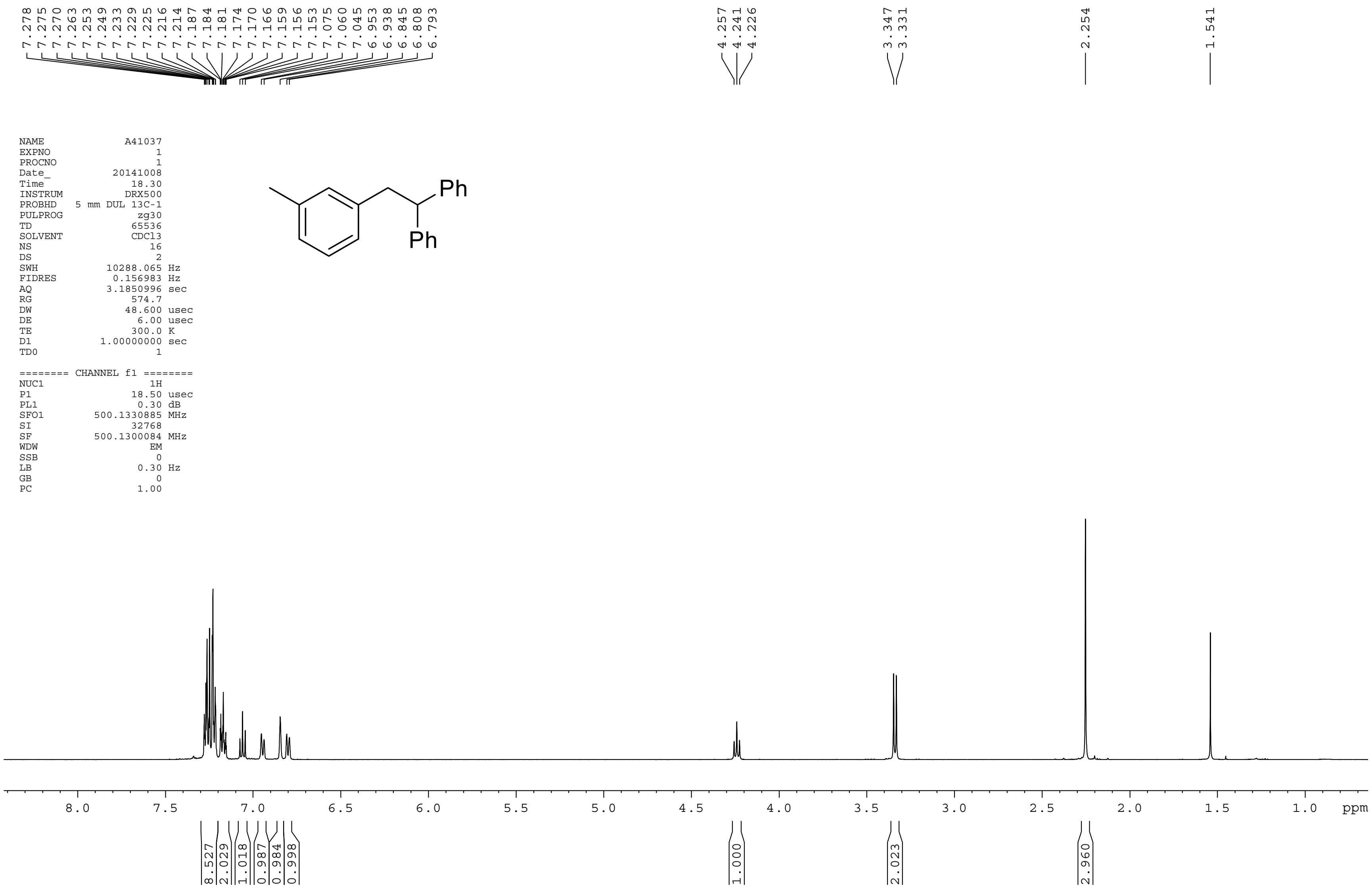

Figure S42. ^{1}H spectrum—compound **15h**.

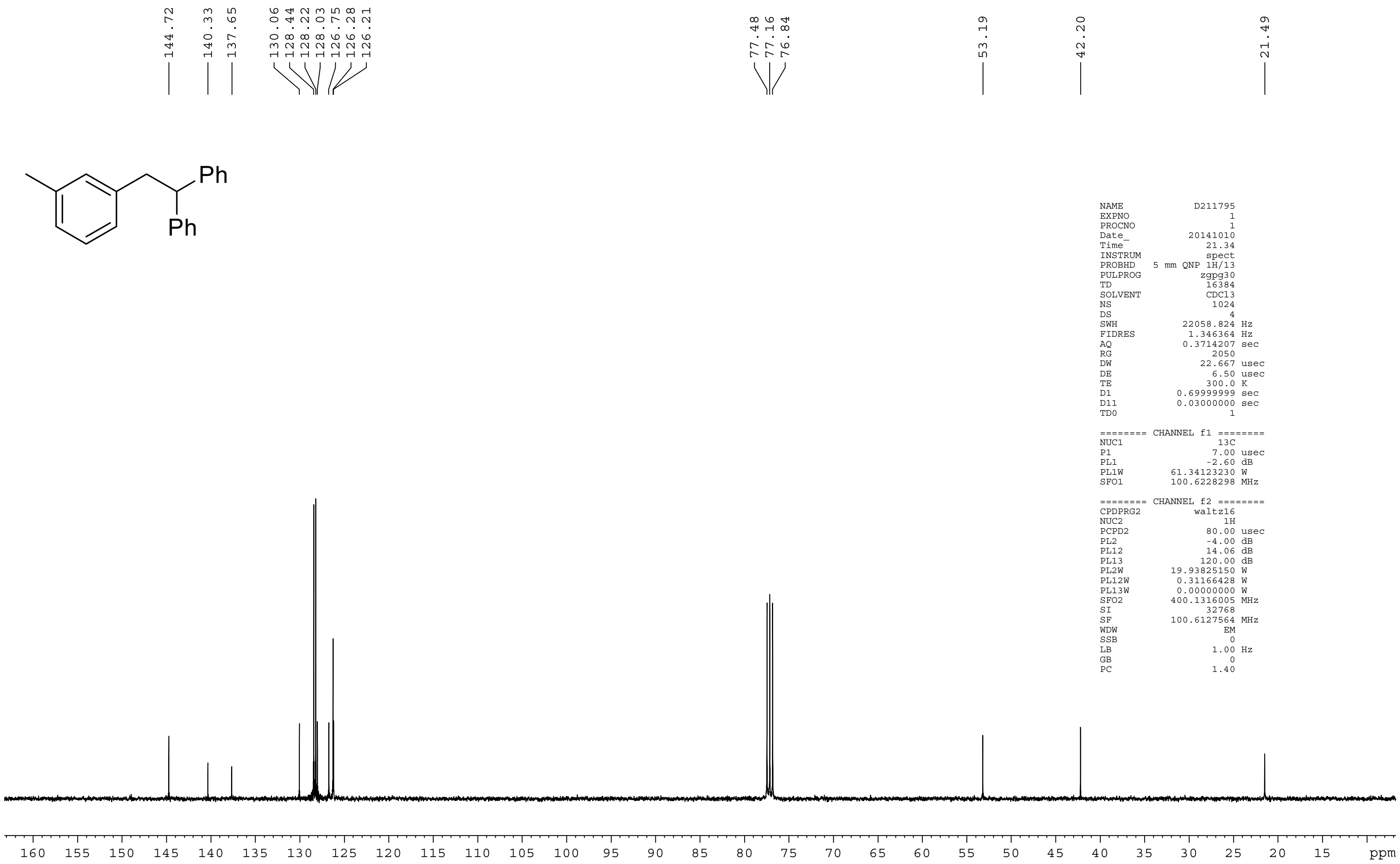

Figure S43. ^{13}C spectrum—compound **15h**.
